# The association between genetically elevated polyunsaturated fatty acids and risk of cancer

**DOI:** 10.1016/j.ebiom.2023.104510

**Published:** 2023-04-20

**Authors:** Fatty Acids in Cancer Mendelian Randomization Collaboration, Nathan Tintle, Terri Rice, Iona Cheng, Mark Jenkins, Steve Gallinger, Alex J. Cornish, Amit Sud, Jayaram Vijayakrishnan, Margaret Wrensch, Mattias Johansson, Aaron D. Norman, Alison Klein, Alyssa Clay-Gilmour, Andre Franke, Andres V. Ardisson Korat, Bill Wheeler, Björn Nilsson, Caren Smith, Chew-Kiat Heng, Ci Song, David Riadi, Elizabeth B. Claus, Eva Ellinghaus, Evgenia Ostroumova, Hosnijeh, Florent de Vathaire, Giovanni Cugliari, Giuseppe Matullo, Irene Oi-Lin Ng, Jeanette E. Passow, Jia Nee Foo, Jiali Han, Jianjun Liu, Jill Barnholtz-Sloan, Joellen M. Schildkraut, John Maris, Joseph L. Wiemels, Kari Hemminki, Keming Yang, Lambertus A. Kiemeney, Lang Wu, Laufey Amundadottir, Marc-Henri Stern, Marie-Christine Boutron, Mark Martin Iles, Mark P. Purdue, Martin Stanulla, Melissa Bondy, Mia Gaudet, Lenha Mobuchon, Nicola J. Camp, Pak Chung Sham, Pascal Guénel, Paul Brennan, Philip R. Taylor, Quinn Ostrom, Rachael Stolzenberg-Solomon, Rajkumar Dorajoo, Richard Houlston, Robert B. Jenkins, Sharon Diskin, Sonja I. Berndt, Spiridon Tsavachidis, Stephen J. Channock, Tabitha Harrison, Tessel Galesloot, Ulf Gyllensten, Vijai Joseph, Y. Shi, Wenjian Yang, Yi Lin, Stephen K. Van Den Eeden, Philip C. Haycock, Maria Carolina Borges, Kimberley Burrows, Rozenn N. Lemaitre, Stephen Burgess, Nikhil K. Khankari, Konstantinos K. Tsilidis, Tom R. Gaunt, Gibran Hemani, Jie Zheng, Therese Truong, Brenda M. Birmann, Tracy OMara, Amanda B. Spurdle, Mark M. Iles, Matthew H. Law, Susan L. Slager, Fatemeh Saberi Hosnijeh, Daniela Mariosa, Michelle Cotterchio, James R. Cerhan, Ulrike Peters, Stefan Enroth, Puya Gharahkhani, Loic Le Marchand, Ann C. Williams, Robert C. Block, ACCC, CCFR-CORECT-GECCO, EPITHYR, InterLymph, MMAC, ECAC, ILCCO, The PRACTICAL Consortium, PanScan, PanC4, Christopher I. Amos, Rayjean J. Hung, Wei Zheng, Marc J. Gunter, George Davey Smith, Caroline Relton, Richard M. Martin

**Affiliations:** a MRC Integrative Epidemiology Unit (IEU), Population Health Sciences, University of Bristol, Bristol, United Kingdom; b Department of Medicine, Cardiovascular Health Research Unit, University of Washington, Seattle, WA, USA; c MRC Biostatistics Unit, University of Cambridge, USA; d Division of Genetic Medicine, Department of Medicine, Vanderbilt University Medical Center, Nashville, TN, USA; e Department of Epidemiology and Biostatistics, School of Public Health, Imperial College London, London, UK; f Department of Hygiene and Epidemiology, University of Ioannina School of Medicine, Ioannina, Greece; g Department of Endocrine and Metabolic Diseases, Shanghai Institute of Endocrine and Metabolic Diseases, Ruijin Hospital, Shanghai Jiao Tong University School of Medicine, Shanghai, China; h Shanghai National Clinical Research Center for Metabolic Diseases, Key Laboratory for Endocrine and Metabolic Diseases of the National Health Commission of the PR China, Shanghai National Center for Translational Medicine, Ruijin Hospital, Shanghai Jiao Tong University School of Medicine, Shanghai, China; i Université Paris-Saclay, UVSQ, Inserm, Gustave Roussy, Team "Exposome, Heredity, Cancer and Health", CESP, Villejuif, France; j Channing Division of Network Medicine, Department of Medicine, Brigham and Women's Hospital and Harvard Medical School, Boston, MA, USA; k Genetics and Computational Biology Division, QIMR Berghofer Medical Research Institute, Brisbane, Queensland, Australia; l School of Medicine, Faculty of Health Sciences, University of Queensland, Australia; m Statistical Genetics, QIMR Berghofer Medical Research Institute, Brisbane, Australia; n School of Biomedical Sciences, Faculty of Health, and Institute of Health and Biomedical Innovation, Queensland University of Technology, Kelvin Grove, Queensland, Australia; o Department of Quantitative Health Sciences, Mayo Clinic, Rochester, MN, USA; p Institute for Risk Assessment Sciences, Utrecht University, Netherlands; q Genomic Epidemiology Branch, International Agency for Research on Cancer (IARC/WHO), Lyon, France; r Dalla Lana School of Public Health, University of Toronto, Canada; s Prevention and Cancer Control, Cancer Care Ontario, Ontario Health, Toronto, ON, Canada; t Public Health Sciences Division, Fred Hutchinson Cancer Center, Seattle, USA; u Department of Epidemiology, School of Public Health, University of Washington, Seattle, USA; v Department of Immunology, Genetics, and Pathology, Biomedical Center, Science for Life Laboratory (SciLifeLab) Uppsala, Uppsala University, Uppsala, Sweden; w Statistical Genetics Lab, QIMR Berghofer Medical Research Institute, 300 Herston Road, Herston QLD, 4006, Australia; x University of Hawaii Cancer Center, Honolulu, HI, USA; y School of Cellular and Molecular Medicine, University of Bristol, Bristol, UK; z Department of Public Health Sciences, University of Rochester, NY, USA; aa Dan L Duncan Comprehensive Cancer Center Baylor College of Medicine, USA; ab Lunenfeld-Tanenbaum Research Institute Mount Sinai Hospital and University of Toronto, Canada; ac Division of Epidemiology, Department of Medicine, Vanderbilt University Medical Center, Nashville, TN, USA; ad Section of Nutrition and Metabolism, International Agency for Research on Cancer (IARC), 150 Cours Albert Thomas, Lyon, France; ae The National Institute for Health Research (NIHR) Bristol Biomedical Research Centre, University Hospitals Bristol and Weston NHS Foundation Trust and University of Bristol, Bristol, United Kingdom; af Population Health Sciences, Bristol Medical School, University of Bristol, Bristol, United Kingdom; ag Leeds Institute for Data Analytics, University of Leeds, Leeds, UK; ah NIHR Leeds Biomedical Research Centre, Leeds Teaching Hospitals NHS Trust, Leeds, UK

**Keywords:** Mendelian randomization, Cancer risk, Polyunsaturated fatty acids, Omega 3, Omega 6, Delta-5 desaturase, Delta-6 desaturase

## Abstract

**Background:**

The causal relevance of polyunsaturated fatty acids (PUFAs) for risk of site-specific cancers remains uncertain.

**Methods:**

Using a Mendelian randomization (MR) framework, we assessed the causal relevance of PUFAs for risk of cancer in European and East Asian ancestry individuals. We defined the primary exposure as PUFA desaturase activity, proxied by rs174546 at the *FADS* locus. Secondary exposures were defined as omega 3 and omega 6 PUFAs that could be proxied by genetic polymorphisms outside the *FADS* region. Our study used summary genetic data on 10 PUFAs and 67 cancers, corresponding to 562,871 cases and 1,619,465 controls, collected by the Fatty Acids in Cancer Mendelian Randomization Collaboration. We estimated odds ratios (ORs) for cancer per standard deviation increase in genetically proxied PUFA exposures.

**Findings:**

Genetically elevated PUFA desaturase activity was associated (P < 0.0007) with higher risk (OR [95% confidence interval]) of colorectal cancer (1.09 [1.07–1.11]), esophageal squamous cell carcinoma (1.16 [1.06–1.26]), lung cancer (1.06 [1.03–1.08]) and basal cell carcinoma (1.05 [1.02–1.07]). There was little evidence for associations with reproductive cancers (OR = 1.00 [95% CI: 0.99–1.01]; P_heterogeneity_ = 0.25), urinary system cancers (1.03 [0.99–1.06], P_heterogeneity_ = 0.51), nervous system cancers (0.99 [0.95–1.03], P_heterogeneity_ = 0.92) or blood cancers (1.01 [0.98–1.04], P_heterogeneity_ = 0.09). Findings for colorectal cancer and esophageal squamous cell carcinoma remained compatible with causality in sensitivity analyses for violations of assumptions. Secondary MR analyses highlighted higher omega 6 PUFAs (arachidonic acid, gamma-linolenic acid and dihomo-gamma-linolenic acid) as potential mediators. PUFA biosynthesis is known to interact with aspirin, which increases risk of bleeding and inflammatory bowel disease. In a phenome-wide MR study of non-neoplastic diseases, we found that genetic lowering of PUFA desaturase activity, mimicking a hypothetical intervention to reduce cancer risk, was associated (P < 0.0006) with increased risk of inflammatory bowel disease but not bleeding.

**Interpretation:**

The PUFA biosynthesis pathway may be an intervention target for prevention of colorectal cancer and esophageal squamous cell carcinoma but with potential for increased risk of inflammatory bowel disease.

**Funding:**

Cancer Resesrch UK (C52724/A20138, C18281/A19169). 10.13039/501100000265UK Medical Research Council (MR/P014054/1). 10.13039/501100000272National Institute for Health Research (NIHR202411). 10.13039/501100000265UK Medical Research Council (MC_UU_00011/1, MC_UU_00011/3, MC_UU_00011/6, and MC_UU_00011/4). 10.13039/100000054National Cancer Institute (R00 CA215360). 10.13039/100000002National Institutes of Health (U01 CA164973, R01 CA60987, R01 CA72520, U01 CA74806, R01 CA55874, U01 CA164973 and U01 CA164973).

**Research in context**

**Evidence before this study**

Most meta-analyses of observational studies support the existence of protective associations between omega 3 polyunsaturated fatty acids (PUFAs) and cancer risk, whereas associations with omega 6 PUFAs are unclear. In randomized controlled trials (RCTs), there is little evidence for benefit from interventions on omega 3 or omega 6 PUFAs but some evidence that increased total PUFA intake might increase cancer risk (see supplement for literature search strategy). The current evidence base is subject to limitations, including measurement error and study heterogeneity in the observational data and the potential influence of high background PUFA intake and short follow-up time in RCTs. RCTs have also focused primarily on overall cancer incidence, with little evidence on site-specific cancers.

**Added value of this study**

We addressed the continuing uncertainty on the causal relevance of PUFAs for risk of site-specific cancers through a Mendelian randomization study design, an approach that exploits natural randomization of germline genotypes to strengthen causal inference in observational studies. We used summary genetic data on 67 cancers in European and East Asian ancestry studies, corresponding to 562,871 cases and 1,619,465 controls, collected by the Fatty Acids in Cancer Mendelian Randomization Collaboration. We found robust genetic evidence compatible with a causal effect of increased PUFA biosynthesis on risk of colorectal cancer and esophageal squamous cell carcinoma, with little evidence for associations with male or female reproductive cancers, blood cancers, urinary system cancers or nervous system cancers. Further analyses highlighted omega 6 PUFAs, such as arachidonic acid, as potential mediators of findings for colorectal cancer and identified increased risk of inflammatory bowel disease as a potential consequence of interventions to inhibit PUFA biosynthesis.

**Implications of all the available evidence**

Dietary guidelines typically recommend replacement of saturated with polyunsaturated fat for prevention of coronary heart disease. Our findings suggest, however, that such advice should potentially be reconsidered in individuals with an increased risk of colorectal cancer. Our findings are compatible with RCT evidence that interventions on PUFAs have little to no effect on overall cancer incidence but is beneficial for prevention of colorectal adenomas, a precursor for colorectal cancer. Taken together, our findings support the design of trials to evaluate the role of interventions on omega 6 PUFAs for colorectal cancer prevention but highlight inflammatory bowel disease as a potential adverse effect.

## Introduction

Polyunsaturated fatty acids (PUFAs) are substantial components of the diet, contributing about 4–11% of total energy intake in Europe.^1^ The most important PUFA classes are the omega 3 family, derived from alpha-linolenic acid, and the omega 6 family, derived from linoleic acid (LA). Omega 3 and omega 6 PUFAs are metabolized by delta-5 (D5D) and delta-6 (D6D) desaturases to their respective long-chain metabolites. PUFAs are important precursors for eicosanoid hormones and regulate several processes implicated in cancer and other diseases, including inflammation, thrombosis and insulin resistance.^2^^,^^3^

Most meta-analyses of observational studies support the existence of protective associations between omega 3 PUFAs and cancer risk,^4^, ^5^, ^6^, ^7^, ^8^, ^9^, ^10^, ^11^, ^12^, ^13^ whereas associations with omega 6 PUFAs are unclear.^11^^,^^14^^,^^15^ In randomized controlled trials (RCTs), there is little evidence for benefit from interventions on omega 3 or omega 6 PUFAs but some evidence that increased total PUFA intake might increase cancer risk.^16^, ^17^, ^18^

Interpretation of the observational evidence is undermined by study heterogeneity and other limitations. For example, the correlation between dietary-recall methods and fatty acid biomarkers is weak-to-modest (ranging from 0.12 to 0.37),^19^, ^20^, ^21^ suggesting substantial scope for measurement error. In two meta-analyses, biomarker and dietary recall-based studies tended to give results in opposite directions.^22^^,^^23^ Increasing the scope for study heterogeneity, fatty acid measurements in different tissues capture different temporal patterns of intake.^15^ Null findings from RCTs could reflect the impact of high background intake of supplements or fish (major sources of omega 3 PUFAs), short follow-up times and the possible importance of exposure before middle age (the typical age of study participants). Previous RCTs have also focused primarily on omega 3 PUFAs and overall cancer incidence, with little evidence on site-specific cancers.

We addressed the continuing uncertainty on the causal relevance of PUFAs for risk of site-specific cancers through a Mendelian randomization (MR) study design, defining the primary exposure as PUFA desaturase activity and secondary exposures as individual omega 3 and omega 6 PUFAs. The MR approach exploits natural randomization of germline genotypes to strengthen causal inference in observational studies^24^ and addresses many of the aforementioned limitations. For example, germline genetic variation can be used to model lifelong exposure, avoiding limitations due to short follow-up time or long exposure latencies, and can be measured with high accuracy, conferring less susceptibility to the measurement error biases of dietary studies. Additionally, germline genetic variants are fixed, and their distribution is generally random in the population with respect to socioeconomic or environmental confounders, meaning that MR findings are less susceptible to reverse causation and confounding seen in observational studies. Subject to satisfaction of the instrument variable assumptions, estimates derived from MR can be interpreted as causal.^24^ Our study made use of summary data generated in genome-wide association studies (GWAS) of 10 PUFAs and 67 cancers, corresponding with up to 562,871 cases and 1,619,465 controls and 43 consortia or biobanks, collected by the Fatty Acids in Cancer Mendelian Randomization Collaboration (FAMRC).^25^

## Methods

Our study had six design components (Supplementary Fig. S1): 1) definition of PUFA exposures; 2) design of instruments for PUFA exposures; 3) MR analyses of PUFAs and cancer risk; 4) sensitivity analyses for violations of assumptions; 5) modelling to identify sources of heterogeneity in findings amongst cancers; and 6) a phenome-wide MR study (MR-PheWAS) of non-neoplastic diseases to assess potential for adverse effects from interventions on PUFA biosynthesis.

### Definition of primary and secondary PUFAs

In the present study, we defined our primary PUFA exposure as activity of D5D and D6D, enzymes encoded by the *FADS1* and *FADS2* genes. D5D and D6D catalyse rate-limiting desaturase steps in omega 6 and omega 3 PUFA biosynthesis (Supplementary Fig. S2) and are strong determinants of variation in most PUFAs. Defining our primary exposure in this way, rather than as individual PUFAs, had two key advantages. First, instruments designed for individual PUFAs will be largely driven by the *FADS* region, making corresponding MR results highly redundant. Second, our exposure choice makes it easier to justify MR assumptions because: i) the *FADS* region has a proven biological role in PUFA metabolism; and ii) MR studies conducted at the protein level are less susceptible to horizontal pleiotropy bias.^26^ We further defined a set of secondary exposures as omega 3 and omega 6 PUFAs that could be instrumented by variation outside the *FADS* region.

### Design of instrument for primary exposure

We used the following product-to-substrate ratios as biomarkers of enzyme activity: for D5D, the ratio of arachidonic acid to dihomo-gamma-linolenic acid (AA:DGLA) and for D6D, the ratio of gamma-linolenic acid to LA (GLA:LA) (Supplementary Fig. S1). For analyses of European ancestry individuals, we derived summary data for AA:DGLA and GLA:LA by applying GWIS^27^ (Genome-Wide Inferred Statistics for Functions of Multiple Phenotypes) to summary data for AA, DGLA, GLA and LA obtained from the Cohorts for Heart and Aging Research in Genomic Epidemiology study (N = 8631).^28^ For analyses of East Asian ancestry individuals, we obtained summary data for AA:DGLA and GLA:LA from the Singapore Chinese Health Study (N = 1316).^29^

We identified single nucleotide polymorphisms (SNPs) associated with the D5D and D6D activity biomarkers using a conventional threshold of GWAS statistical significance (P < 5 × 10^−8^) and with linkage disequilibrium (LD) clumping to prune for independence. This identified rs174546 as the most strongly associated and only independent variant for AA:DGLA (standard deviation [SD] change per C allele = 0.87 [standard error = 0.01]; r^2^ = 0.33, P < 5 × 10^−100^) and GLA:LA (SD change per C allele = 0.38 [0.02]; r^2^ = 0.06, P < 5 × 10^−100^) in Europeans and GLA:LA (SD change per C allele = 0.72 [0.03]; r^2^ = 0.23, P < 5 × 10^−100^) in East Asians (Supplementary Table S1). No associations were identified for AA:DGLA in East Asians (P = 0.11). Since rs174546 is associated with both D5D and D6D activity biomarkers in European ancestry individuals, we interpret rs174546 as an instrument for PUFA desaturase activity. See the Supplementary methods for further details on the primary instrument.

### Design of instruments for secondary exposures

To identify instruments for secondary exposures (defined as omega 3 or omega 6 PUFAs that could be instrumented by variation outside the *FADS* region) we performed LD clumping on summary data for 14 PUFAs measured in six studies. When multiple studies were available for the same PUFA, we restricted analyses to the single largest study for that PUFA. Potential bias from this strategy, which we consider to be minimal, is discussed in the Supplementary methods. For studies of European ancestry, this identified 124 SNPs associated with 14 PUFAs. Four of the 14 PUFAs could not be instrumented by variation outside the FADS region and were excluded. The retained secondary exposures included five omega 3 PUFAs and five omega 6 PUFAs (variation explained, excluding the *FADS* region, ranged from 0.36% to 2.52% for omega 3 and 0.47% to 4.59% for omega 6). For studies of East Asian ancestry, only one PUFA was identified that could be instrumented by variation outside the *FADS* region. We therefore excluded studies of East Asian ancestry from secondary analyses. See Supplementary Tables S1–S3 and the Supplementary materials for further details on the PUFA exposures, their genetic instruments, and the instrument selection strategy.

### Mendelian randomization analyses of PUFAs and cancer risk

Summary data were available for 90 cancers derived from 51 studies, cleaned and harmonised by the FAMRC^25^ (Supplementary Table S4). For primary analyses we focused on 67 cancers with greater than 1000 cases and 1000 controls, derived from 43 studies (Supplementary Tables S5 and S6). Per SD increase in genetically proxied PUFA desaturase activity, we estimated we had ≥80% power to detect odds ratios (ORs) ≥1.05 for 22 cancers, ≥1.10 for 45 cancers and ≥1.15 for 63 cancers (alpha = 0.05).

We estimated the effect of the PUFA desaturase biomarker on cancer risk using the Wald ratio:

$${\hat{ß}}_{IV}=\frac{{\hat{ß}}_{ZY}}{{\hat{ß}}_{ZX}}$$

in which $\hat{\beta }$_ZY_ is the log OR for cancer (Y) due to rs174546 (Z) and $\hat{\beta }$_ZX_ is the SD change in PUFA desaturase activity (X) due to rs174546. The fatty acid ratios AA:DGLA and GLA:LA were used as biomarkers for PUFA desaturase activity in European and East Asian ancestry studies, respectively. $\hat{\beta }$_IV_ can be interpreted as the estimated log OR for cancer per SD increase in the PUFA desaturase biomarker due to rs174546, with variance estimated as the standard error for $\hat{\beta }$_ZY_ divided by $\hat{\beta }$_ZX_. When summary data were available for the same cancer from multiple independent studies, we conducted MR analyses separately for each study, and then combined the MR results by fixed effects meta-analysis using inverse variance weights (an alternative approach gave similar results; see supplementary materials). MR analyses were conducted using the TwoSampleMR R package (version 0.5.6),^30^ and we used a Bonferroni corrected alpha error threshold of 0.05/67 (0.0007) to identify associations. To boost power, we also combined MR results across cancers for selected biological systems (reproductive cancers, nervous system cancers, urinary cancers and blood cancers) using random effects meta-analysis, implemented in the meta package.^31^ Z and Cochrane's Q tests were used to assess differences in MR findings amongst selected cancers. Analyses made allowance for potential sample overlap between studies (Supplementary methods). To identify additional potential associations, we also searched for cancers associated with rs174546 (or LD proxies) in the GWAS catalog up until 19 January 2021.^32^

In secondary MR analyses of selected cancers, we further assessed evidence for associations with omega 3 and omega 6 PUFAs using instruments that were independent of the *FADS* region. For seven of 10 PUFAs with multiple instrumental SNPs, we estimated associations using inverse variance weighted (IVW) linear regression (the Wald ratio method was using for the remaining three PUFAs). The variance for the IVW effect was estimated using a random effects model, except when there were only two independent instrumental SNPs or there was under-dispersion in effect estimates,^33^^,^^34^ in which cases a fixed effects model was used. We used an alpha threshold of 0.05 to identify potential associations.

### Sensitivity analyses for violations of assumptions

Inference of causal effects in our estimates requires satisfaction of the following instrumental variable assumptions: (1) the selected SNPs are associated with the exposure; (2) the selected SNPs are not associated with confounders; and (3) the selected SNPs are associated with cancer exclusively through their effect on the exposure.^24^ If these assumptions are satisfied, the selected SNPs are valid instrumental variables, and an association between the exposure and cancer can be interpreted as causal. We conducted three sets of analyses to assess the sensitivity of our findings to violations of these assumptions (name of assumption in brackets): colocalisation analysis (assumption 2 or no genomic confounding), within-sibship MR analyses (assumption 2 or no confounding by population stratification) and effect decomposition analyses (assumption 3 or no horizontal pleiotropy bias) (details in Supplementary methods). Colocalisation analyses, which provide evidence against genomic confounding, were conducted using the coloc package^35^ and assessed evidence for sharing the same causal variant amongst cancer, PUFA desaturase activity (as proxied by the fatty acid ratios AA:DGLA or GLA:LA) and *FADS1* and *FADS2* gene expression at the *FADS* region. Within sibship MR analyses were conducted using data on 19,588 sibships from UK Biobank.^36^^,^^37^ In effect decomposition analyses, we estimated associations of rs174546 with 36 selected and biomedically important characteristics, including lipids and anthropometrics, and then modelled the extent to which any identified associations (defined as P values < 0.0013 [alpha of 0.05/36]) could explain our findings using the product of coefficients method.^38^

### Modelling sources of heterogeneity

To identify sources of heterogeneity in MR findings amongst cancers, we assessed the impact of cancer-level characteristics using a meta-regression approach^39^ (Supplementary methods). We modelled the following cancer-level characteristics: smoking (i.e. whether smoking is an accepted cause of the cancer^40^^,^^41^), chronic inflammation (whether the cancer has an accepted relationship to a chronic inflammatory condition^42^), cancer incidence,^43^ survival time,^43^ median age-at-diagnosis^43^ and tissue-specific rates of stem cell division.^44^ Analyses made allowance for sample overlap between studies and assessed the sensitivity of findings to alternative cancer groupings. We interpret findings from this approach as exploratory and used an alpha threshold of 0.05 to identify potential sources of heterogeneity.

### Phenome-wide Mendelian randomization study of non-neoplastic diseases

PUFA biosynthesis interacts with aspirin, which is known to increase risk of bleeding and inflammatory bowel disease. To identify possible adverse effects from hypothetical interventions on the PUFA biosynthesis pathway, we conducted an MR-PheWAS of non-neoplastic outcomes, using disease associations curated by OpenGWAS.^45^^,^^46^ Associations with non-neoplastic diseases were estimated using the same procedure described above for primary MR analyses. The selected non-neoplastic outcomes covered a wide range of disease areas, including autoimmune, inflammatory, bleeding, cardiometabolic, psychiatric, neurological, bone and connective tissue conditions. We used an alpha threshold of 0.05, with a Bonferroni correction for multiple testing (0.05/84 outcomes = 0.0006), to identify potential associations.

### Ethics

This work used summary data from previously published GWAS or summary data from GWAS conducted in UK Biobank under application number 15825. Relevant approvals were obtained by each of the previously published studies. An ethics statement for each included GWAS can be found in Supplementary Table S6. For GWAS conducted in UK Biobank under application number 15825, UK Biobank has obtained Research Tissue Bank (RTB) approval from its ethics committee that covers the majority of proposed uses of the Resource. The UK Biobank Research Ethics Committee (REC) approval number is 16/NW/0274.

### Role of funders

The funding institutions had no role in the design and conduct of the study; collection, management, analysis, and interpretation of the data; preparation, review, or approval of the manuscript; and decision to submit the manuscript for publication.

Analyses were conducted in R version 4.0.4. P values were two-sided. MR results were visualised using the metafor or ggforestplot packages.^39^^,^^47^ The scripts used for the analyses can be found in our github repository (https://github.com/mightyphil2000/fatty-acids).

## Results

**Fig. 1:**
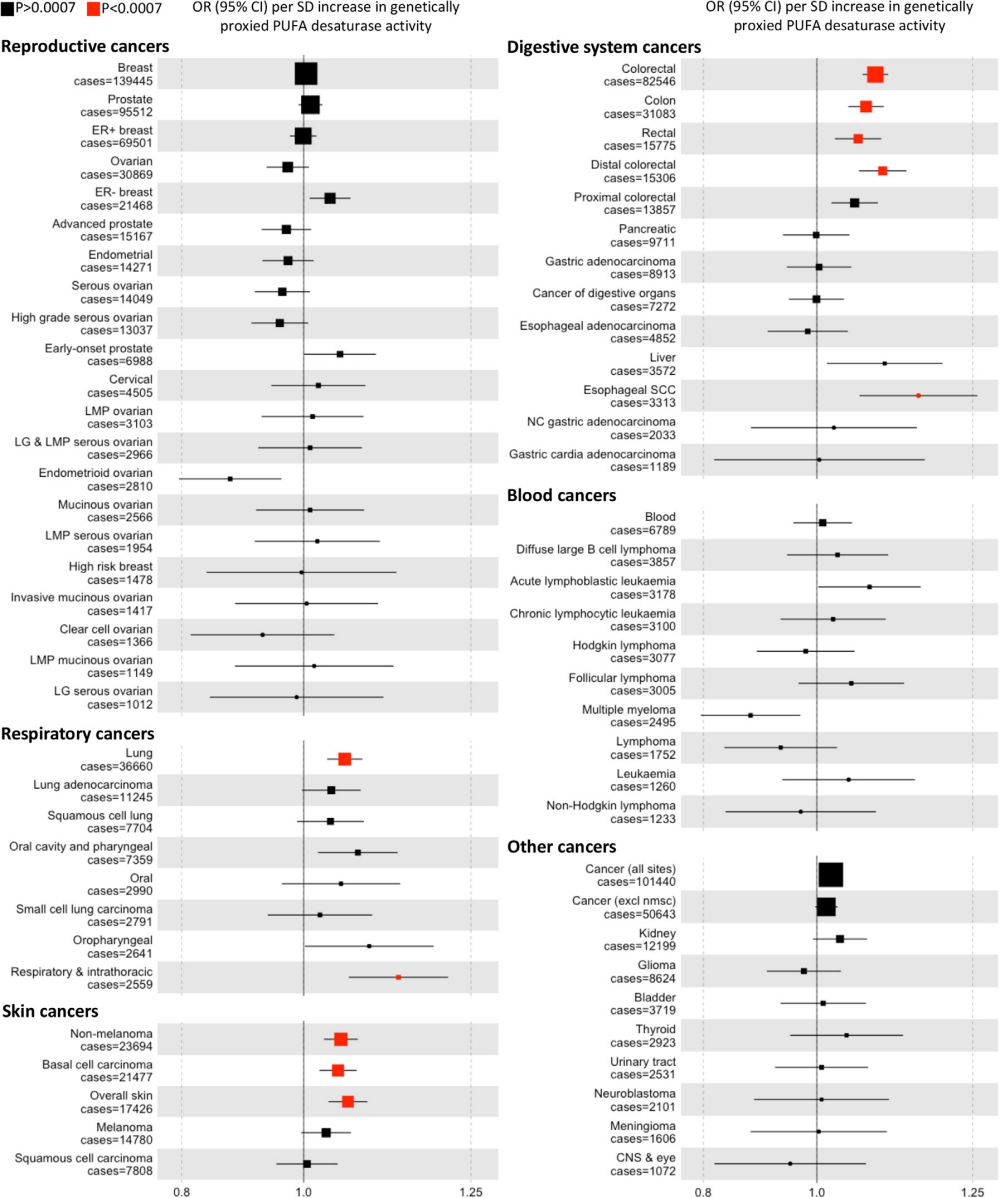
**Association between genetically proxied polyunsaturated fatty acid desaturase activity and risk of cancer**. Plotted data indicate odds ratios for cancer per standard deviation increase in polyunsaturated fatty acid desaturase activity instrumented by rs174546. Point sizes are proportional to the inverse of the variance for the log odds ratio. An alpha threshold of 0.0007 (0.05/67 cancers) was used to identify associations. Abbreviations: OR, odds ratio; SD, standard deviation; CI, confidence interval; nmsc, non-melanoma skin cancer, ER, estrogen receptor; LMP, low malignant potential; LG, low grade; SCC, squamous cell carcinoma; PUFA, polyunsaturated fatty acid.

Genetically proxied higher PUFA desaturase activity was associated (P < 0.0007) with higher risk of colorectal cancer (including subtypes), esophageal squamous cell carcinoma, lung cancer, respiratory and intrathoracic cancer, non-melanoma skin cancer, basal cell carcinoma, and overall skin cancer (Fig. 1 and Supplementary Table S7). Findings were not substantially different amongst independent studies for each cancer (P_het_ ≥ 0.03) (Supplementary Figs. S3–S8). Findings for colorectal cancer and lung cancer were similar amongst studies of European and East Asian ancestry (Supplementary Figs. S3 and S4) and were also similar amongst tumour subtypes for colorectal cancer (P = 0.13 for distal versus proximal colorectal cancer; P = 0.64 for colon versus rectal cancer) and lung cancer (P = 0.94 for difference amongst adenocarcinoma, squamous carcinoma and small cell lung cancer). We saw little evidence (OR reported per SD increase in PUFA desaturase activity) for associations with male and female reproductive cancers (OR = 1.00 [95% CI: 0.99–1.01],P_heterogeneity_ = 0.25), urinary system cancers (1.03 [0.98–1.07], P_heterogeneity_ = 0.51), nervous system cancers (0.99 [0.95–1.03], P_heterogeneity_ = 0.92) or blood cancers (1.01 [0.98–1.04], P_heterogeneity_ = 0.09) (Fig. 1 and Supplementary Table S8). A search of the GWAS catalog identified an association with increased risk of laryngeal squamous cell carcinoma in East Asians (OR = 1.37 [95% CI: 1.28–1.47] per copy of the allele associated with higher PUFA desaturase activity) (Supplementary Table S9).

### Secondary MR analyses

**Fig. 2:**
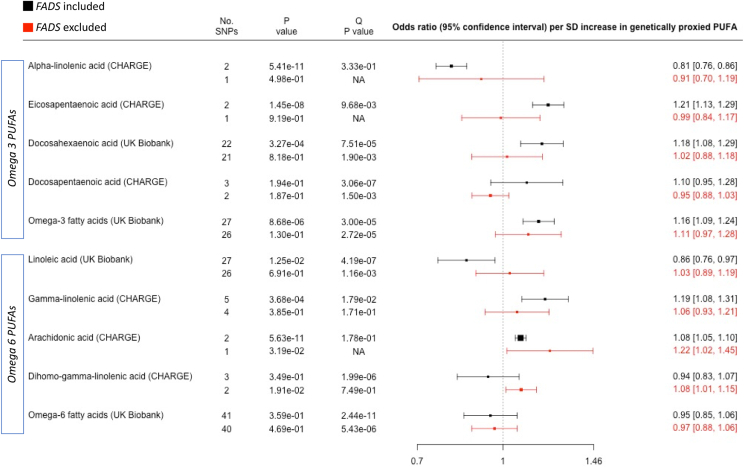
**Association between genetically elevated polyunsaturated fatty acids and risk of colorectal cancer in 58,131 cases and 67,347 controls**. Outcome summary data for colorectal cancer were derived from a meta-analysis of the GECCO, CORECT and CCFR studies. Summary data for fatty acid exposures were derived from either the CHARGE consortium or UK Biobank. The Q P value was derived from a Cochran's Q test for heterogeneity in MR results amongst SNPs in the genetic instrument. The P value column represents the P value for association between the PUFA and cancer, derived from inverse-variance weighted linear regression (>1 SNP) or a Wald ratio test (1 SNP). The “No. SNPs” column represents the number of SNPs present in the genetic instrument. The FADS region (proxied by rs174546) was either included (black data points) or excluded (red data points) from the genetic instrument. Individual PUFAs within the omega 3 and omega 6 sections are sorted according to chain length (shorter to longer). Abbreviations: PUFAs, polyunsaturated fatty acids; SD, standard deviation.

**Fig. 3:**
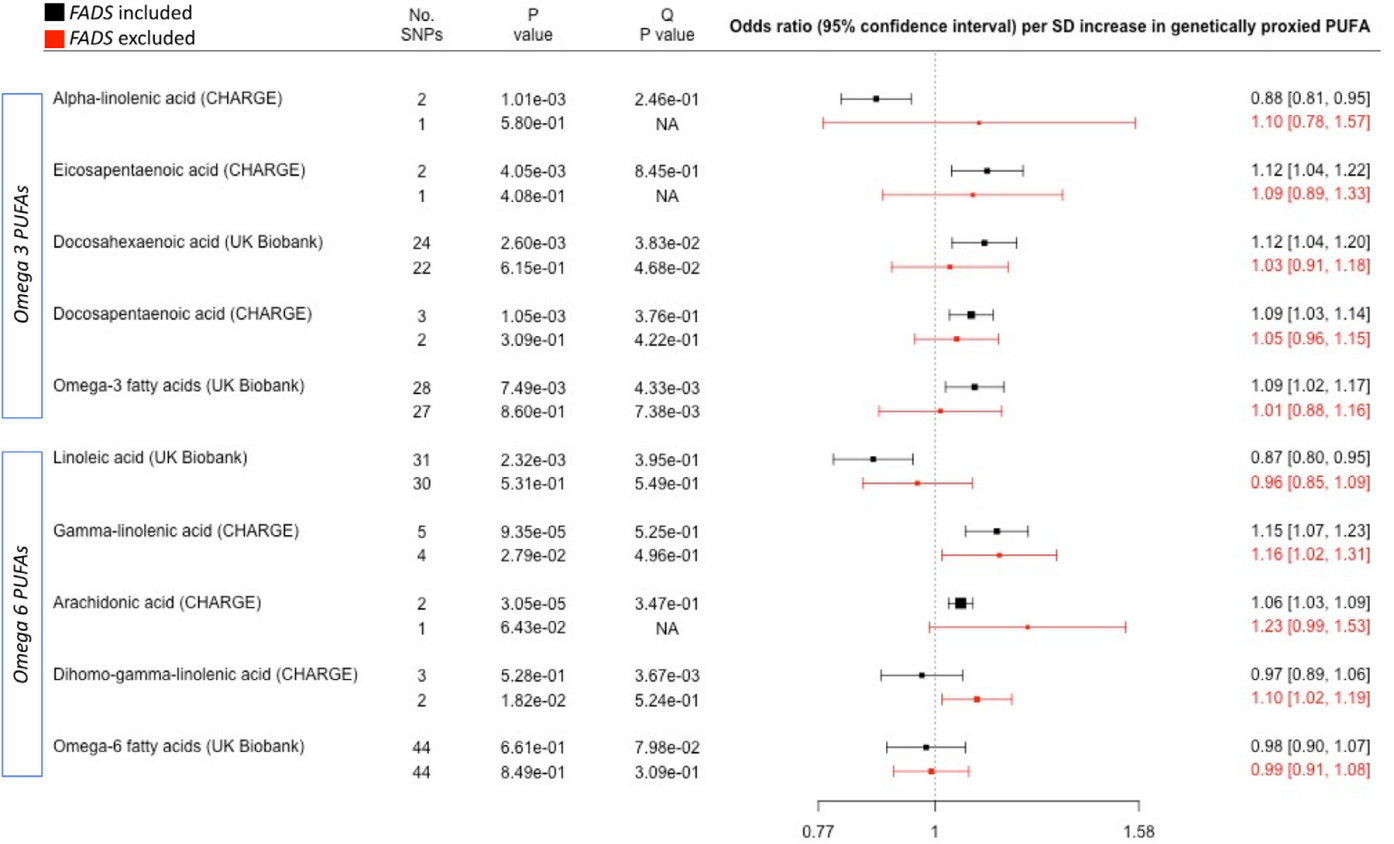
**Association between genetically elevated polyunsaturated fatty acids and risk of lung cancer in 31,937 cases and 428,466 controls**. Outcome summary data for lung cancer were derived from a meta-analysis of the ILCCO and UK Biobank. Summary data for fatty acid exposures were derived from either the CHARGE consortium or UK Biobank. The Q P value was derived from a Cochran's Q test for heterogeneity in MR results amongst SNPs in the genetic instrument. The P value column represents the P value for association between the PUFA and cancer, derived from inverse-variance weighted linear regression (>1 SNP) or a Wald ratio test (1 SNP). The “No. SNPs” column represents the number of SNPs present in the genetic instrument. The FADS region (proxied by rs174546) was either included (black data points) or excluded (red data points) from the genetic instrument. Individual PUFAs within the omega 3 and omega 6 sections are sorted according to chain length (shorter to longer). Abbreviations: PUFAs, polyunsaturated fatty acids; SD, standard deviation.

To assess the potential role of omega 3 and omega 6 PUFA exposures in our findings, we conducted additional MR analyses of colorectal cancer, lung cancer and basal cell skin cancer, restricted to individuals of European ancestry and excluding the *FADS* region from the genetic instrument (Fig. 2, Fig. 3 and Supplementary Fig. S9). Genetically proxied omega 3 PUFAs were not associated with the selected cancers in analyses that excluded the *FADS* region (P values > 0.13), albeit with wide confidence intervals indicating potential lack of power. In contrast, a number of omega 6 PUFAs were associated with risk of colorectal cancer and lung cancer (P < 0.05) in analyses that excluded the *FADS* region (OR [95% CI] per SD increase in genetically proxied PUFA): AA with higher risk of colorectal cancer (1.22 [1.02–1.45]), GLA with higher risk of lung cancer (1.16 [1.02–1.31]) and DGLA with higher risk of colorectal cancer (1.08 [1.01–1.15]) and lung cancer (1.10 [1.01–1.21]). No associations were observed with basal cell carcinoma.

### Sensitivity analyses

As a sensitivity analysis for genomic confounding, we assessed evidence for colocalisation of selected cancers with PUFA desaturase activity and with expression of the *FADS1* and *FADS2* genes in various tissues (Supplementary Fig. S10, Supplementary Table S10 and Supplementary results). Overall, the evidence for colocalisation was strongest for colorectal cancer and esophageal squamous cell carcinoma (posterior probabilities for a shared causal variant [PP_H4_] > 80%) and was weakest for basal cell carcinoma (PP_H4_ < 8%). There was strong evidence for colocalisation of lung cancer with *FADS1* gene expression in adipose subcutaneous tissue (PP_H4_ = 96%) but not with *FADS1* gene expression in lung tissue or with PUFA desaturase activity (PP_H4_ < 70%).

Findings from the within-sibship MR analyses were broadly like the primary MR results for overall cancer but were unclear for other selected cancers due to small sample sizes (Supplementary Fig. S11).

In effect decomposition analyses (Supplementary results), we found that the instrument for PUFA desaturase activity (rs174546) was associated (P < 0.0014) with LDL cholesterol, total cholesterol, triglycerides, HDL cholesterol, height, platelet count, heart rate and age at menopause (Supplementary Fig. S12). However, the magnitude of these associations was too small to account for our colorectal cancer or lung cancer findings (P values ≤ 0.013 for total versus indirect effects) or implied implausibly large effects on colorectal cancer (Supplementary Table S11). We also found that genetically proxied lifetime smoking could not account for our colorectal cancer, lung cancer or basal carcinoma findings (P values ≤ 4.72 × 10^−03^ for total versus indirect effects) (Supplementary Table S12). Similar findings were observed in decomposition analyses of cigarettes smoked per day and lung cancer in ever smokers (Supplementary Table S13).

### Sources of heterogeneity

There was little evidence that MR results varied by cancer incidence, survival time, median age-at-diagnosis, or tissue-specific rates of stem cell division (P ≥ 0.56, Supplementary Figs. S13–S16; Supplementary Table S14). MR results tended to be stronger for 13 “smoking-related” cancers (P = 0.003), nine cancers with an accepted relationship to chronic inflammatory conditions (P = 0.004) and digestive system cancers (P = 0.019) (Supplementary Figs. S17–S19). Results were similar in sensitivity analyses, including analyses that adjusted for potential sample overlap (Supplementary Table S14).

### MR-PheWAS

Genetically proxied PUFA desaturase activity was associated with higher risk of large artery stroke, asthma, nasal polyps, hypothyroidism but with lower risk of inflammatory bowel disease and Crohn's disease in an MR-PheWAS (P < 0.0006, Supplementary Fig. S20). This suggests that a hypothetical intervention to lower PUFA desaturase activity, for purposes of cancer prevention, might increase risk of inflammatory bowel disease and Crohn's disease. We did not see strong evidence for associations with bleeding disorders.

## Discussion

We found that genetically proxied PUFA desaturase activity was associated with higher risk of colorectal cancer, esophageal squamous cell carcinoma, basal cell carcinoma, lung cancer and laryngeal squamous cell carcinoma. Extending similar findings in previous studies,^48^, ^49^, ^50^, ^51^, ^52^, ^53^ we used colocalization analysis to demonstrate that MR results for colorectal cancer and esophageal squamous cell carcinoma, but not lung cancer or basal cell carcinoma, are robust to genomic confounding. We also conducted sensitivity analyses for violations of assumptions, which suggested that our findings cannot be entirely explained by confounding by population stratification or by pleiotropy with selected biomedical factors, including smoking, anthropometrics and lipids. We also found evidence that interventions to inhibit the PUFA desaturase activity, for cancer prevention, would increase risk of inflammatory bowel disease and Crohn's disease.

### Potential mechanisms

Our findings are compatible with a causal effect of increased PUFA desaturase activity on risk of colorectal cancer and esophageal squamous cell carcinoma. A plausible candidate mechanism is increased synthesis of AA, which is the preferred substrate for cyclooxygenases (COX) in the generation of pro-inflammatory and carcinogenic eicosanoids, such as prostaglandin E2 (PGE-2)^54^, ^55^, ^56^, ^57^ (see supplementary discussion for consideration of other potential pathways). This would be compatible with our secondary MR analyses, which highlighted AA as a potential mediator. Consistent with a pro-inflammatory mechanism, our MR results tended to be stronger for cancers with known relationships to chronic inflammatory conditions and smoking (a pro-inflammatory factor^54^, ^55^, ^56^, ^57^, ^58^, ^59^), and genetically proxied PUFA desaturase activity was associated with chronic inflammatory conditions in MR-PheWAS.

The association with higher risk of colorectal cancer but lower risk of inflammatory bowel disease may reflect arachidonic acid and its role in inflammation, tissue injury and wound healing.^60^ For example, eicosanoids derived from arachidonic acid metabolism play a role in tissue repair, which may underlie the inverse association with inflammatory bowel disease (since damage to the intestinal lining promotes development of inflammatory bowel disease^61^) but are also pro-inflammatory, which might account for associations with increased cancer risk.^54^, ^55^, ^56^, ^57^ Increased synthesis of eicosanoids, derived from arachidonic acid, may lead to increased cellular proliferation, promoting wound healing and decreasing risk of inflammatory bowel disease but promoting carcinogenesis. The increased cancer risk might result from mutagenic mechanisms (e.g. greater probability for cancer causing mutations in dividing cells), or might reflect non-mutagenic mechanisms, e.g. activation of otherwise dormant cells that are carriers of cancer driver mutations.^62^ This would also help explain why the strongest MR findings in our study were at tissue sites directly exposed to the external environment, which we speculate reflects the greater susceptibility of these tissues to external damage and increased cell turnover during tissue repair. These considerations are compatible with the known effect of non-steroidal anti-inflammatory drugs (NSAIDs), such as aspirin, which inhibit the metabolism of arachidonic acid, decrease risk of colorectal cancer and increase risk of inflammatory bowel disease. The increased risk of inflammatory bowel disease in aspirin users may reflect inhibition of wound healing, leading to increased interaction between the gut microbiome and immune cells in the intestine lining.^63^^,^^64^

### Clinical relevance

Our findings highlight PUFA biosynthesis and omega 6 fatty acids as possible intervention targets for colorectal cancer prevention, which might be achievable through dietary or chemoprevention strategies. For example, NSAIDs, such as aspirin, inhibit COX mediated metabolism of AA, which may contribute to the efficacy of NSAIDs for cancer prevention.^65^, ^66^, ^67^, ^68^, ^69^, ^70^, ^71^, ^72^ As to dietary strategies, competitive inhibition of omega 6 PUFA biosynthesis can be achieved through increased consumption of foods or supplements rich in omega 3 PUFAs.^73^, ^74^, ^75^, ^76^, ^77^ In the seAFOod Polyp Prevention trial participants,^78^ eicosapentaenoic acid (EPA, an omega-3 PUFA) and aspirin reduced the number of colorectal adenomas, a precursor for colorectal cancer, in individuals undergoing colonoscopy screening for one year. A potential safety concern is that NSAIDs may increase risk of inflammatory bowel disease and bleeding disorders. Consistent with this, our MR-PheWAS identified increased risk of inflammatory bowel disease, but not bleeding, as a potential consequence of interventions to inhibit PUFA biosynthesis pathway for cancer prevention.

To improve the safety profile, interventions could be targeted to high-risk individuals (e.g. those with advanced adenomas on colonoscopy screening). Targeted interventions may also be possible through treatment stratification on *FADS* genotypes, with the expectation that carriers of the C allele (the allele associated with higher PUFA desaturase activity and increased cancer risk) would obtain most benefit from interventions. Compatible with this idea, the association between the allele for increased PUFA desaturase activity and colorectal cancer risk was weaker in self-reported aspirin users.^50^ In addition, in a randomized crossover study, dietary omega 3 PUFAs reduced circulating AA levels, but this effect was greatest in carriers of the *FADS* risk allele.^76^ Better-powered studies are required, however, to validate these potential gene–environment interactions.

Dietary guidelines typically recommend replacement of saturated with polyunsaturated fat for prevention of coronary heart disease.^1^ Our findings suggest, however, that such advice should potentially be reconsidered in individuals with an increased risk of colorectal cancer.

We found little evidence for associations with most cancers, including common reproductive cancers (breast and prostate), which might help explain lack of observed benefit in RCTs in which overall cancer incidence was the primary endpoint.^16^^,^^17^

### Confounding by population structure

There is evidence that the *FADS* locus is under selection pressure,^79^ which increases the potential for confounding by population stratification. Arguing against this possibility: (1) findings from a within-sibling design were similar to primary MR results for overall cancer (other cancers could not be reliably assessed due to low power); (2) findings for AA were similar across analyses that excluded or included the *FADS* region; and (3) MR results for colorectal cancer and lung cancer were consistent across populations of European and East Asian ancestry.

### Strengths and limitations

Our study was large and well powered for multiple cancers and included East Asian and European ancestry studies. This allowed us to conduct a more systematic analysis and develop insights into potential sources of heterogeneity. Our MR results may, however, be susceptible to genomic confounding. Although this is less likely for cancers showing strong evidence for colocalisation, the presence of multiple causal variants within the same *FADS* haplotype^31^ would make it harder to identify evidence against colocalisation. Horizontal pleiotropy could also introduce alternative pathways from the genetic instrument to cancer, which would invalidate our conclusions. However, to account for our results, horizontal pleiotropy would have to operate prior to translation of *FADS* transcripts into PUFA desaturases. Given that our instrument directly affects PUFA desaturase activity–most likely via an effect on FADS gene expression (supported by our colocalisation results)–pleiotropy is more likely to be vertical, rather than horizontal, which would be compatible with MR assumptions. Further arguing against horizontal pleiotropy bias, in effect decomposition analyses we found that pleiotropy with 36 biomedical characteristics, including known cancer risk factors, could not account for our primary results. Results for individual PUFAs in secondary MR analyses will however be more susceptible to bias from horizontal pleiotropy. For example, if linoleic acid were to have a direct effect on cancer (i.e. an effect that was not mediated by other PUFAs) this would invalidate causal inferences about arachidonic acid. We did not correct findings from secondary analyses of individual PUFAs for multiple testing, which increases the potential for false positives. The latter results should however be considered exploratory and require confirmation in followup studies. Finally, our study focused on European and East Asian ancestry studies and therefore our findings may not be generalizable to other populations.

### Conclusion

The PUFA biosynthesis pathway may be an intervention target for prevention of colorectal cancer and esophageal squamous cell carcinoma but with potential for increased risk of inflammatory bowel disease.

## Contributors

All authors read and approved the final version of the manuscript. PCH and MCB verified the underlying data.

*Data curation and access*: Haycock, Borges, Cerhan, Lemaitre, OMara, Birmann, Spurdle, Iles, Law, Slager, Hosnijeh, Mariosa, Cotterchio, Peters, Enroth, Gharahkhani, Marchand, Block, Amos, Hung, Zheng, Gunter.

*Data interpretation*: Haycock, Borges, Cerhan, Burrows, Lemaitre, Burgess, Khankari, Tsilidis, Gaunt, Hemani, Zheng, Tuong, Birmann, OMara, Spurdle, Iles, Law, Slager, Hosnijeh, Mariosa, Cotterchio, Peters, Enroth, Gharahkhani, Marchand, Williams, Block, Amos, Hung, Zheng, Gunter, Davey Smith, Relton, Martin.

*Writing – review & editing*: Haycock, Borges, Burrows, Cerhan, Lemaitre, Burgess, Khankari, Tsilidis, Gaunt, Hemani, Zheng, Tuong, Birmann, OMara, Spurdle, Iles, Law, Slager, Hosnijeh, Mariosa, Cotterchio, Peters, Enroth, Gharahkhani, Marchand, Williams, Block, Amos, Hung, Zheng, Gunter, Davey Smith, Relton, Martin.

## Fatty Acids in Cancer Mendelian Randomization Collaboration

Nathan Tintle, Ulrike Peters, Terri Rice, Iona Cheng, Mark Jenkins, Steve Gallinger, Alex J. Cornish, Amit Sud, Jayaram Vijayakrishnan, Margaret Wrensch, Mattias Johansson, Aaron D. Norman, Alison Klein, Alyssa Clay-Gilmour, Andre Franke, Andres V. Ardisson Korat, Bill Wheeler, Björn Nilsson, Caren Smith, Chew-Kiat Heng, Ci Song, David Riadi, Elizabeth B. Claus, Eva Ellinghaus, Evgenia Ostroumova, Florent de Vathaire, Giovanni Cugliari, Giuseppe Matullo, Irene Oi-Lin Ng, James R. Cerhan, Jeanette E. Passow, Jia Nee Foo, Jiali Han, Jianjun Liu, Jill Barnholtz-Sloan, Joellen M. Schildkraut, John Maris, Joseph L. Wiemels, Kari Hemminki, Keming Yang, Lambertus A Kiemeney, Lang Wu, Laufey T Amundadottir, Marc-Henri Stern, Marie-Christine Boutron, Mark Martin Iles, Mark P. Purdue, Martin Stanulla, Melissa Bondy, Mia Gaudet, Lenha Mobuchon, Nicki J. Camp, Pak Chung Sham, Pascal Guénel, Paul Brennan, Philip R. Taylor, Puya Gharahkhani, Quinn Ostrom, Rachael Stolzenberg-Solomon, Rajkumar Dorajoo, Richard Houlston, Robert B Jenkins, Sharon Diskin, Sonja I. Berndt, Spiridon Tsavachidis, Stefan Enroth, Stephen J. Chanock, Tabitha Harrison, Tessel Galesloot, Ulf Gyllensten, Vijai Joseph, Yongyong Shi, Wenjian Yang, Yi Lin, Stephen K. Van Den Eeden, Guangfu Jin, Maria Carolina Borges, Kimberley Burrows, Rozenn N. Lemaitre, Sean Harrison, Stephen Burgess, Xuling Chang, Jason Westra, Nikhil K. Khankari, Kostas Tsilidis, Tom Gaunt, Gibran Hemani, Jie Zheng, Therese Truong, Tracy O’Mara, Amanda B. Spurdle, Matthew H. Law, Susan L. Slager, Brenda M. Birmann, Fatemeh Saberi Hosnijeh, Daniela Mariosa, Christopher I. Amos, Rayjean J. Hung, Wei Zheng, Marc J. Gunter, George Davey Smith, Caroline Relton, Richard M Martin, Philip C. Haycock.

## Data sharing statement

Details on how to access the summary data used in these analyses can be found in Haycock et al.^25^

## Declaration of interests

TRG has received funding from the Medical Research Council, Cancer Research UK and Biogen. BMB has received funding from the US National Institutes of Health/National Cancer Institute, American Institute of Cancer Research and Harvard Chan-NIEHS Center. JRC has received funding from the National Cancer Institute. GDS has received funding from the Medical Research Council. PG has received funding from the National Health and Medical Research Council. RM and PCH have received funding from Cancer Research UK. SB has received funding from the Wellcome Trust and the Medical Research Council. GDS reports Scientific Advisory Board Membership for Relation Therapeutics and Insitro.

## Appendix A Supplementary data

Supplementary_figures_resubmission Figs. S1–S21

Supplementary_materials

Supplementary_materials_track_changes

Supplemetary_tables_resubmission Tables S1–S14
