# Supplementary material for: The association between genetically elevated polyunsaturated fatty acids and risk of cancer
Source: eBioMedicine. 2023 Apr 20;91:104510. doi: 10.1016/j.ebiom.2023.104510 (PMC10148095; doi:10.1016/j.ebiom.2023.104510)
Supplement: Supplementary_figures_resubmission Figs. S1–S21 [file mmc1.docx]

Supplementary figure S1. Study design flow chart

Supplementary figure S2. Polyunsaturated fatty acid biosynthesis pathway

*FADS1* codes for delta-5 (D5D) desaturase and *FADS2* codes for delta-6 (D6D) desaturase.

Supplementary figure S3. Genetically proxied polyunsaturated fatty acid desaturase activity and risk of colorectal cancer in separate studies of the FAMRC

OR (95% CI) per SD increase in

genetically proxied PUFA desaturase activity


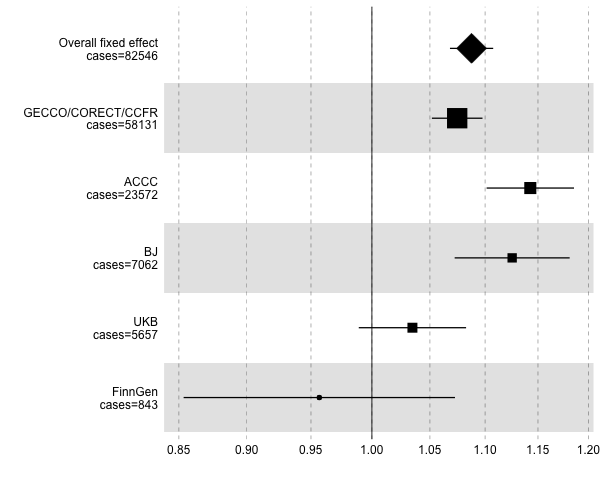


East Asian ancestry studies = ACCC and BJ; European ancestry studies = GECCO/CORECT/CCFR, UKB, FinnGen. The overall-fixed effect was estimated by inverse-variance weighted fixed effects meta-analysis of the GECCO/CORECT/CCFR, ACCC and FinnGen studies (BJ and UKB were excluded from the meta-analysis because they were previously included in the ACCC and GECCO/CORECT/CCFR GWAS analyses, respectively). Abbreviations: CI, confidence interval; OR, odds ratio; SD, standard deviation. FAMRC, Fatty Acids in Cancer Mendelian Randomization Collaboration. Study acronyms explained in supplementary table S6.

Supplementary figure S4. Genetically proxied polyunsaturated fatty acid desaturase activity and risk of lung cancer in independent studies of the FAMRC

OR (95% CI) per SD increase in

genetically proxied PUFA desaturase activity


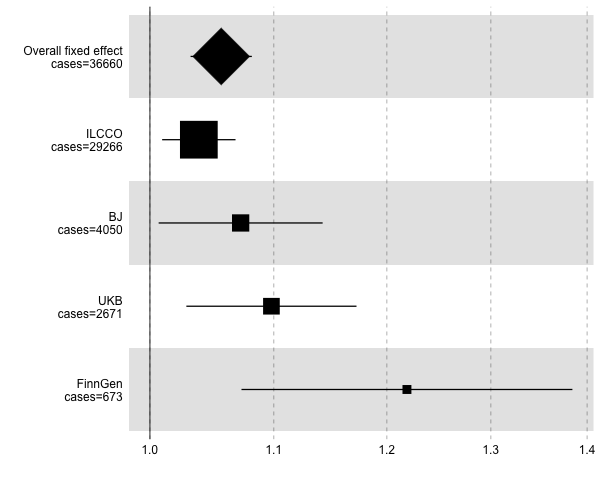


East Asian ancestry studies = BJ; European ancestry studies = ILCCO, UKB and FinnGen. Abbreviations: CI, confidence interval; OR, odds ratio; SD, standard deviation. FAMRC, Fatty Acids in Cancer Mendelian Randomization Collaboration. Study acronyms explained in supplementary table S6.

Supplementary figure S5. Genetically proxied polyunsaturated fatty acid desaturase activity and risk of squamous esophageal cancer in independent studies of the FAMRC


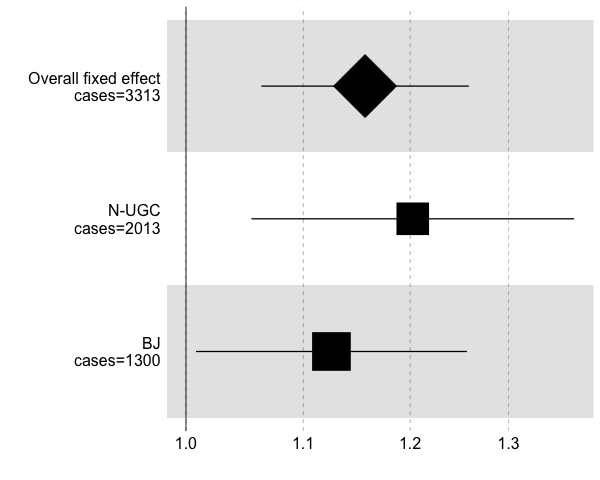


OR (95% CI) per SD change in genetically elevated FADS1/2 activity

OR (95% CI) per SD increase in

genetically proxied PUFA desaturase activity

Abbreviations: CI, confidence interval; FAMRC, Fatty Acids in Cancer Mendelian Randomization Collaboration; OR, odds ratio; SD, standard deviation. Study acronyms explained in supplementary table S6.

Supplementary figure S6. Genetically proxied polyunsaturated fatty acid desaturase activity and risk of overall skin cancer in independent studies of the FAMRC


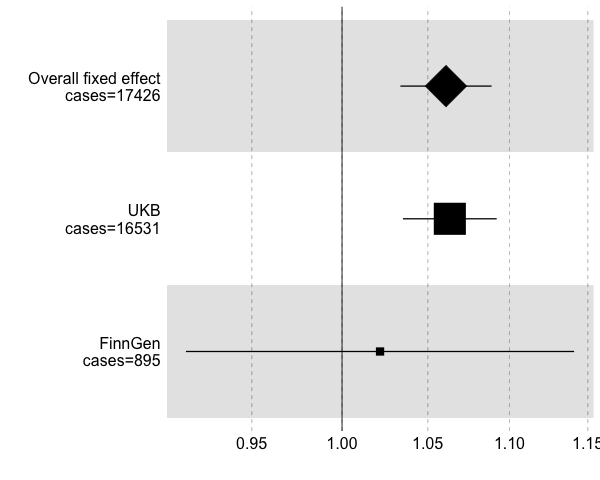


OR (95% CI) per SD change in genetically elevated FADS1/2 activity

OR (95% CI) per SD increase in

genetically proxied PUFA desaturase activity

Abbreviations: CI, confidence interval; FAMRC, Fatty Acids in Cancer Mendelian Randomization Collaboration; OR, odds ratio; SD, standard deviation. Study acronyms explained in supplementary table S6.

Supplementary figure S7. Genetically proxied polyunsaturated fatty acid desaturase activity and risk of basal cell carcinoma in independent studies of the FAMRC


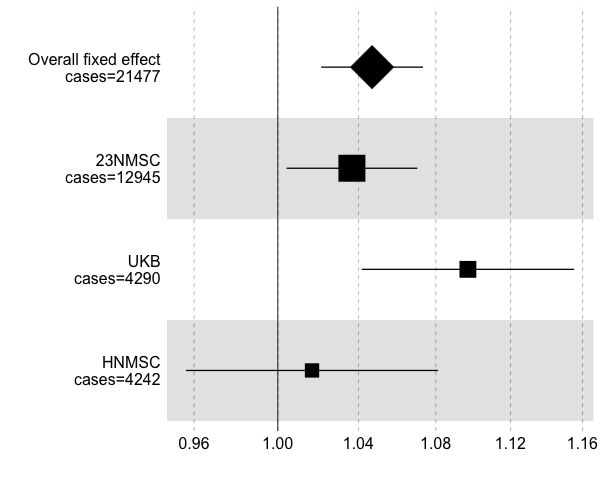


OR (95% CI) per SD change in genetically elevated FADS1/2 activity

OR (95% CI) per SD increase

genetically proxied PUFA desaturase activity

Abbreviations: CI, confidence interval; FAMRC, Fatty Acids in Cancer Mendelian Randomization Collaboration; OR, odds ratio; SD, standard deviation. Study acronyms explained in supplementary table S6.

Supplementary figure S8. Genetically proxied polyunsaturated fatty acid desaturase activity and risk of respiratory and intrathoracic cancer in independent studies of the FAMRC

OR (95% CI) per SD increase in

genetically proxied PUFA desaturase activity


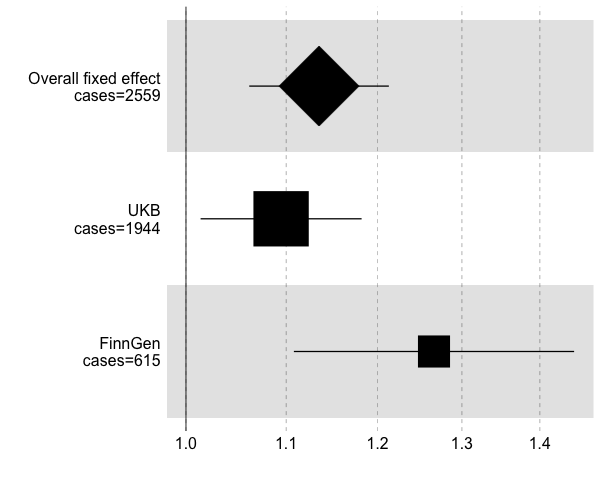


OR (95% CI) per SD change in genetically elevated FADS1/2 activity

Abbreviations: CI, confidence interval; FAMRC, Fatty Acids in Cancer Mendelian Randomization Collaboration; OR, odds ratio; SD, standard deviation. Study acronyms explained in supplementary table S6.

Supplementary figure S9. Association between genetically elevated polyunsaturated fatty acids and risk of basal cell carcinoma in up to 17,235 cases and 732,895 controls

Outcome summary data for basal cell carcinoma were derived from UK Biobank. Summary data for fatty acid exposures were derived from either the CHARGE consortium or UK Biobank. The Q p value was derived from a Cochran’s Q test for heterogeneity in MR results amongst SNPs in the genetic instrument. The P value column represents the P value for association between the PUFA and cancer, derived from inverse-variance weighted linear regression (>1 SNP) or a Wald ratio test (1 SNP). The “No. SNPs” column represents the number of SNPs present in the genetic instrument. The FADS region (proxied by rs174546) was either included (black data points) or excluded (red data points) from the genetic instrument. Individual PUFAs within the omega 3 and omega 6 sections are sorted according to chain length (shorter to longer). Abbreviations: PUFAs, polyunsaturated fatty acids; SD, standard deviation.

Supplementary figure S10. Colocalisation of PUFA desaturase activity with *FADS1* and *FADS2* gene expression and selected cancers ­­­­at 11q12.2 in Europeans and East Asians

The Y axis refers to the association P value (on a -log10 scale) for each SNP; the x axis corresponds to the base pair position for each SNP; each data point corresponds to a single SNP; Abbreviations: ILCCO, The International Lung Cancer Consortium; UKB, UK Biobank; BBJ or BJ, BioBank Japan study; GTEx, Genotype-Tissue Expression (GTEx) project (version 8); Cohorts for Heart and Aging Research in Genomic Epidemiology (CHARGE); GECCO/CORECT/CCFR, Genetics and Epidemiology of Colorectal Cancer Consortium/Colorectal Transdisciplinary study/Colon Cancer Family Registry; N-UGC, NCI Upper Gastrointestinal Cancer Study; ACCC, Asian Colorectal Cancer; SCHS, Singapore Chinese Health Study (SCHS) Consortium; AA, arachidonic acid (20:4n6); DGLA, dihomo-gamma-linolenic acid (20:3n6); LA, linoleic acid (18:2n6); PUFA desat., polyunsaturated fatty acid desaturase activity; FADS, fatty acid desaturase; r2, correlation between rs174546 and other SNPs

Supplementary figure S11. Within sibship MR analysis of the association between genetically elevated PUFA desaturase activity and risk of selected cancers

Cancer (3 sites) refers to combined analysis of colorectal, lung and overall skin cancer

Supplementary figure S12. Association between rs174546 and 36 selected biomedical characteristics

Sig=P<0.0013 [alpha of 0.05/36]; SD, standard deviation; CI, confidence interval. The major allele was allele C and is the allele associated with higher PUFA desaturase activity; PUFA, polyunsaturated fatty acid; LDL, low densitity lipoprotein; HDL, high densitity lipoprotein; AA:DGLA, ratio of arachidonic acid to dihomo-gamma-linolenic acid, which was used as a biomarker for PUFA desaturase activity in European ancestry studies.

Supplementary figure S13. The association between genetically elevated PUFA desaturase activity and odds of cancer as a function of the average lifetime number of stem cell divisions


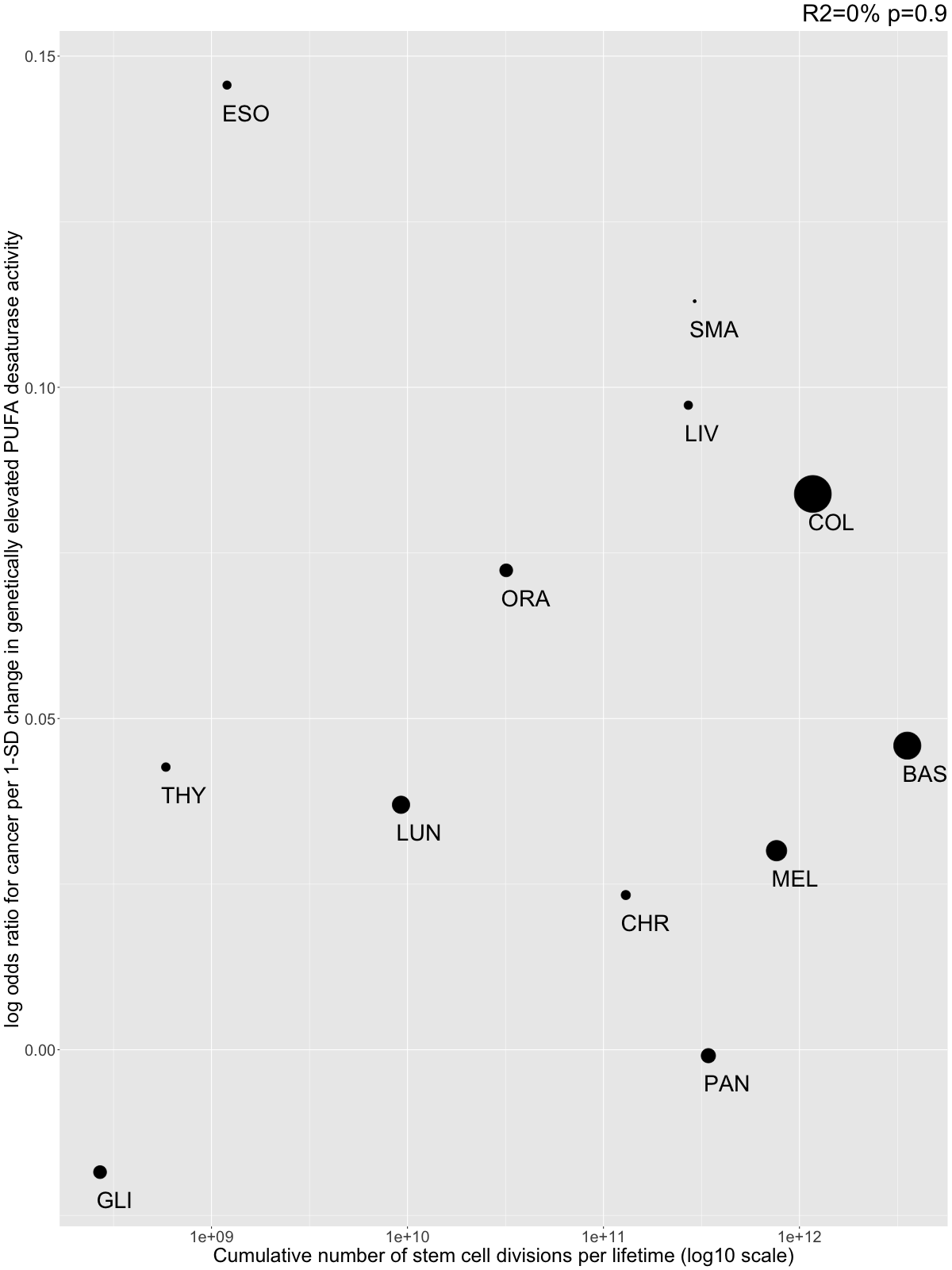


The plotted data show how the strength of the relationship between genetically increased PUFA desaturase activity and cancer varies by the average lifetime number of stem cell divisions. The R2 statistic indicates how much of the variation in MR results between cancers can be explained by average lifetime number of stem cell divisions. Circle sizes are proportional to the inverse of the variance of the log OR.

COL Colorectal cancer; PAN Pancreatic cancer; ORA Oral cavity and pharyngeal cancer; LIV Liver cancer; ESO Esophageal squamous cell carcinoma; BAS Basal cell carcinoma; MEL Melanoma; LUN Lung adenocarcinoma; GLI Glioma; CHR Chronic lymphocytic leukaemia; THY Thyroid cancer; SMA Small bowel cancer. Abbreviations: PUFA, polyunsaturated fatty acid; details of the cancers included in the analyses can be found in supplementary tables S4-S6 & S17; further details of the results can be found in supplementary table S14

Supplementary figure S14. The association between genetically elevated PUFA desaturase activity and odds of cancer as a function of cancer incidence in the Surveillance, Epidemiology, and End Results Program


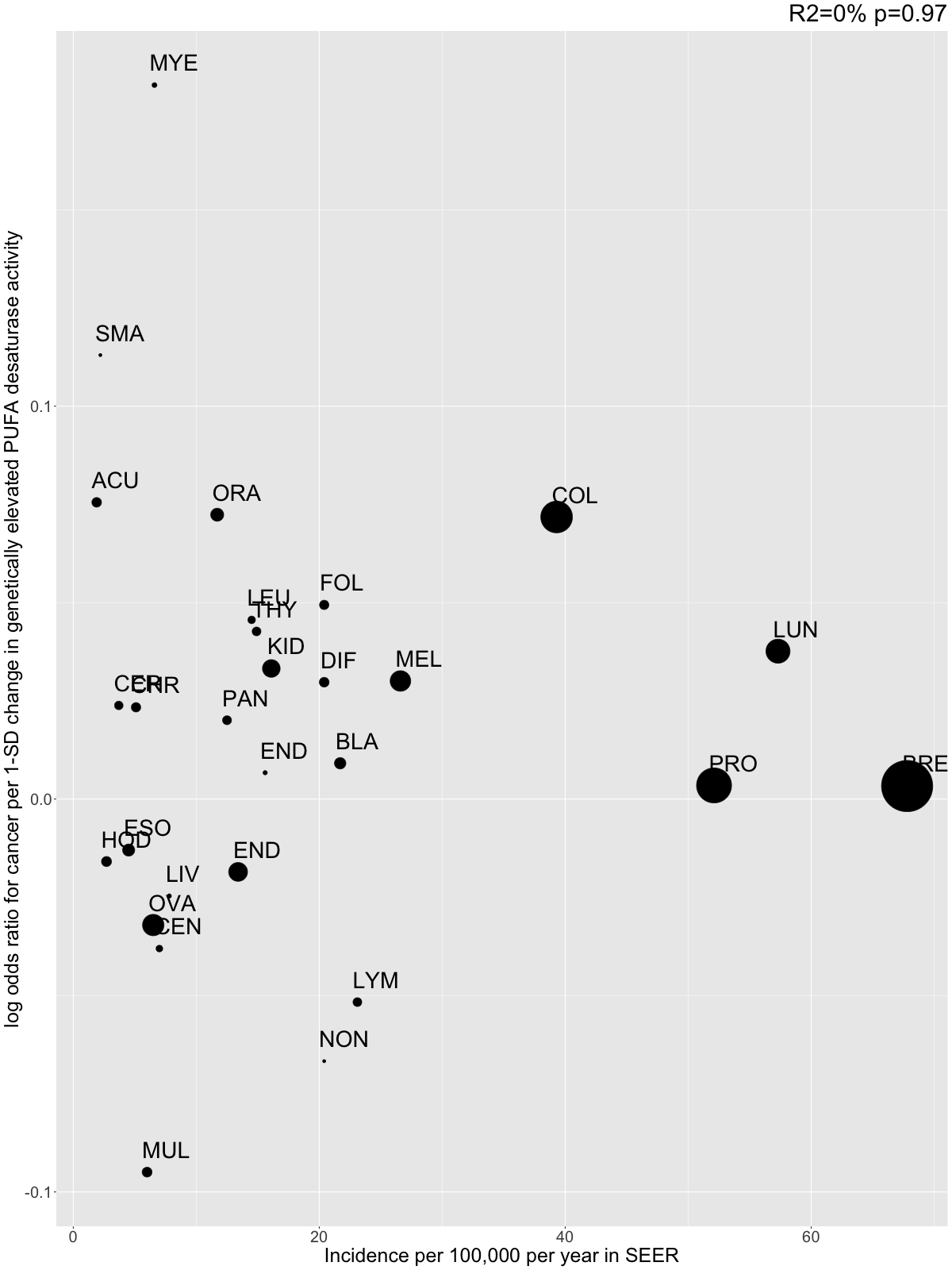


The plotted data show how the strength of the relationship between genetically increased PUFA desaturase activity and cancer varies by cancer incidence in the Surveillance, Epidemiology, and End Results Program. The R2 statistic indicates how much of the variation in MR results between cancers can be explained by cancer incidence. Circle sizes are proportional to the inverse of the variance of the log OR. COL Colorectal cancer; LUN Lung cancer; KID Kidney cancer; ORA Oral cavity and pharyngeal cancer; ESO Esophageal adenocarcinoma; PAN Pancreatic cáncer; BLA Bladder cancer; CER Cervical cancer; MYE Myeloid leukaemia; BRE Breast cancer; PRO Prostate cancer; OVA Ovarian cancer; MEL Melanoma; END Endometrial cancer; DIF Diffuse large b cell lymphoma; ACU Acute lymphoblastic leukaemia; CHR Chronic lymphocytic leukaemia; HOD Hodgkin’s lymphoma; FOL Follicular lymphoma; THY Thyroid cancer; MUL Multiple myeloma; LYM Lymphoma; LEU Leukaemia; CEN Central nervous system and eye cancer; LIV Liver & bile duct cancer; END Endocrine gland cancer; SMA Small bowel cancer; NON Non-hodgkin lymphoma unspecified. Abbreviations: PUFA, polyunsaturated fatty acid; details of the cancers included in the analyses can be found in supplementary tables S4-S6 & S17; further details of the results can be found in supplementary table S14

Supplementary figure S15. The association between genetically elevated PUFA desaturase activity and odds of cancer as a function of 5 year survival rate in the Surveillance, Epidemiology, and End Results Program


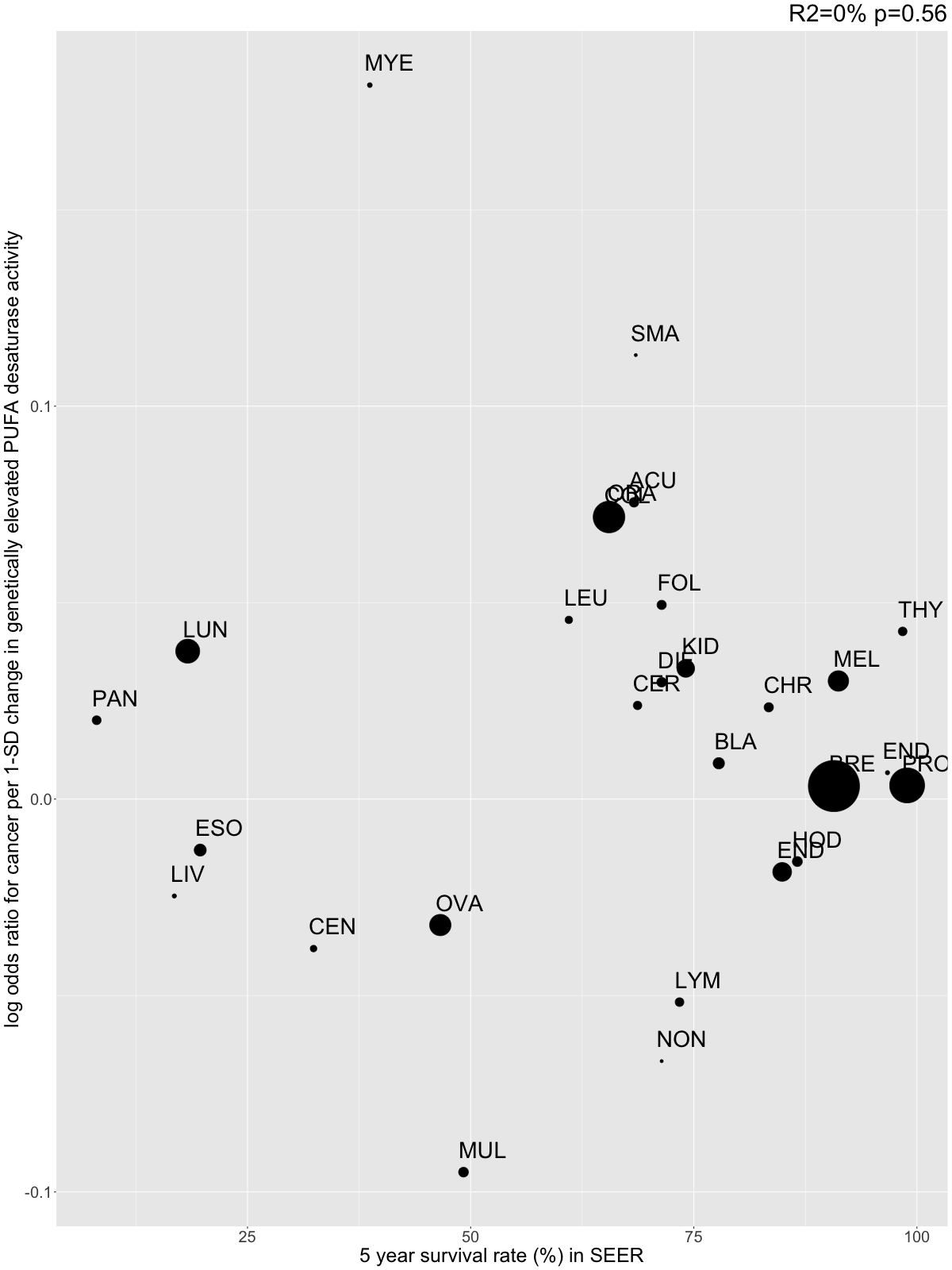


The plotted data show how the strength of the relationship between genetically increased PUFA desaturase activity and cancer varies by 5 year survival rates in the Surveillance, Epidemiology, and End Results Program. The *R^2^* statistic indicates how much of the variation in MR results between cancers can be explained by 5 year survival rates. Circle sizes are proportional to the inverse of the variance of the log OR. COL Colorectal cancer; LUN Lung cancer; KID Kidney cancer; ORA Oral cavity and pharyngeal cancer; ESO Esophageal adenocarcinoma; PAN Pancreatic cáncer; BLA Bladder cancer; CER Cervical cancer; MYE Myeloid leukaemia; BRE Breast cancer; PRO Prostate cancer

OVA Ovarian cancer; MEL Melanoma; END Endometrial cancer; DIF Diffuse large b cell lymphoma; ACU Acute lymphoblastic leukaemia; CHR Chronic lymphocytic leukaemia; HOD Hodgkin’s lymphoma; FOL Follicular lymphoma; THY Thyroid cancer; MUL Multiple myeloma; LYM Lymphoma; LEU Leukaemia; CEN Central nervous system and eye cancer; LIV Liver & bile duct cancer; END Endocrine gland cancer; SMA Small bowel cancer; NON Non-hodgkin lymphoma unspecified. Abbreviations: PUFA, polyunsaturated fatty acid; details of the cancers included in the analyses can be found in supplementary tables S4-S6 & S17; further details of the results can be found in supplementary table S14

Supplementary figure S16. The association between genetically elevated PUFA desaturase activity and odds of cancer as a function of median age at diagnosis in the Surveillance, Epidemiology, and End Results Program


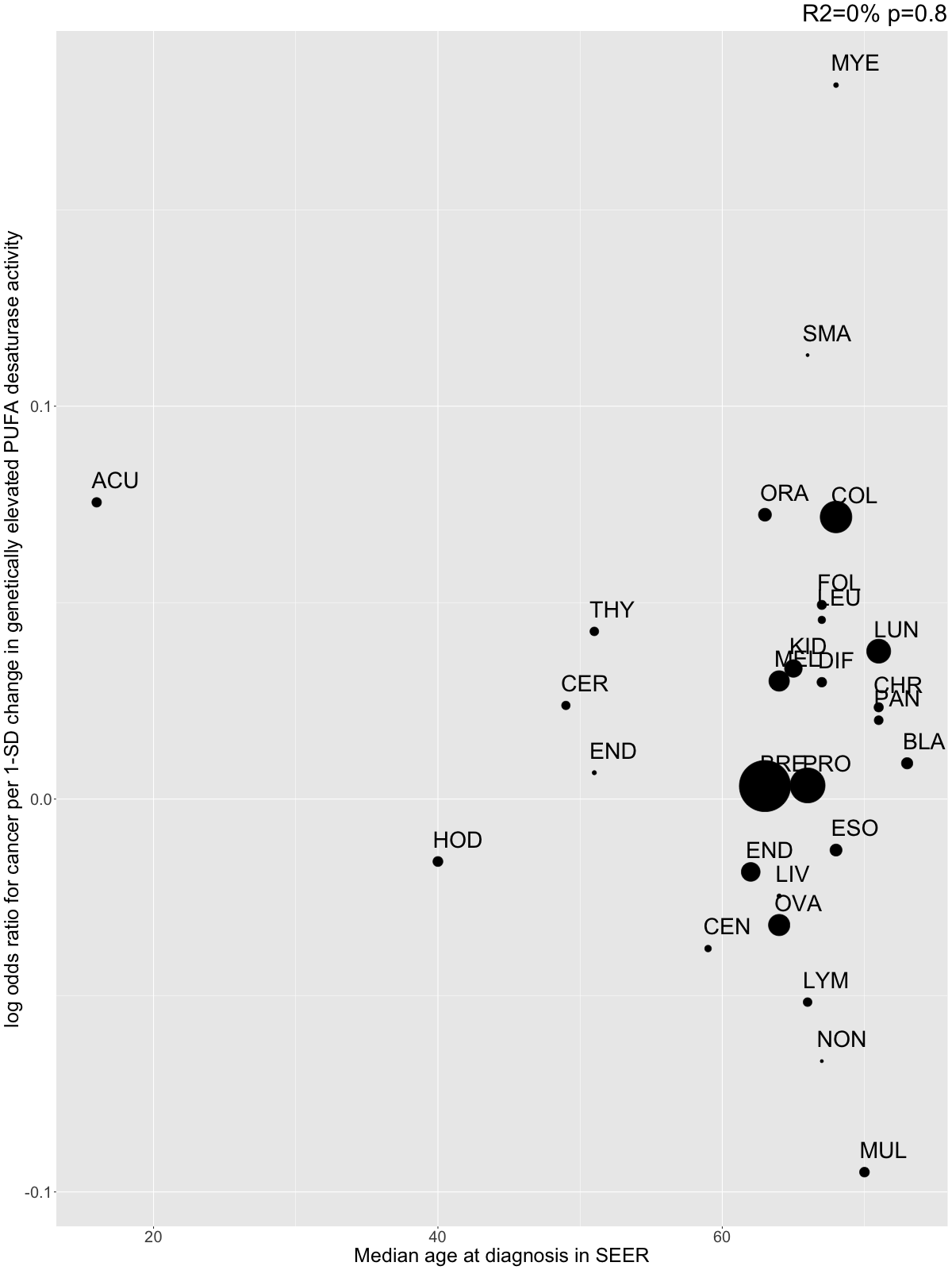


The plotted data show how the strength of the relationship between genetically increased PUFA desaturase activity and cancer varies by median age at diagnosis in the Surveillance, Epidemiology, and End Results Program. The R2 statistic indicates how much of the variation in MR results between cancers can be explained by median age at diagnosis. Circle sizes are proportional to the inverse of the variance of the log OR. COL Colorectal cancer; LUN Lung cancer; KID Kidney cancer; ORA Oral cavity and pharyngeal cancer; ESO Esophageal adenocarcinoma; PAN Pancreatic cáncer; BLA Bladder cancer; CER Cervical cancer; MYE Myeloid leukaemia; BRE Breast cancer; PRO Prostate cancer; OVA Ovarian cancer; MEL Melanoma; END Endometrial cancer; DIF Diffuse large b cell lymphoma; ACU Acute lymphoblastic leukaemia; CHR Chronic lymphocytic leukaemia; HOD Hodgkin’s lymphoma; FOL Follicular lymphoma; THY Thyroid cancer; MUL Multiple myeloma; LYM Lymphoma; LEU Leukaemia; CEN Central nervous system and eye cancer; LIV Liver & bile duct cancer; END Endocrine gland cancer; SMA Small bowel cancer; NON Non-hodgkin lymphoma unspecified. Abbreviations: PUFA, polyunsaturated fatty acid; details of the cancers included in the analyses can be found in supplementary tables S4-S6 & S17; further details of the results can be found in supplementary table S14

Supplementary figure S17. Association of genetically elevated polyunsaturated fatty acid desaturase activity with risk of smoking-related and other cancers

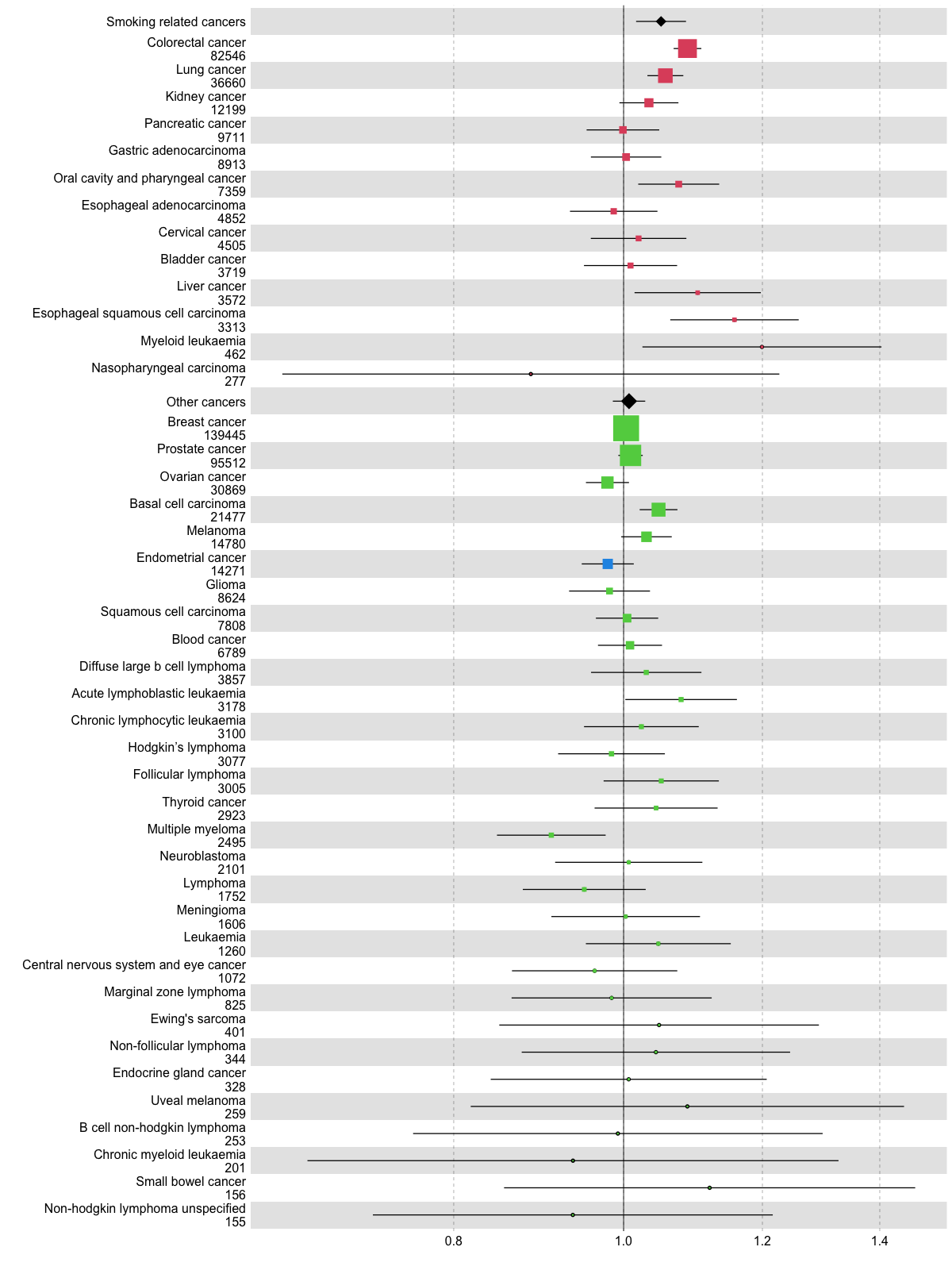
­

OR (95% CI) per SD increase

in genetically proxied PUFA desaturase activity

Abbreviations: OR, odds ratio; CI, confidence interval; SD, standard deviation; PUFA, polyunsaturated fatty acid; details of the cancers included in the analyses can be found in supplementary tables S4-S6 & S17; further details of the results can be found in supplementary table S14

Supplementary figure S18. Association between genetically proxied polyunsaturated fatty acid desaturase activity and cancer risk grouped according to the cancer’s relationship to chronic inflammatory conditions

Accepted relationship with a chronic inflammatory condition

Unknown/uncertain association with chronic inflammatory condition


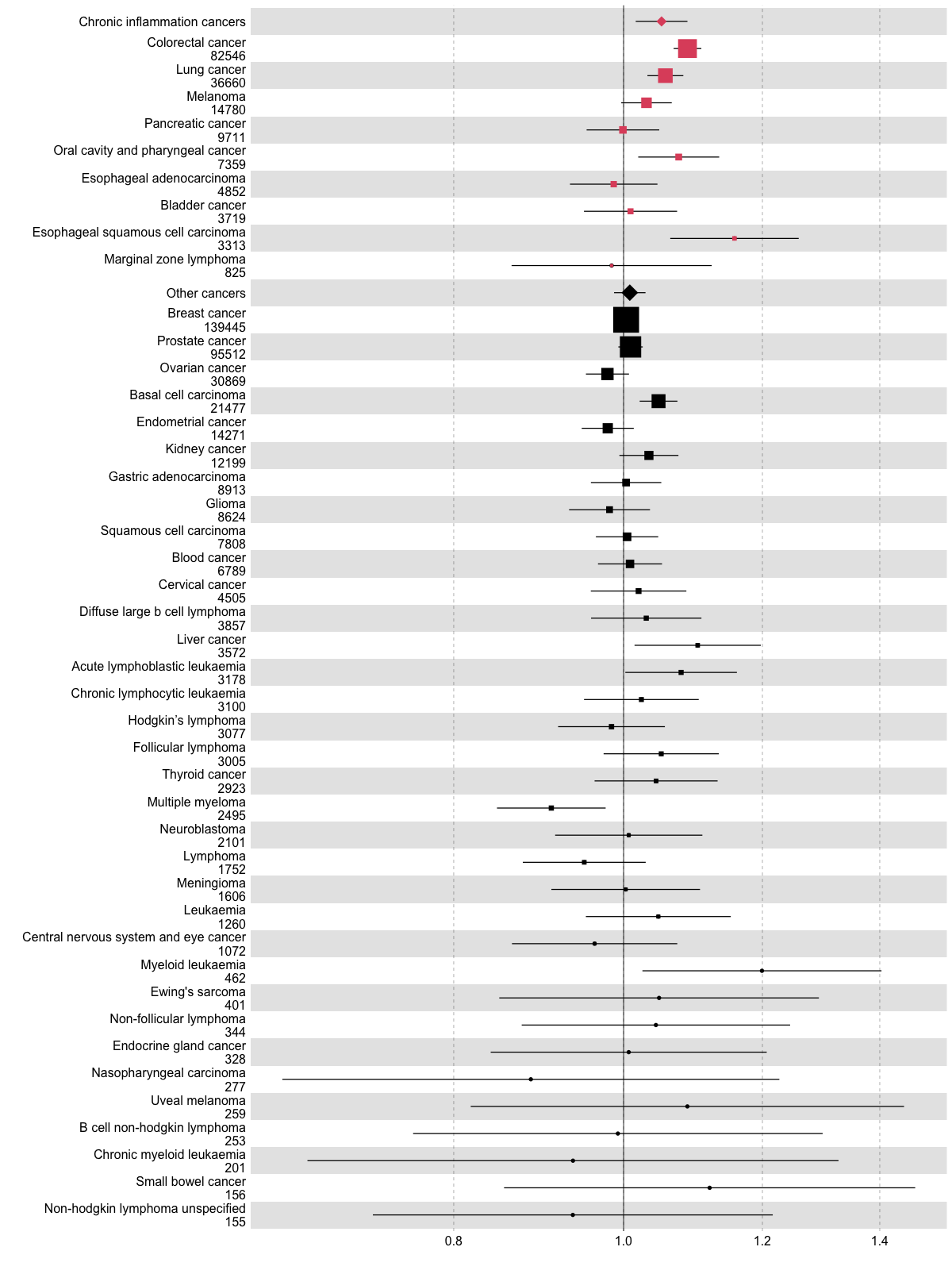


OR (95% CI) per SD increase

in genetically proxied PUFA desaturase activity

Abbreviations: OR, odds ratio; CI, confidence interval; SD, standard deviation; PUFA, polyunsaturated fatty acid; details of the cancers included in the analyses can be found in supplementary tables S4-S6 & S17; further details of the results can be found in supplementary table S14

Supplementary figure S19. Association of genetically proxied polyunsaturated fatty acid desaturase activity with digestive and non-digestive system cancers

Cancers of the digestive system

Non digestive system cancers


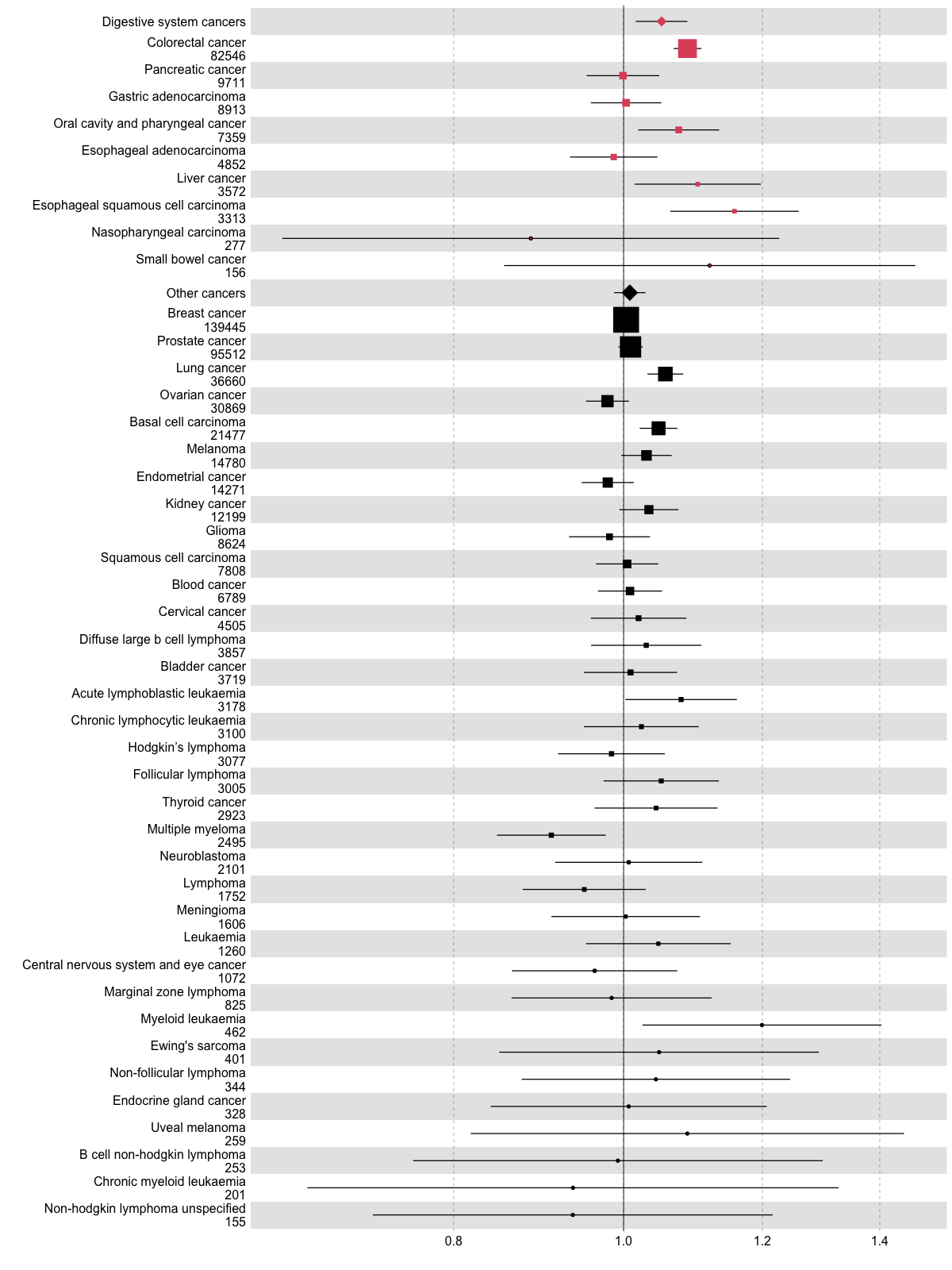


OR (95% CI) per SD increase

in genetically proxied PUFA desaturase activity

Abbreviations: OR, odds ratio; CI, confidence interval; SD, standard deviation; PUFA, polyunsaturated fatty acid; details of the cancers included in the analyses can be found in supplementary tables S4-S6 & S17; further details of the results can be found in supplementary table S14

Supplementary figure S20. Association between genetically proxied polyunsaturated fatty acid desaturase activity and risk of non-neoplastic diseases

P_Bonf._<0.05

P_Bonf._>0.05

Supplementary figure S21. Association between genetically elevated polyunsaturated fatty acids and risk of lung cancer using alternative strategies to combined results across studies

Outcome summary data for lung cancer were derived from a meta-analysis of ILCCO and UK Biobank. The meta-analysis was conducted using two strategies. In the first approach (MR-by-study), we ran Mendelian randomisation (MR) analyses separately for ILCCO and UK Biobank and then combined the MR results by fixed effects meta-analysis. In an alternative second approach (MR-pooled-study), we first combined the summary genetic data across ILCCO and UK Biobank by fixed effects meta-analysis and then ran the MR analysis on the resulting pooled dataset. The FADS region (proxied by rs174546) was either included (red data points) or excluded (black data points) from the genetic instrument. Abbreviations: CI, confidence interval; ILCCO, International Lung Cancer Consortium; PUFAs, polyunsaturated fatty acids; SD, standard deviation.
