# Supplementary material for: The association between genetically elevated polyunsaturated fatty acids and risk of cancer
Source: eBioMedicine. 2023 Apr 20;91:104510. doi: 10.1016/j.ebiom.2023.104510 (PMC10148095; doi:10.1016/j.ebiom.2023.104510)
Supplement: Supplementary_materials [file mmc2.docx]

[Supplementary methods 2](#_Toc131671202)

[Literature search 2](#_Toc131671203)

[Potential biases of the instrument selection strategy for secondary PUFA exposures 2](#_Toc131671204)

[Clumping procedure to identify independent genetic associations 3](#_Toc131671205)

[Instrument for PUFA desaturase activity 3](#_Toc131671206)

[Defining genetic instruments for secondary exposures 3](#_Toc131671207)

[Combining results across studies 4](#_Toc131671208)

[Making allowance for sample overlap between studies 4](#_Toc131671209)

[Sensitivity analyses for violations of Mendelian randomization analytical assumptions 5](#_Toc131671210)

[Colocalisation analysis 5](#_Toc131671211)

[Within-sibship MR analyses 5](#_Toc131671212)

[Effect decomposition analyses 6](#_Toc131671213)

[Modelling sources of heterogeneity in MR findings amongst cancer sites 6](#_Toc131671214)

[Note on how number of cases and controls was estimated 8](#_Toc131671215)

[Note on esophageal squamous cell carcinoma 8](#_Toc131671216)

[Supplementary results 8](#_Toc131671217)

[Note on results for skin cancers 8](#_Toc131671218)

[Error in the MR analysis of non-Hodgkin lymphoma 8](#_Toc131671219)

[Sensitivity analyses for violations of Mendelian randomization analytical assumptions 9](#_Toc131671220)

[Colocalisation 9](#_Toc131671221)

[Effect decomposition analyses 9](#_Toc131671222)

[Supplementary discussion 10](#_Toc131671223)

[Proxying multiple independent points in the PUFA biosynthesis pathway 11](#_Toc131671224)

[Role of FADS activity 11](#_Toc131671225)

[Clinical implications of global variation in rs174546 C allele prevalence 11](#_Toc131671226)

[Study acknowledgements 12](#_Toc131671227)

[23andMe 12](#_Toc131671228)

[Asian Colorectal Cancer Consortium (ACCC) 12](#_Toc131671229)

[Endometrial Cancer Association Consortium (ECAC) 12](#_Toc131671230)

[The EPIdemiology of THYRoid cancer consortium (EPITHYR) 13](#_Toc131671231)

[Esophageal Adenocarcinoma Study (EAS) 13](#_Toc131671232)

[Colon Cancer Family Registry-Colorectal Transdisciplinary study- Genetics and Epidemiology of Colorectal Cancer Consortium (CCFR-CORECT-GECCO) 13](#_Toc131671233)

[InterLymph 14](#_Toc131671234)

[International Lung Cancer Consortium (ILCCO) 15](#_Toc131671235)

[Melanoma Meta-analysis Consortium (MMAC) 16](#_Toc131671236)

[Pancreatic Cancer Case-Control Consortium (PanC4) 21](#_Toc131671237)

[The Prostate Cancer Association Group to Investigate Cancer Associated Alterations in the Genome (PRACTICAL) Consortium 22](#_Toc131671238)

[References 26](#_Toc131671239)

# Supplementary methods

## Literature search

We searched for meta-analyses of observational studies of the association between polyunsaturated fatty acids (PUFAs) and cancer risk using the following search terms: ("meta-analysis" OR "meta analysis" OR "systematic review") AND (omega OR polyunsaturated) AND cancer. We also searched for randomized controlled trials (RCTs) of PUFAs and cancer risk using: ("randomized controlled trial" OR "randomised controlled trial") AND (omega or polyunsaturated) AND cancer. We searched PubMed up until 10 April 2022 with no language restrictions.

## Potential biases of the instrument selection strategy for secondary PUFA exposures

We investigated individual PUFA exposures in secondary analyses of the cancers that were associated with PUFA desaturase activity in the primary analyses. The investigated PUFA exposures were: alpha-linolenic acid (ALA;18:3n3), docosahexaenoic acid (DHA; 22:6n3), docosapentaenoic acid (DPA; 22:5n3), eicosapentaenoic acid (EPA, 20:5n3), total omega-3 fatty acids, arachidonic acid (AA; 20:4n6), dihomo-gamma-linolenic acid (DGLA; 20:3n6), gamma-linolenic acid (GLA; 18:3n6), linoleic acid (LA; 18:2n6) and total omega-6 fatty acids. Since summary data were available from multiple independent studies for each PUFA, we restricted our analyses to the single largest available study for each PUFA. This resulted in six of 10 instruments being defined using CHARGE and four of 10 being defined using UK Biobank. Due to methodological differences in how fatty acids were measured between the two studies (gas chromatography in CHARGE and nuclear magnetic resonance [NMR] in UK biobank) and the substantially different sample sizes (up to 114,999 in UK Biobank and up to 8866 in CHARGE), this instrument selection strategy reduces the comparability of Mendelian randomization (MR) results amongst different PUFA exposures. However, for our purposes we were not interested in comparing results between PUFA exposures, but mainly in determining whether associations with cancer exist for each PUFA.

Weak instrument bias (a type of measurement error) can also introduce bias, and its impact is likely to be more severe for instruments defined using CHARGE than for UK Biobank. (due to the relatively much smaller sample size of the former). In the presence of sample overlap, instruments estimated in small samples can introduce bias in MR estimates towards the confounded observational association, but in the absence of overlap this bias is towards the null. The degree of bias towards the confounded observational association depends on sample size, the degree of overlap of the instrument study (CHARGE or UK Biobank) with the cancer cases, and the size of the instrument effect on the exposure. Bias is expected to be minimal when study overlap is only with controls of a case-control study or when instrument strength is strong (conventionally defined as instrument F statistics much greater than 10)(1,2).

The individual cohorts of the CHARGE study were: ARIC, CHS, InCHIANTI, CARDIA and MESA. Given that these are population-based cohort studies in which the number of cancer cases as a proportion of the whole study is relatively low, we expect overlap with the cancer consortia, which are primarily based on case-control studies, to have been minimal, and we did not identify any instances of study overlap with the cancer consortia. Instrument strength in CHARGE also varied from 36 to 1929 (median=125, **Supplementary table S2**), indicating that even in the presence of considerable sample overlap, bias is unlikely to have been substantial(1). The UK Biobank study used to define instruments for PUFA exposures contained up to 114,999 participants. Of the 67 cancer outcomes investigated in primary MR analyses, 25 overlapped with UK Biobank. In these 25 studies, we estimate that 15% to 32% (median=24%) of the participants in the cancer study overlapped with the UK Biobank PUFA study (overlap of cases is likely to have been substantially lower) (**Supplementary table S5**). The estimated F statistics for the genetic instruments defined in UK Biobank varied from 81 to 374 (median=117, **Supplementary table S2**), indicating that bias from sample overlap with UK Biobank is likely to have been minimal(1). A caveat is that these F statistics may be over-estimated, indicating that we can’t entirely rule out some materially important weak instrument bias. However, given that overlap of CHARGE and UK Biobank with most of the cancer studies is likely to be low, this bias is more likely to be towards the null for most results.

## Clumping procedure to identify independent genetic associations

We identified independent SNPs associated with PUFA exposures (either the fatty acid biomarkers of D5D and D6D activity or individual omega 3 and omega 6 PUFAs), using a conventional threshold of GWAS statistical significance (P<5x10^-8^) and with linkage disequilibrium (LD) clumping to prune for independence. We used an r^2^ threshold of 0.001 and a base pair window of 10,000 kb. For European ancestry studies, we used 10,000 randomly selected individuals from UK Biobank as the reference panel (all analyses involving individual level data from UK Biobank were accessed under project number 15825). For East Asian ancestry studies, we used 504 East Asian ancestry individuals from the 1000 genomes project as the reference panel(3).

## Instrument for PUFA desaturase activity

The C allele of rs174546 is associated with higher PUFA desaturase activity in European and East Asian ancestry studies (**Supplementary table S1**). It is also associated with higher AA and higher EPA levels. In European and East Asian ancestry studies from Japan and Northern China, the C allele is also the major allele (T allele is the minor allele). However in South East Asian populations the C allele tends to be the minor allele and the T allele the major allele. FADS1/D5D converts the omega 6 PUFAs DGLA into AA and omega 3 PUFAs eicosatetraenoic acid (ETA) to EPA, while FADS2/D6D converts the omega 6 PUFAs LA into gamma linolenic acid (GLA) and omega 3 PUFAs ALA into stearidonic acid (SA).

## Defining genetic instruments for secondary exposures

Secondary Exposures: We defined a set of secondary exposures as omega 3 or omega 6 PUFAs that could be instrumented by genetic variants outside the FADS region, defined as genomic coordinates [GRCh37] chr11: 61,060,452-62,159,523. To do this, we obtained GWAS summary data for individual PUFAs from the following six studies: TwinsUK/KORA (Cooperative Health Research in the Region Augsburg)(4), the SCHS(5), Kettunen et al(6), the Framingham study(7), CHARGE^16^ and UK Biobank (downloaded from OpenGWAS <https://gwas.mrcieu.ac.uk/>)(8,9). Eighteen PUFAs were measured across the six studies (**Supplementary table S2**). Four of the 18 PUFAs could not be clearly classified as omega 3 or omega 6 PUFAs and were therefore excluded **(Supplementary table S2**). We next identified SNPs associated with the individual PUFAs using LD clumping (details described above). When multiple studies were available for the same PUFA, we restricted our analyses to the single largest available study for that PUFA (potential bias from this instrument selection strategy, which we consider to be minimal, is discussed above). For studies of European ancestry, this procedure identified 124 SNPs associated with 14 PUFAs. Four of the 14 PUFAs could not be instrumented by genetic variation outside the *FADS* region and were therefore excluded from secondary MR analyses. The retained secondary PUFA exposures included five omega 3 fatty acids (variation explained, with or without the FADS region in brackets): ALA (3.76% vs 0.36%), DHA (5.07% vs 1.58%), DPA (9.93% vs 2.52%) and total omega-3 fatty acids (8.94% vs 2.19%). Secondary PUFA exposures also included the following five omega 6 PUFAs: GLA (5.31% vs 1.94%), LA (4.01% vs 2.39%), AA (30.9% vs 0.47%), DGLA (11.86% vs 3.75%) and total omega-6 fatty acids (4.62% vs 4.59%). All instruments excluding the FADS region had an F statistic ≥36 (median=81; max=150, **Supplementary table S2**), indicating that bias from weak instruments is unlikely to be substantial(1). Seven of 10 secondary PUFA exposures could be instrumented by ≥2 independent SNPs outside of the FADS region, while three could be instrumented by a single SNP outside the FADS region. For studies of East Asian ancestry, only one PUFA was associated with variation outside the *FADS* region. We therefore excluded studies of East Asian ancestry from secondary MR analyses. Further details on these PUFAs and their genetic instruments can be found in **Supplementary tables S2-S3.**

## Combining results across studies

When summary data were available for the same cancer from multiple independent studies, we conducted MR analyses separately for each study, and then combined the MR results by fixed effects meta-analysis using inverse variance weights (“MR-by-study” approach). An alternative approach would be to first combine the summary data across studies, by fixed effects meta-analysis, and then to conduct MR analyses on the pooled dataset (“MR-of-pooled-study” approach). In our case, the first approach (MR-by-study) was preferable because for some cancers we combined results across European and Easy Asian ancestry studies and the instrument for PUFA desaturase activity has a different effect size in European and East Asian populations. The alternative approach is less appropriate when combining data across different populations with distinct instruments. Both approaches should however give the same result when only a single variant is used to instrument an exposure, as was the case in our primary analyses, but might be expected to give different results when the instrument is comprised of multiple variants, as was the case in secondary analyses. To see if that was the case, we compared the two approaches in an MR analysis of secondary PUFAs and lung cancer, where summary genetic data were derived from ILCCO and UK Biobank. The two approaches gave almost identical results (**Supplementary figure S21**).

## Making allowance for sample overlap between studies

We made allowance for sample overlap in analyses that combined results from, or tested for differences between, studies. In meta-analyses of cancer results from the same biological system or in meta regression analyses, we pruned MR results to have no overlapping cases (we acknowledge that a small degree of overlap, even after this procedure, cannot be entirely excluded). This involved the following steps. First, we pruned MR results so that only a single cancer was included for each of the following sites: breast, bowel, stomach, kidney, liver, mouth & throat, ovary and prostate, retaining the cancer with the most cases (e.g. overall breast cancer was included but breast cancer subtypes were excluded). Due to considerable overlap in cases amongst different cancer definitions in UK Biobank, we excluded the following cancer datasets generated in UK Biobank: overall cancer (case overlap with all site specific cancers), cancer of digestive organs (overlap with site-specific digestive system cancers), respiratory and intrathoracic cancer (overlap with lung cancer), lymphoid leukaemia (overlap with leukaemia), brain cancer (overlap with central nervous system and eye cancer), female genital cancer (overlap with endometrial cancer), non-melanoma skin cancer (overlap with squamous cell carcinoma and basal cell carcinoma), overall skin cancer (overlap with melanoma, basal cell carcinoma and squamous cell carcinoma), urinary tract cancer (overlap with bladder cancer). Further details on the above cancers can be found in **Supplementary tables 4-6**.

There was considerable overlap in controls amongst cancer datasets derived from the following biobanks or consortia: UK Biobank, FinnGen, Biobank Japan and InterLymph. To make allowance for control overlap, we increased the standard errors for all MR results generated with participants from one of the latter studies, using a decoupling transformation(10). This involved the following steps. First, we estimated a correlation matrix for the MR results using the method of Lin and Sullivan(11) (this step involves using the number of overlapping samples to infer the correlation matrix). We then used the correlation matrix to decouple the log odds ratios and standard errors for MR results with overlapping controls. The decoupled log odds ratios and standard errors were then used in the following downstream analyses: meta-regression analyses to identify sources of heterogeneity in MR results amongst cancers; random effects meta-analyses of MR results from the same biological system (reproductive cancers, urinary cancers, blood cancers and nervous system cancers); and in Q and Z tests for differences in MR findings amongst colorectal and lung cancer tumour subtypes.

## Sensitivity analyses for violations of Mendelian randomization analytical assumptions

We conducted three sets of analyses to assess the sensitivity of our main findings to violations of analytical assumptions (name of assumption in backets): colocalisation analysis (no genomic confounding), within-sibship MR analyses (no confounding by population stratification) and effect decomposition analyses (no horizontal pleiotropy with smoking).

### Colocalisation analysis

To assess the sensitivity of our findings to genomic confounding, we used colocalisation analysis to estimate posterior probabilities for the sharing of the same causal variant across selected cancers, PUFA desaturase biomarkers and *FADS1/2* gene expression(12). For these analyses we used genetic association results across 500,000 base pairs at the *FADS* gene cluster on chromosome 11q12.2-13.1 and focused on the cancers identified in the primary MR analysis. Genetic association results for *FADS1* and *FADS2* gene expression in Europeans were obtained from the Genotype-Tissue Expression (GTEx) project (version 8) for liver, adipose and blood tissues(13) and from eQTLGen in blood(14). Genetic association results for *FADS1* and *FADS2* gene expression in East Asians were obtained from Biobank Japan in blood and white blood cell subfractions (B cells, CD8 T cells, monocytes and natural killer [NK] cells; other tissues were unavailable)(14). We also selected tissues that were most representative of cancer sites associated with the PUFA desaturase biomarker in the primary MR analysis. Colocalisation analyses were conducted using the coloc package(12) and were conducted in a pairwise fashion, testing each cancer against each of three exposure traits: the PUFA desaturase biomarker, *FADS1* gene expression or *FADS2* gene expression. We set the prior probability that a randomly selected SNP was associated with the cancer or exposure trait to 1x10^-4^ or with both traits to 1x10^-6^ (these are the default priors of the coloc package)(12). Regional association plots, generated using the gassocplot package, were used to visualise genetic association results(15). We defined strong evidence for colocalisation as a poster probability (PP) for sharing the same causal variant (hypothesis 4 [H4])≥80%). Weak evidence was defined as PP_H4_≤25% and moderate evidence defined as 25%<PP_H4_<80%.

### Within-sibship MR analyses

To assess the sensitivity of our results to confounding by population stratification, indirect genetic effects or assortative mating, we conducted within-sibship MR analyses using data on 19,588 sibships from UK Biobank(16,17). Analyses were conducted on overall cancer (to boost power), overall skin cancers, colorectal cancer and lung cancer. To boost power, we also combined colorectal, lung and skin cancers.

### Effect decomposition analyses

In effect decomposition analyses, we estimated the association of rs174546 with 36 biomedically important characteristics, including lipids, smoking and anthropometrics, and then modelled the extent to which any identified associations (defined as P values <0.0013 [alpha of 0.05/36]) could explain our findings. The 36 selected characteristics were defined as traits categorised as risk factors in Open GWAS (<https://gwas.mrcieu.ac.uk/>)(9). For non-smoking characteristics associated with rs174546, we then searched for evidence that the identified characteristics are causally associated with colorectal cancer, lung cancer and basal cell carcinoma using a systematic review of MR studies of cancer(18). We then estimated the extent to which the identified characteristics could explain the association between rs174546 and the latter cancers using the product of coefficients method(19). The result of this analysis can be interpreted as the association between rs174546 and cancer mediated by the selected characteristic (also known as the indirect effect).

For smoking characteristics, we conducted additional association analyses of rs174546 and rs2524299 (the variant most strongly associated with lung cancer in the *FADS* region) with cigarettes smoked per day (N=249,752) and smoking status (ever versus never, N=607,291) in GSCAN (GWAS & Sequencing Consortium of Alcohol and Nicotine use), and with a lifetime smoking score in UK Biobank (N=462,690). The lifetime smoking score captures information on smoking status (ever versus never) as well as smoking duration, heaviness and cessation in ever smokers. Genetic instruments based on the lifetime smoking score can therefore be applied to samples that have not been stratified on smoking status, which was the case for all cancers included in our study with the exception of lung cancer. To assess whether smoking could account for our primary MR findings, we then conducted additional MR analyses of lifetime time smoking on colorectal cancer, lung cancer and basal cell carcinoma, as well as cigarettes smoked per day on lung cancer in ever smokers, using a random effects IVW model(8) (see **Supplementary tables S15 & S16** for details of genetic instruments). We then estimated the effect of rs174546 and rs2524299 on cancer mediated by the latter smoking characteristics using the products of coefficient method(19).

In a scenario in which the biomedical characteristic can account for our MR findings, we expect the mediated effect to be similar to the observed effect of rs174546 or rs2524299 on cancer (also known as the total effect)(19). A limitation is that, even in a scenario in which the effect is entirely mediated by the characteristic, due to measurement error we do not expect them to be identical. We tested for a difference between the mediated (i.e. indirect) and total effects using a Z test. We interpreted similarity between the mediated and total effects as compatible with either vertical or horizontal pleiotropy in our MR findings, whereas dissimilarity was interpreted as evidence against both types of pleiotropy. When we could not identify published MR studies of the characteristic and cancer, we estimated our own causal effect using the Wald ratio method (the effect of rs174546 on cancer divided by its effect on the characteristic). This can be interpreted as the expected effect of the characteristic on cancer, assuming the relationship between rs174546 and cancer is entirely mediated by that characteristic, rs174546 is truly associated with the characteristic, and that rs174546 is not associated with confounders of the relationship between the characteristic and cancer.

## Modelling sources of heterogeneity in MR findings amongst cancer sites

To identify potential sources of heterogeneity in MR findings amongst cancers, we assessed the impact of cancer-level characteristics on our results using a meta-regression approach. We modelled the following cancer-level characteristics: smoking (i.e. whether smoking is one of the accepted causes of the cancer(20,21)), chronic inflammation (whether the cancer has an accepted relationship to a chronic inflammatory condition(22)), cancer incidence(23), survival time(23), median age at diagnosis(23) and tissue-specific rates of stem cell division(24). **Supplementary table S17** contains further details on the assignment of cancer-level characteristics to each cancer included in these analyses.

We regressed the MR results for each cancer on the cancer-level characteristic and tested for a non-zero slope using a random effects model (fitted using a maximum-likelihood estimator and inverse variance weights implemented in the metafor package(25)). In this analysis, the MR result for each cancer can be considered the outcome, the cancer-level characteristic is the independent variable and a non-zero slope can be interpreted as evidence that the magnitude of the MR result varies by the cancer-level characteristic. To make allowance for sample overlap between cancer studies, we first pruned the MR results to have minimal overlap in cases and, in sensitivity analyses, inflated the standard errors using a decoupling transformation(10) (see above for more details). Sensitivity analyses further assessed the impact of reclassifying cancers into alternative groupings. These included: adding the result for laryngeal squamous carcinoma (identified by a search of the GWAS catalog) to the group of smoking-related cancers (this cancer was otherwise excluded from all analyses); recoding endometrial cancer (which shows a protective association with smoking in observational studies) as a smoking-related cancer; and including cancers associated with infectious agents in the chronic inflammatory group of cancers (see below for further details of how we defined this group).

We defined “smoking-related” cancers using the 2014 US Surgeon General’s Report(20,21). According to the report, the evidence is sufficient to infer that smoking causally increases the risk of the following 12 cancers: bladder cancer, cervical cancer, colorectal cancer, esophageal cancer, kidney cancer, laryngeal cancer, acute myeloid leukemia, liver cancer, lung cancer, oral cavity and pharyngeal cancer, pancreatic cancer and stomach cancer. We defined these cancers as “smoking-related” i.e. smoking is one of the accepted causes of the cancer. According to the report, there is either no, or insufficient, evidence to infer that smoking causally increases risk of the following cancers: brain cancer, breast cancer, prostate cancer, ovarian cancer and endometrial cancer. The report also concluded that smoking reduces risk of endometrial cancer in postmenopausal women. All cancers that did not overlap with the 12 “smoking-related cancers” above were defined as cancers with an uncertain relationship to smoking, referred to as “non-smoking cancers” for short. We had summary data for all smoking-related cancers, except for laryngeal cancer. However, our genetic instrument (rs174546) is in strong LD (r^2^=0.93) with a published GWAS hit for laryngeal cancer in East Asians(23). We therefore conducted additional meta-regression analyses with this cancer included as a sensitivity analysis.

We also compared cancers grouped according to their relationship to chronic inflammatory conditions as defined by Coussens et al(22). Although this may be an outdated reference, we consider it to be reasonably accurate for these analyses, which we consider to be exploratory and hypothesis-generating. According to Coussens et al(22), chronic inflammatory conditions increase risk of the following cancers (condition in brackets): mesothelioma (asbestosis & silicosis), lung cancer (bronchitis), bladder cancer (cystitis & bladder inflammation), oral squamous cell carcinoma (gingivitis and lichen planus), colorectal cancer (inflammatory bowel disease: Crohn’s disease & ulcerative colitis), vulvar squamous cell carcinoma (lichen sclerosus), pancreatic cancer (pancreatitis), esophageal cancer (reflex esophagitis & Barett’s esophagus), salivary gland carcinoma (sialadentis), mucosa-associated lymphoid tissue (MALT) lymphoma (Sjogren syndrome & hashimoto’s thyroiditis) and melanoma (skin inflammation). We defined these cancers as “chronic inflammatory cancers”. All cancers that did not overlap with this group were defined as “other inflammatory cancers”. Summary data were available for all “chronic inflammatory” cancers, except for vulvar squamous cell carcinoma and salivary gland carcinoma. As a sensitivity analysis, we expanded the “chronic inflammatory” group to include cancers with a relationship to infectious agents (agent in brackets): cholangiosarcoma and colon carcinoma (liver flukes); gall bladder cancer (bacteria); gastric adenocarcinoma, gastric MALT lymphoma (Helicobacter pylori); Hepatocellular carcinoma (Hepatitis B and/or C virus); B-cell non-Hodgkin lymphoma, Burkitt lymphoma (Epstein-Barr Virus); Non-Hodgkin lymphoma, squamous cell carcinomas, Kaposi’s sarcoma (human immunodeficiency virus, human herpesvirus type 8); skin carcinoma in draining sinuses (bacterial infection); ovarian carcinoma, cervical/anal carcinoma (Gonnorrhoea, chlamydia, human papillomavirus); bladder, liver and rectal carcinoma and follicular lymphoma of the spleen (Schistosomiasis). Summary data were available for all “infectious agent related” cancers except gall bladder cancer, Burkitt lymphoma and anal carcinoma.

A caveat is that the meta-regression procedure is likely to provide overly precise confidence intervals(26), and findings are also likely to be susceptible to confounding by other cancer-level characteristics. We therefore interpret results as exploratory and hypothesis-generating. We used an alpha threshold of 0.05 to prioritise potential sources of heterogeneity for replication in future studies.

## Note on how number of cases and controls was estimated

Our primary analyses relied on summary data corresponding to 67 cancers, 128 datasets and 43 independent biobanks or consortia. Given that many cancers and datasets were generated from the same biobank or consortium, there is potential to over-estimate the total number of cases and controls in these analyses. To estimate the number of independent cases and controls across the primary MR analyses, we retained a single cancer per consortium or biobank, retaining the cancer that had the largest number of cases. This led to an estimate of 562,871 cases and 1,619,465 controls.

## Note on esophageal squamous cell carcinoma

Although reported as esophageal cancer in Biobank Japan, 88% of esophageal cancer cases in Japan are squamous cell carcinomas(27). We therefore combined MR results for esophageal cancer from Biobank Japan (FAMRC ID=14) with MR results for esophageal squamous cell carcinoma from the N-UGC (FAMRC ID=99) and interpreted the outcome as esophageal squamous cell carcinoma.

# Supplementary results

## Note on results for skin cancers

Primary MR analyses identified associations with non-melanoma skin cancer, basal cell carcinoma, and overall skin cancer. Since we did not identify associations with squamous cell skin cancer or melanoma, we interpreted the associations with overall skin cancer and non-melanoma skin cancer as being driven by basal cell carcinoma. For this reason, of the skin cancer associations identified, only basal cell carcinoma was carried forward for sensitivity analyses. However, it should be noted that associations for melanoma and squamous cell skin cancer were not substantially different to the association for basal cell carcinoma (P=0.23 for difference with squamous cell carcinoma and P=0.82 for difference with melanoma).

## Error in the MR analysis of non-Hodgkin lymphoma

The presented MR results for non-Hodgkin lymphoma mistakenly included summary data from the BC-NHL study of B cell non-Hodgkin lymphoma (FAMRC ID=5). Due to ambiguous effect allele information, this study should have been excluded. However, excluding this study made little difference to the results. The odds ratio (95% confidence interval) for non-Hodgkin lymphoma per SD increase in genetically proxied PUFA desaturase activity was 0.98 (0.88 to 1.09), including the BC-NHL study (FAMRC ID =5) and was 0.97 (0.87 to 1.09) excluding it. The other studies included in the presented MR results for non-Hodgkin lymphoma were the FinnGen study of non-Hodgkin lymphoma unspecified (FAMRC ID=49) and the InterLymph study of Marginal zone lymphoma (FAMRC ID=86) (see **Supplementary tables S4-S6** for further details of these studies).

## Sensitivity analyses for violations of Mendelian randomization analytical assumptions

### Colocalisation

As a sensitivity analysis for genomic confounding, we assessed evidence for colocalisation of selected cancers with PUFA desaturase activity and with expression of the *FADS1* and *FADS2* genes in various tissues (**Supplementary figure S10 and Supplementary table S10**). Overall, the evidence for colocalisation was strongest for colorectal cancer: the posterior probabilities for a shared causal variant (PP_H4_) with PUFA desaturase activity and with *FADS1* gene expression in the sigmoid colon were ≥96% in European ancestry studies (with similarly strong evidence in East Asian ancestry studies). Findings for esophageal squamous cell carcinoma also showed strong evidence for colocalisation with PUFA desaturase activity (PP_H4_=87%), as well as with *FADS1* and *FADS2* gene expression in blood subtypes (PP_H4_≥76% in CD4 T, CD8 T and NK cells), in studies of East Asian ancestry. Further supporting colocalisation, the lead variants for esophageal squamous cell carcinoma and for *FADS1* gene expression in esophageal tissue are strongly correlated with each other in studies of European and East Asian ancestry (r^2^>0.8).

Colocalisation evidence for other cancers was less robust. Lung cancer did not consistently colocalise with different biomarkers for PUFA desaturase activity (PP_H4_=42% for the D5D biomarker and PP_H4_=68% for the D6D biomarker) and evidence for colocalisation with *FADS1* gene expression in lung tissue was moderate (PP_H4_=69%). On the other hand, there was strong evidence for colocalisation of lung cancer with *FADS1* gene expression in adipose subcutaneous tissue (PP_H4_=96%). Basal cell carcinoma did not colocalise with PUFA desaturase activity, *FADS1* gene expression or *FADS2* gene expression (PP_H4_<8%).

### Effect decomposition analyses

We found that the instrument for PUFA desaturase activity (rs174546) was weakly associated (SD change [95% confidence interval] per copy of the C allele) with LDL cholesterol (0.01 [0.01 to 0.02]), total cholesterol (0.05 [0.04 to 0.05]), triglycerides (-0.05 [-0.05 to -0.04]), HDL cholesterol (0.04 [0.03 to 0.05]), height (0.01 [0.01 to 0.02]), platelet count (-0.03 [-0.04 to -0.02]), heart rate (-0.03 [-0.04 to -0.02]) and age at menopause (0.02 [0.01 to 0.03]) **(Supplementary table S11 and Supplementary figure S12**). By comparison, the SD change in AA:DGLA (biomarker for D5D activity) per copy of the C allele was 0.87 (0.84 to 0.89) in European ancestry individuals (**Supplementary table S1**).

In a systematic review of MR studies(18), the OR (95% CI) for colorectal cancer per SD increase in genetically proxied height was 1.04 (1.00 to 1.08), for LDL cholesterol was 1.14 (1.04 to 1.25), for triglycerides was 0.93 (0.84 to 1.03), for total cholesterol was 1.09 (1.01 to 1.18), for HDL cholesterol was 1.03 (0.93 to 1.15) and for age at menopause was 1.00 (0.82 to 1.22). For lung cancer, the OR (95% CI) per SD increase in genetically proxied height was 1.07 (1.00 to 1.15), for platelet count was 1.00 (1.00 to 1.00), for LDL cholesterol was 0.90 (0.84 to 0.97), for triglycerides was 0.98 (0.91 to 1.06), for total cholesterol was 0.94 (0.88 to 1.01) and for HDL cholesterol was 1.01 (0.95 to 1.08)(18). We then estimated the effect of rs174546 on colorectal cancer and lung cancer mediated by the latter characteristics (also known as the indirect effect, **Supplementary table S11**). These were consistently less than 1.01 and smaller than the total effect of rs174546 on colorectal cancer (OR=1.06 95% CI: 1.05 to 1.08) and lung cancer (OR=1.03 95% CI: 1.01 to 1.06) (P values for difference between indirect and total effects ≤ 0.013). These results indicate that the identified characteristics cannot account for the observed associations of rs174546 with colorectal cancer and lung cancer and, consequently, that pleiotropy with these characteristics cannot explain our MR findings. A caveat is that we have likely underestimated the CIs for the indirect effects, since these analyses did not take into account uncertainty in the effect of rs174546 on the above characteristics.

We did not identify published MR studies for associations of platelet count and heart rate with colorectal cancer or associations of heart rate and age at menopause with lung cancer. We were therefore unable to estimate indirect effects for these characteristics. However, to account for the observed association of rs174546 with colorectal cancer, lower platelet count and lower heart rate would have to be associated with unusually strong ORs for colorectal cancer of 8.50 (4.59 to 15.72) per SD decrease. We therefore consider it unlikely that these characteristics can account for our colorectal cancer MR results. ORs for lung cancer per SD decrease in heart rate would have to be 3.06 (1.31 to 7.17) and per SD increase in age at menopause would have to be 5.52 (95% CI 1.51 to 20.24), which includes plausible effect sizes in the confidence intervals. We therefore cannot exclude the possibility that these characteristics can partly explain the lung cancer findings.

We conducted additional effect decomposition analyses to assess whether our findings could be explained by smoking. Per copy of the allele associated with higher PUFA desaturase activity (allele C) for rs174546, the OR for ever smoking status was 1.00 (95% CI: 0.99 to 1.01), the SD change in cigarettes smoked per day was 0.01 (95% CI 0.00 to 0.03) and the SD change in a lifetime smoking score was -0.001 (-0.004 to 0.002). In effect decomposition analyses, we found that genetically proxied lifetime smoking could not account for the observed association between rs174546 and selected cancers (P values ≤ 4.72x10^-03^ for difference between total and indirect smoking effects) (**Supplementary table S12**). In further analyses of rs2524299 (the strongest variant for lung cancer in the *FADS* region), the OR for lung cancer in ever smokers due to genetically proxied cigarettes smoked per day (1.01 [95% CI: 1.01 to 1.02]) was smaller than the observed association (OR=1.10 [95% CI: 1.05 to 1.14]) (P value = 3.53x10^-04^ for difference between total and indirect effects), suggesting that pleiotropy with smoking heaviness does not explain our MR results; similar findings were observed for rs174546 (**Supplementary table S13**). A caveat is that these analyses did not model uncertainty in the effect of these genetic variants on smoking, meaning we are likely to have underestimated the CIs for the indirect effects.

**Modelling sources of heterogeneity amongst cancers**

There was little evidence that MR results varied by cancer incidence, survival time, median age-at-diagnosis, or tissue-specific rates of stem cell division (P≥0.56, **Supplementary figures S13-S16; Supplementary table S14**). MR results tended to be stronger for 13 “smoking-related” cancers (OR= 1.05 [95% CI: 1.02 to 1.09]) compared to 30 “non-smoking” cancers (OR=1.01 [95% CI: 0.99 to 1.03]) (P = 0.003, for difference between ORs) (**Supplementary figure S17**). MR results were stronger for nine cancers with an accepted relationship to chronic inflammatory conditions (OR=1.05 [95% CI: 1.02 to 1.09]) compared to 34 other cancers (OR=1.01 [95% CI: 0.99 to 1.03]) (P=0.004, for the difference in OR) (**Supplementary figure S18**). MR results also tended to be stronger for digestive (OR=1.05 [95% CI: 1.01 to 1.10]) compared to non-digestive system cancers (OR=1.01 [95% CI: 0.99 to 1.03]) (P=0.019 for difference in OR) (**Supplementary figure S19**). Results were similar in various sensitivity analyses, including analyses that adjusted for potential sample overlap between studies or that reclassified cancers into different groupings (**Supplementary table S14**).

# Supplementary discussion

## Proxying multiple independent points in the PUFA biosynthesis pathway

Our analysis would have been significantly strengthened by the use of multiple independent genetic proxies for different points in the PUFA biosynthesis pathway, e.g. for the metabolic steps catalysed by the enzymatic products of *FADS1*, *FADS2* and *ELOVL2* (**Supplementary figure S2**). This proved impossible for *FADS1* and *FADS2,* due to their very close physical proximity. We did however employ some SNPs as instruments that probably work through modulation of *ELOVL2* gene expression levels. For example, rs3734398, rs3798713 and rs2180725, located in the *ELOVL2* gene, were amongst the genetic instruments used for docosapentaenoic acid (DPA), eicosapentaenoic acid (EPA) and linoleic acid (LA) (**Supplementary table S3**). rs3734398 resides in the 3 prime untranslated region, while rs3798713 and rs2180725 are intronic to *ELOVL2*. Since EPA is the substrate for the ELOVL2 enzyme, associations between rs3798713 and this fatty acid may reflect ELOVL2 activity levels. Per SD increase in EPA proxied by rs3798713, the odds ratio for colorectal cancer was 0.99 (95% confidence interval 0.84 to 1.17) (**Figure 2**), for lung cancer was 1.09 (0.89 to 1.33) (**Figure 3**) and for basal cell carcinoma was 0.99 (0.8 to 1.22) (**Supplementary figure S9**; MR results for EPA excluding the FADS region reflect rs3798713). Although these results could be interpreted as indicating no association between ELOVL2 enzyme activity and the selected cancers, interpretation is complicated by the relatively low variance explained in EPA levels by rs3798713 (0.55%). Thus, it is unclear whether this result reflects lack of power or is a true null. For example, the confidence intervals include an effect size that is similar in magnitude to the associations observed for arachidonic acid and colorectal cancer (**Figure 2**).

## Role of FADS activity

Our primary instrument, rs174546, which we used as a proxy for PUFA desaturase activity, resides in the 3 prime untranslated region of the *FADS1* gene and is also intronic to the *FADS2* gene. There is evidence that the variant regulates expression of both *FADS1* and *FADS2* through modulation of an enhancer element located between the two genes. For example, rs174537 (r2=0.989 with rs174546) is correlated with DNA methylation in a putative enhancer located between the *FADS1* and *FADS2* promoter regions(28). Our instrument, or the causal variant(s) in LD with our instrument, is likely to directly affect levels of D5D and D6D enzymes via *FADS1* and *FADS2* gene expression. Since we were unable to distinguish between activity of the two, we interpret our instrument as a proxy for both D5D and D6D desaturase activity.

D5D and D6D metabolise omega 3 and omega 6 PUFAs, which are hypothesised to have opposing effects on cancer risk - the omega 3 PUFAs having anti-inflammatory anti-carcinogenic effects and omega 6 PUFAs have pro-inflammatory pro-carcinogenic effects. These opposing effects would be expected to attenuate cancer findings based on *FADS* genetic variants towards the null. As to why we observed positive associations with some cancers (and not consistently null associations), this might reflect a relatively higher consumption of omega 6 PUFAs compared to omega 3 PUFAs in the European and East Asian ancestry studies included in our analyses. In other words, omega 6 PUFAs may outcompete omega 3 PUFAs for D5D and D6D, due to their greater abundance. Alternatively, perhaps the observational associations for omega 3 and cancer risk are not causal and only omega 6 PUFAs have a true causal effect on cancer. It would be interesting to replicate our study in a population where omega 3 PUFA consumption outweighs the consumption of omega 6 PUFAs, to see whether associations with cancer risk reversed direction or attenuated to the null.

## Clinical implications of global variation in rs174546 C allele prevalence

To improve the safety profile of potential interventions on PUFAs, interventions could be targeted to carriers of the C allele of rs174546 (the allele associated with higher PUFA desaturase activity, higher risk of selected cancers but lower risk of inflammatory bowel disease), since such individuals would be expected to obtain more benefit than carriers of the T allele. Such an intervention would have to consider variation in C and T allele prevalence. For example, the intervention would probably not be worth the increased cost of screening in populations where the T allele is very rare.

## Potential carcinogenic pathways

The pro-inflammatory eicosanoid pathway is not the only biological mechanism that could account for our findings. For example, AA is metabolised into leukotrienes and pro-inflammatory hydroxyeicosatetraenoic acids (HETEs), via lipoxygenase (LOX), which are hypothesised to have a role in carcinogenesis(28). Cytochrome P450 (CYP) ω-hydroxylase is also able to metabolise AA into HETEs(28). CYP epoxygenase generates AA epoxides or epoxyeicosatrienoic acids (EETs), which could play a role in carcinogenesis via their influence on cellular proliferation, survival and angiogenesis(28). Thromboxane, another product of COX mediated metabolism of AA, is a clotting factor that leads to platelet aggregation, which could influence carcinogenesis through its regulation of angiogenesis(29).

# Study acknowledgements

## 23andMe

23andMe, Inc. participants provided informed consent and volunteered to participate in the research online, under a protocol approved by the external AAHRPP-accredited IRB, Ethical & Independent (E&I) Review Services. As of 2022, E&I Review Services is part of Salus IRB (<https://www.versiticlinicaltrials.org/salusirb>).

The full GWAS summary statistics for the 23andMe discovery data set will be made available through 23andMe to qualified researchers under an agreement with 23andMe that protects the privacy of the 23andMe, Inc. participants. Datasets will be made available at no cost for academic use. Please visit <https://research.23andme.com/collaborate/#dataset-access/> for more information and to apply to access the data.

## Asian Colorectal Cancer Consortium (ACCC)

The ACCC is supported by the National Institutes of Health R01 CA188214. Members of the ACCC: Sun-Seog Kweon, Koichi Matsuda, Wei-Hua Jia, Aesun Shin, Keitaro Matsuo, Sun Ha Jee, Dong-Hyun Kim and Jeongseon Kim.

## Endometrial Cancer Association Consortium (ECAC)

We are grateful for Deborah Thompson’s integral contributions to the Endometrial Cancer Association Consortium. The endometrial cancer genome-wide association analyses were supported by the National Health and Medical Research Council of Australia (APP552402, APP1031333, APP1109286, APP1111246 and APP1061779), the U.S. National Institutes of Health (R01-CA134958), European Research Council (EU FP7 Grant), Wellcome Trust Centre for Human Genetics (090532/Z/09Z) and Cancer Research UK. OncoArray genotyping of ECAC cases was performed with the generous assistance of the Ovarian Cancer Association Consortium (OCAC), which was funded through grants from the U.S. National Institutes of Health (CA1X01HG007491-01, U19-CA148112, R01-CA149429 and R01-CA058598); Canadian Institutes of Health Research (MOP-86727) and the Ovarian Cancer Research Fund. We particularly thank the efforts of Cathy Phelan. OncoArray genotyping of the BCAC controls was funded by Genome Canada Grant GPH-129344, NIH Grant U19 CA148065, and Cancer UK Grant C1287/A16563. All studies and funders are listed in O’Mara et al (2018).

## The EPIdemiology of THYRoid cancer consortium (EPITHYR)

The EPITHYR genome-wide association analyses were supported by Institut National du Cancer (grant number 9533) and Fondation ARC (grant number PGA120150202302).

Esophageal Adenocarcinoma Study (EAS)

Esophageal adenocarcinoma GWAS(30): The MD Anderson controls were drawn from dbGaP (study accession: phs000187.v1.p1). Genotyping of these controls was performed through the University of Texas MD Anderson Cancer Center (UTMDACC) and the Johns Hopkins University Center for Inherited Disease Research (CIDR). We acknowledge the principal investigators of this study: Christopher Amos, Qingyi Wei and Jeffrey E. Lee. Controls from the Genome-Wide Association Study of Parkinson Disease were obtained from dbGaP (study accession: phs000196.v2.p1). This work, in part, used data from the National Institute of Neurological Disorders and Stroke (NINDS) dbGaP database from the CIDR:NeuroGenetics Research Consortium Parkinson’s disease study. We acknowledge the principal investigators and coinvestigators of this study: Haydeh Payami, John Nutt, Cyrus Zabetian, Stewart Factor, Eric Molho and Donald Higgins. Controls from the Chronic Renal Insufficiency Cohort (CRIC) were drawn from dbGaP (study accession: phs000524.v1.p1). The CRIC study was performed by the CRIC investigators and supported by the National Institute of Diabetes and Digestive and Kidney Diseases (NIDDK). Data and samples from CRIC reported here were supplied by NIDDK Central Repositories. This report was not prepared in collaboration with investigators of the CRIC study and does not necessarily reflect the opinions or views of the CRIC study, the NIDDK Central Repositories or the NIDDK. We acknowledge the principal investigators and the project officer of this study: Harold I. Feldman, Raymond R. Townsend, Lawrence J. Appel, Mahboob Rahman, Akinlolu Ojo, James P. Lash, Jiang He, Alan S. Go and John W. Kusek.

## Colon Cancer Family Registry-Colorectal Transdisciplinary study- Genetics and Epidemiology of Colorectal Cancer Consortium (CCFR-CORECT-GECCO)

We gratefully acknowledge the investigators of the CCFR, CORECT and GECCO consortia: Goncalo R Abecasis (GECCO), Demetrius Albanes (CORECT), M Henar Alonso (CORECT), Kristin Anderson (GECCO), Coral Arnau-Collell (CORECT & GECCO), Volker Arndt (CORECT & GECCO), Christina Bamia (GECCO), John A Baron (CORECT), Elizabeth L Barry (CORECT), Michael C Bassik (GECCO), Sonja I Berndt (GECCO), Stéphane Bézieau (GECCO), Stephanie Bien (GECCO), D Timothy Bishop (GECCO), Heiner Boeing (GECCO), Hermann Brenner (GECCO), Stefanie Brezina (GECCO), Stephan Buch (CORECT & GECCO), Daniel D Buchanan (CCFR), Andrea Burnett-Hartman (GECCO), Bette J Caan (GECCO), Qiuyin Cai (CORECT), Peter T Campbell (CORECT & GECCO), Christopher S Carlson (GECCO), Graham Casey (CCFR), Jose Esteban Castelao (CORECT), Sergi Castellví-Bel (GECCO), Andrew T Chan (GECCO), Jenny Chang-Claude (GECCO), Stephen J Chanock (GECCO), Sai Chen (GECCO), Lee Soon (CORECT), Maria-Dolores Chirlaque (GECCO), Sang Hee Cho (CORECT), James Church (CCFR), Gerhard Coetzee (CORECT), David V Conti (CORECT), Chiara Cremolini (CORECT), Amanda J Cross (GECCO), Marcia Cruz-Correa (CORECT), Katarina Cuk (CORECT), Keith R Curtis (GECCO), Albert de la Chapelle (GECCO), Kimberly F Doheny (GECCO), David Duggan (CCFR), Douglas F Easton (CORECT), Sjoerd G Elias (GECCO), Faye Elliott (GECCO), Dallas R English (CORECT), Alfredo Falcone (CORECT), Jane C Figueiredo (CCFR), Liesel M FitzGerald (CORECT), Charles Fuchs (GECCO), Manuela Gago-Dominguez (CORECT), Manish Gala (GECCO), Steven J Gallinger (CCFR), William Gauderman (CORECT), Graham G Giles (CORECT & GECCO), Edward Giovannucci (GECCO), Jian Gong (GECCO), Phyllis J Goodman (GECCO), William M Grady (CORECT & GECCO), Peyton Greenside (GECCO), Joel Greenson (CORECT), John S Grove (CCFR), Stephen B Gruber (CORECT), Andrea Gsur (GECCO), Marc J Gunter (GECCO), Robert W Haile (CCFR), Christopher A. Haiman (CORECT & GECCO), Jochen Hampe (CORECT & GECCO), Heather Hampel (GECCO), Sophia Harlid (GECCO), Tabitha A Harrison (GECCO), Richard B Hayes (GECCO), Volker Heinemann (CORECT), Philipp Hofer (GECCO), Michael Hoffmeister (GECCO), John L Hopper (CCFR), Li Hsu (GECCO), Wen-Yi Huang (GECCO), Thomas J Hudson (GECCO), David J Hunter (GECCO), Jeroen R Huyghe (GECCO), Gregory E Idos (CORECT), Rebecca Jackson (GECCO), Mark A Jenkins (CCFR), Jihyoun Jeon (GECCO), Amit D Joshi (GECCO), Corinne E Joshu (GECCO), Hyun Min Kang (GECCO), Temitope O Keku (GECCO), Timothy J Key (GECCO), Hyeong Rok Kim (CORECT), Laurence N Kolonel (CORECT & GECCO), Charles Kooperberg (GECCO), Tilman Kuhn (GECCO), Anshul Kundaje (GECCO), Sébastien Küry (GECCO), Sun-Seog Kweon (CORECT), Susanna C Larsson (CORECT), Cecelia A Laurie (GECCO), Loic Le Marchand (CCFR, CORECT & GECCO), Suzanne M Leal (GECCO), Soo Chin Lee (CORECT), Flavio Lejbkowicz (CORECT), Heinz-Josef Lenz (CORECT), David M Levine (GECCO), Christopher I Li (CORECT & GECCO), Li Li (CORECT), Wolfgang Lieb (CORECT & GECCO), Yi Lin (GECCO), Annika Lindblom (CORECT), Noralane M Lindor (CCFR), Hua Ling (CORECT), Yun-Ru Liu (CORECT), Tin L Louie (GECCO), Fotios Loupakis (CORECT), Frank Luh (CORECT), Satu Männistö (CORECT), Sanford D Markowitz (CORECT), Vicente Martín (GECCO), Giovanna Masala (CORECT), Kevin J McDonnell (CORECT), Caroline E McNeil (CORECT), Marilena Melas (CORECT), Roger L Milne (CORECT & GECCO), Victor Moreno (CORECT & GECCO), Lorena Moreno (GECCO), Bhramar Mukherjee (CORECT), Victor Muñoz-Garzón (GECCO), Neil Murphy (GECCO), Alessio Naccarati (CORECT), Sarah C Nelson (GECCO), Polly A Newcomb (CCFR), Deborah A Nickerson (GECCO), Kenneth Offit (CORECT), Shuji Ogino (GECCO), N Charlotte Onland-Moret (CORECT), Barbara Pardini (GECCO), Patrick S Parfrey (GECCO), Rachel Pearlman (GECCO), Vittorio Perduca (CORECT), Julyann Pérez-Mayoral (CORECT), Ulrike Peters (CORECT & GECCO), Paul D P Pharoah (CORECT), Mila Pinchev (CORECT), Elizabeth A Platz (GECCO), Sarah Plummer (CORECT), John D Potter (GECCO), Ross L Prentice (GECCO), Elizabeth Pugh (GECCO), Chenxu Qu (CORECT), Conghui Qu (GECCO), Leon Raskin (CORECT), Gad Rennert (CORECT), Hedy S Rennert (CORECT), Elio Riboli (GECCO), Miguel Rodríguez-Barranco (GECCO), Jane Romm (GECCO), Lori C Sakoda (GECCO), Peter C Scacheri (GECCO), Clemens Schafmayer (CORECT & GECCO), Stephanie L Schmit (CORECT), Robert E Schoen (GECCO), Fredrick R Schumacher (GECCO), Daniela Seminara (GECCO), Gianluca Severi (CORECT), Mitul Shah (CORECT), Tameka Shelford (GECCO), David Shibata (CORECT), Min-Ho Shin (CORECT), Xiao-Ou Shu (CORECT), Katerina Shulman (CORECT), Erin Siegel (CORECT), Sabina Sieri (CORECT), Nasa A Sinnott-Armstrong (GECCO), Martha L Slattery (GECCO), Joshua D Smith (GECCO), Melissa C Southey (CCFR), Zsofia K Stadler (CORECT), Mariana Stern (CORECT), Sebastian Stintzing (CORECT), Yu-Ru Su (GECCO), Catherine M Tangen (GECCO), Stephen N Thibodeau (CCFR), Duncan C Thomas (CORECT), Sushma S Thomas (GECCO), Amanda E Toland (GECCO), Antonia Trichopoulou (GECCO), Cornelia M Ulrich (CORECT), David J Van Den Berg (CORECT), Franzel JB van Duijnhoven (GECCO), Bethany Van Guelpen (GECCO), Henk van Kranen (CORECT), Joseph Vijai (CORECT), Kala Visvanathan (GECCO), Pavel Vodicka (GECCO), Ludmila Vodickova (GECCO), Veronika Vymetalkova (GECCO), Michael Wainberg (GECCO), Hansong Wang (CCFR), Korbinian Weigl (GECCO), Stephanie J Weinstein (CORECT), Emily White (GECCO), Lynne R. Wilkens (CORECT), Aung Ko Win (CCFR), C Roland Wolf (CORECT), Alicja Wolk (CORECT), Michael O Woods (CCFR), Anna H Wu (CORECT), Yen Yun (CORECT), Syed H Zaidi (GECCO), Brent W Zanke (GECCO), Wei Zheng (ACCC, CORECT & GECCO)

## InterLymph

The Health Professionals Follow-up Study was supported in part by National Institutes of Health grants UO1 CA167552, R01 CA149445, and R01 CA098122. The authors would like to acknowledge the contribution to this study from central cancer registries supported through the Centers for Disease Control and Prevention’s National Program of Cancer Registries (NPCR) and/or the National Cancer Institute’s Surveillance, Epidemiology, and End Results (SEER) Program. Central registries may also be supported by state agencies, universities, and cancer centers. Participating central cancer registries include the following: Alabama, Alaska, Arizona, Arkansas, California, Colorado, Connecticut, Delaware, Florida, Georgia, Hawaii, Idaho, Indiana, Iowa, Kentucky, Louisiana, Massachusetts, Maine, Maryland, Michigan, Mississippi, Montana, Nebraska, Nevada, New Hampshire, New Jersey, New Mexico, New York, North Carolina, North Dakota, Ohio, Oklahoma, Oregon, Pennsylvania, Puerto Rico, Rhode Island, Seattle SEER Registry, South Carolina, Tennessee, Texas, Utah, Virginia, West Virginia, Wyoming. The authors assume full responsibility for analyses and interpretation of these data. We would also like to thank the participants and staff of the Health Professionals Follow-up Study for their valuable contributions. The study protocol was approved by the institutional review boards of the Brigham and Women’s Hospital and Harvard T.H. Chan School of Public Health, and those of participating registries as required.

The Nurses’ Health Study was supported in part by National Institutes of Health grants UM1 CA186107, P01 CA87969, R01 CA49449, R01 CA149445, R01 CA098122 and R01 CA134958. The authors would like to acknowledge the contribution to this study from central cancer registries supported through the Centers for Disease Control and Prevention’s National Program of Cancer Registries (NPCR) and/or the National Cancer Institute’s Surveillance, Epidemiology, and End Results (SEER) Program. Central registries may also be supported by state agencies, universities, and cancer centers. Participating central cancer registries include the following: Alabama, Alaska, Arizona, Arkansas, California, Colorado, Connecticut, Delaware, Florida, Georgia, Hawaii, Idaho, Indiana, Iowa, Kentucky, Louisiana, Massachusetts, Maine, Maryland, Michigan, Mississippi, Montana, Nebraska, Nevada, New Hampshire, New Jersey, New Mexico, New York, North Carolina, North Dakota, Ohio, Oklahoma, Oregon, Pennsylvania, Puerto Rico, Rhode Island, Seattle SEER Registry, South Carolina, Tennessee, Texas, Utah, Virginia, West Virginia, Wyoming. The authors assume full responsibility for analyses and interpretation of these data. We also thank the participants and staff of the Nurses' Health Study for their valuable contributions. The study protocol was approved by the institutional review boards of the Brigham and Women’s Hospital and Harvard T.H. Chan School of Public Health, and those of participating registries as required.

The Utah hematological malignancy study was supported by funding from the National Cancer Institute (NCI) grant R01 CA134674 (to NJC). Data collection in Utah was supported by the Utah Population Database (UPDB) and Utah Cancer Registry (UCR). The UPDB is supported by Huntsman Cancer Institute (HCI, including Huntsman Cancer Foundation, HCF), the University of Utah, and NCI grant P30 CA2014. The UCR is funded by the NCI's SEER Program, Contract No. HHSN261201800016I, the US Center for Disease Control and Prevention's National Program of Cancer Registries (Cooperative Agreement No. NU58DP006320), the University of Utah, and HCF. The study thanks all study participants, ascertainment, laboratory, and research informatics teams at HCI, and the Hematology Biobank, Justin Williams, Brandt Jones, Myke Madsen, Brian Avery and Rob Sargent for their important contributions.

The Mayo studies in InterLymph were supported in part by the US National Cancer Institute grants P50 CA97274 and R01 CA92153.

## International Lung Cancer Consortium (ILCCO)

The authors gratefully acknowledge the following investigators and contributors to the International Lung Cancer Consortium: Demetrios Albanes (ATBC), Stephen Lam (Canadian screening study), Adonina Tardon (CAPUA STUDY), Chu Chen (CARET), Stig E. Bojesen (Copenhagen study), Maria Teresa Landi (EAGLE), Mattias Johansson (EPIC: European Prospective Investigation into Cancer and Nutrition), Angela Risch (German Lung Cancer Study - DKFZ), Heike Bickeböller (German Lung Cancer Study -LUCY), H-Erich Wichmann (German Lung Cancer Study - LUCY), David Christiani (Harvard Lung Cancer Study), Gadi Rennert (Israel study), Susanne Arnold (Kentucky LCRI-DOD), Paul Brennan and James McKay (L2-IARC), John K. Field (Liverpool Lung Project), Sanjay S. Shete (MDACC), Loic Le Marchand (MEC), Olle Melander (MDCS: The Malmö Diet and Cancer Study), Hans Brunnström (MDCS: The Malmö Diet and Cancer Study), Geoffrey Liu (MSH-PMH, Canadian Screening studies), Rayjean J. Hung (MSH-PMH), Angeline Andrew (NELCS), Lambertus A. Kiemeney (Nijmegen), Shan Zienolddiny-Narui (Norway), Kjell Grankvist (NSHDS: Northern Sweden Health and Disease Study), Mikael Johansson (NSHDS: Northern Sweden Health and Disease Study), Neil Caporaso (PLCO), Angie Cox (ReSoLucent), Philip Lazarus (Tampa Lung Cancer Study), Matthew B. Schabath (Total Lung Cancer (TLC): Molecular Epidemiology of Lung Cancer Survival), Melinda C. Aldrich (Vanderbilt Lung Cancer Study - BioVU).

## Melanoma Meta-analysis Consortium (MMAC)

Members of the MMAC:

[Law MH](https://www.ncbi.nlm.nih.gov/pubmed/?term=Law%20MH%5BAuthor%5D&cauthor=true&cauthor_uid=26237428)^1*^, [Bishop DT](https://www.ncbi.nlm.nih.gov/pubmed/?term=Bishop%20DT%5BAuthor%5D&cauthor=true&cauthor_uid=26237428)^2*^, [Lee JE](https://www.ncbi.nlm.nih.gov/pubmed/?term=Lee%20JE%5BAuthor%5D&cauthor=true&cauthor_uid=26237428)^3#^, [Brossard M](https://www.ncbi.nlm.nih.gov/pubmed/?term=Brossard%20M%5BAuthor%5D&cauthor=true&cauthor_uid=26237428)^4,5#^, [Martin NG](https://www.ncbi.nlm.nih.gov/pubmed/?term=Martin%20NG%5BAuthor%5D&cauthor=true&cauthor_uid=26237428)^6^, [Moses EK](https://www.ncbi.nlm.nih.gov/pubmed/?term=Moses%20EK%5BAuthor%5D&cauthor=true&cauthor_uid=26237428)^7^, [Song F](https://www.ncbi.nlm.nih.gov/pubmed/?term=Song%20F%5BAuthor%5D&cauthor=true&cauthor_uid=26237428)^8^, [Barrett JH](https://www.ncbi.nlm.nih.gov/pubmed/?term=Barrett%20JH%5BAuthor%5D&cauthor=true&cauthor_uid=26237428)^2^, [Kumar R](https://www.ncbi.nlm.nih.gov/pubmed/?term=Kumar%20R%5BAuthor%5D&cauthor=true&cauthor_uid=26237428)^9^, [Easton DF](https://www.ncbi.nlm.nih.gov/pubmed/?term=Easton%20DF%5BAuthor%5D&cauthor=true&cauthor_uid=26237428)^10^, [Pharoah PD](https://www.ncbi.nlm.nih.gov/pubmed/?term=Pharoah%20PD%5BAuthor%5D&cauthor=true&cauthor_uid=26237428)^11^, [Swerdlow AJ](https://www.ncbi.nlm.nih.gov/pubmed/?term=Swerdlow%20AJ%5BAuthor%5D&cauthor=true&cauthor_uid=26237428)^12,13^, [Kypreou KP](https://www.ncbi.nlm.nih.gov/pubmed/?term=Kypreou%20KP%5BAuthor%5D&cauthor=true&cauthor_uid=26237428)^14^, [Taylor JC](https://www.ncbi.nlm.nih.gov/pubmed/?term=Taylor%20JC%5BAuthor%5D&cauthor=true&cauthor_uid=26237428)^2^, [Harland M](https://www.ncbi.nlm.nih.gov/pubmed/?term=Harland%20M%5BAuthor%5D&cauthor=true&cauthor_uid=26237428)^2^, [Randerson-Moor J](https://www.ncbi.nlm.nih.gov/pubmed/?term=Randerson-Moor%20J%5BAuthor%5D&cauthor=true&cauthor_uid=26237428)^2^, [Akslen LA](https://www.ncbi.nlm.nih.gov/pubmed/?term=Akslen%20LA%5BAuthor%5D&cauthor=true&cauthor_uid=26237428)^15,16^, [Andresen PA](https://www.ncbi.nlm.nih.gov/pubmed/?term=Andresen%20PA%5BAuthor%5D&cauthor=true&cauthor_uid=26237428)^17^, [Avril MF](https://www.ncbi.nlm.nih.gov/pubmed/?term=Avril%20MF%5BAuthor%5D&cauthor=true&cauthor_uid=26237428)^18^, [Azizi E](https://www.ncbi.nlm.nih.gov/pubmed/?term=Azizi%20E%5BAuthor%5D&cauthor=true&cauthor_uid=26237428)^19,20^, [Scarrà GB](https://www.ncbi.nlm.nih.gov/pubmed/?term=Scarr%C3%A0%20GB%5BAuthor%5D&cauthor=true&cauthor_uid=26237428)^21,22^, [Brown KM](https://www.ncbi.nlm.nih.gov/pubmed/?term=Brown%20KM%5BAuthor%5D&cauthor=true&cauthor_uid=26237428)^23^, [Dȩbniak T](https://www.ncbi.nlm.nih.gov/pubmed/?term=D%C8%A9bniak%20T%5BAuthor%5D&cauthor=true&cauthor_uid=26237428)^24^, [Duffy DL](https://www.ncbi.nlm.nih.gov/pubmed/?term=Duffy%20DL%5BAuthor%5D&cauthor=true&cauthor_uid=26237428)^6^, [Elder DE](https://www.ncbi.nlm.nih.gov/pubmed/?term=Elder%20DE%5BAuthor%5D&cauthor=true&cauthor_uid=26237428)^25^, [Fang S](https://www.ncbi.nlm.nih.gov/pubmed/?term=Fang%20S%5BAuthor%5D&cauthor=true&cauthor_uid=26237428)^3^, [Friedman E](https://www.ncbi.nlm.nih.gov/pubmed/?term=Friedman%20E%5BAuthor%5D&cauthor=true&cauthor_uid=26237428)^20^, [Galan P](https://www.ncbi.nlm.nih.gov/pubmed/?term=Galan%20P%5BAuthor%5D&cauthor=true&cauthor_uid=26237428)^26^, [Ghiorzo P](https://www.ncbi.nlm.nih.gov/pubmed/?term=Ghiorzo%20P%5BAuthor%5D&cauthor=true&cauthor_uid=26237428)^21,22^, [Gillanders EM](https://www.ncbi.nlm.nih.gov/pubmed/?term=Gillanders%20EM%5BAuthor%5D&cauthor=true&cauthor_uid=26237428)^27^, [Goldstein AM](https://www.ncbi.nlm.nih.gov/pubmed/?term=Goldstein%20AM%5BAuthor%5D&cauthor=true&cauthor_uid=26237428)^23^, [Gruis NA](https://www.ncbi.nlm.nih.gov/pubmed/?term=Gruis%20NA%5BAuthor%5D&cauthor=true&cauthor_uid=26237428)^28^, [Hansson J](https://www.ncbi.nlm.nih.gov/pubmed/?term=Hansson%20J%5BAuthor%5D&cauthor=true&cauthor_uid=26237428)^29^, [Helsing P](https://www.ncbi.nlm.nih.gov/pubmed/?term=Helsing%20P%5BAuthor%5D&cauthor=true&cauthor_uid=26237428)^30^, [Hočevar M](https://www.ncbi.nlm.nih.gov/pubmed/?term=Ho%C4%8Devar%20M%5BAuthor%5D&cauthor=true&cauthor_uid=26237428)^31^, [Höiom V](https://www.ncbi.nlm.nih.gov/pubmed/?term=H%C3%B6iom%20V%5BAuthor%5D&cauthor=true&cauthor_uid=26237428)^29^, [Ingvar C](https://www.ncbi.nlm.nih.gov/pubmed/?term=Ingvar%20C%5BAuthor%5D&cauthor=true&cauthor_uid=26237428)^32^, [Kanetsky PA](https://www.ncbi.nlm.nih.gov/pubmed/?term=Kanetsky%20PA%5BAuthor%5D&cauthor=true&cauthor_uid=26237428)^33^, [Chen WV](https://www.ncbi.nlm.nih.gov/pubmed/?term=Chen%20WV%5BAuthor%5D&cauthor=true&cauthor_uid=26237428)^34^; [GenoMEL Consortium](https://www.ncbi.nlm.nih.gov/pubmed/?term=GenoMEL%20Consortium%5BCorporate%20Author%5D); [Essen-Heidelberg Investigators](https://www.ncbi.nlm.nih.gov/pubmed/?term=Essen-Heidelberg%20Investigators%5BCorporate%20Author%5D); [SDH Study Group](https://www.ncbi.nlm.nih.gov/pubmed/?term=SDH%20Study%20Group%5BCorporate%20Author%5D); [Q-MEGA and QTWIN Investigators](https://www.ncbi.nlm.nih.gov/pubmed/?term=Q-MEGA%20and%20QTWIN%20Investigators%5BCorporate%20Author%5D); [AMFS Investigators](https://www.ncbi.nlm.nih.gov/pubmed/?term=AMFS%20Investigators%5BCorporate%20Author%5D); [ATHENS Melanoma Study Group](https://www.ncbi.nlm.nih.gov/pubmed/?term=ATHENS%20Melanoma%20Study%20Group%5BCorporate%20Author%5D), [Landi MT](https://www.ncbi.nlm.nih.gov/pubmed/?term=Landi%20MT%5BAuthor%5D&cauthor=true&cauthor_uid=26237428)^23^, [Lang J](https://www.ncbi.nlm.nih.gov/pubmed/?term=Lang%20J%5BAuthor%5D&cauthor=true&cauthor_uid=26237428)^35^, [Lathrop GM](https://www.ncbi.nlm.nih.gov/pubmed/?term=Lathrop%20GM%5BAuthor%5D&cauthor=true&cauthor_uid=26237428)^36^, [Lubiński J](https://www.ncbi.nlm.nih.gov/pubmed/?term=Lubi%C5%84ski%20J%5BAuthor%5D&cauthor=true&cauthor_uid=26237428)^24^, [Mackie RM](https://www.ncbi.nlm.nih.gov/pubmed/?term=Mackie%20RM%5BAuthor%5D&cauthor=true&cauthor_uid=26237428)^35,37^, [Mann GJ](https://www.ncbi.nlm.nih.gov/pubmed/?term=Mann%20GJ%5BAuthor%5D&cauthor=true&cauthor_uid=26237428)^38^, [Molven A](https://www.ncbi.nlm.nih.gov/pubmed/?term=Molven%20A%5BAuthor%5D&cauthor=true&cauthor_uid=26237428)^16,39^, [Montgomery GW](https://www.ncbi.nlm.nih.gov/pubmed/?term=Montgomery%20GW%5BAuthor%5D&cauthor=true&cauthor_uid=26237428)^40^, [Novaković S](https://www.ncbi.nlm.nih.gov/pubmed/?term=Novakovi%C4%87%20S%5BAuthor%5D&cauthor=true&cauthor_uid=26237428)^41^, [Olsson H](https://www.ncbi.nlm.nih.gov/pubmed/?term=Olsson%20H%5BAuthor%5D&cauthor=true&cauthor_uid=26237428)^42,43^, [Puig S](https://www.ncbi.nlm.nih.gov/pubmed/?term=Puig%20S%5BAuthor%5D&cauthor=true&cauthor_uid=26237428)^44,45^, [Puig-Butille JA](https://www.ncbi.nlm.nih.gov/pubmed/?term=Puig-Butille%20JA%5BAuthor%5D&cauthor=true&cauthor_uid=26237428)^44,45^, Wu W^46,47^, [Qureshi AA](https://www.ncbi.nlm.nih.gov/pubmed/?term=Qureshi%20AA%5BAuthor%5D&cauthor=true&cauthor_uid=26237428)^48^, [Radford-Smith GL](https://www.ncbi.nlm.nih.gov/pubmed/?term=Radford-Smith%20GL%5BAuthor%5D&cauthor=true&cauthor_uid=26237428)^49,50,51^, [van der Stoep N](https://www.ncbi.nlm.nih.gov/pubmed/?term=van%20der%20Stoep%20N%5BAuthor%5D&cauthor=true&cauthor_uid=26237428)^52^, [van Doorn R](https://www.ncbi.nlm.nih.gov/pubmed/?term=van%20Doorn%20R%5BAuthor%5D&cauthor=true&cauthor_uid=26237428)^28^, [Whiteman DC](https://www.ncbi.nlm.nih.gov/pubmed/?term=Whiteman%20DC%5BAuthor%5D&cauthor=true&cauthor_uid=26237428)^53^, [Craig JE](https://www.ncbi.nlm.nih.gov/pubmed/?term=Craig%20JE%5BAuthor%5D&cauthor=true&cauthor_uid=26237428)^54^, [Schadendorf D](https://www.ncbi.nlm.nih.gov/pubmed/?term=Schadendorf%20D%5BAuthor%5D&cauthor=true&cauthor_uid=26237428)^55,56^, [Simms LA](https://www.ncbi.nlm.nih.gov/pubmed/?term=Simms%20LA%5BAuthor%5D&cauthor=true&cauthor_uid=26237428)^47^, [Burdon KP](https://www.ncbi.nlm.nih.gov/pubmed/?term=Burdon%20KP%5BAuthor%5D&cauthor=true&cauthor_uid=26237428)^57^, [Nyholt DR](https://www.ncbi.nlm.nih.gov/pubmed/?term=Nyholt%20DR%5BAuthor%5D&cauthor=true&cauthor_uid=26237428)^40,58^, [Pooley KA](https://www.ncbi.nlm.nih.gov/pubmed/?term=Pooley%20KA%5BAuthor%5D&cauthor=true&cauthor_uid=26237428)^10^, [Orr N](https://www.ncbi.nlm.nih.gov/pubmed/?term=Orr%20N%5BAuthor%5D&cauthor=true&cauthor_uid=26237428)^59^, [Stratigos AJ](https://www.ncbi.nlm.nih.gov/pubmed/?term=Stratigos%20AJ%5BAuthor%5D&cauthor=true&cauthor_uid=26237428)^14^, [Cust AE](https://www.ncbi.nlm.nih.gov/pubmed/?term=Cust%20AE%5BAuthor%5D&cauthor=true&cauthor_uid=26237428)^60^, [Ward SV](https://www.ncbi.nlm.nih.gov/pubmed/?term=Ward%20SV%5BAuthor%5D&cauthor=true&cauthor_uid=26237428)^7^, [Hayward NK](https://www.ncbi.nlm.nih.gov/pubmed/?term=Hayward%20NK%5BAuthor%5D&cauthor=true&cauthor_uid=26237428)^61^, [Han J](https://www.ncbi.nlm.nih.gov/pubmed/?term=Han%20J%5BAuthor%5D&cauthor=true&cauthor_uid=26237428)^46,47^, [Schulze HJ](https://www.ncbi.nlm.nih.gov/pubmed/?term=Schulze%20HJ%5BAuthor%5D&cauthor=true&cauthor_uid=26237428)^62^, [Dunning AM](https://www.ncbi.nlm.nih.gov/pubmed/?term=Dunning%20AM%5BAuthor%5D&cauthor=true&cauthor_uid=26237428)^11^, [Bishop JA](https://www.ncbi.nlm.nih.gov/pubmed/?term=Bishop%20JA%5BAuthor%5D&cauthor=true&cauthor_uid=26237428)^2^, [Demenais F](https://www.ncbi.nlm.nih.gov/pubmed/?term=Demenais%20F%5BAuthor%5D&cauthor=true&cauthor_uid=26237428)^4,5#^, [Amos CI](https://www.ncbi.nlm.nih.gov/pubmed/?term=Amos%20CI%5BAuthor%5D&cauthor=true&cauthor_uid=26237428)^63#^, [MacGregor S](https://www.ncbi.nlm.nih.gov/pubmed/?term=MacGregor%20S%5BAuthor%5D&cauthor=true&cauthor_uid=26237428)^1*^, [Iles MM](https://www.ncbi.nlm.nih.gov/pubmed/?term=Iles%20MM%5BAuthor%5D&cauthor=true&cauthor_uid=26237428)^2*^.

^1^Statistical Genetics, QIMR Berghofer Medical Research Institute, Brisbane, Queensland, Australia.

^2^Section of Epidemiology and Biostatistics, Leeds Institute of Cancer and Pathology, University of Leeds, Leeds, UK.

^3^Department of Surgical Oncology, University of Texas MD Anderson Cancer Center, Houston, Texas, USA.

^4^INSERM, UMR 946, Genetic Variation and Human Diseases Unit, Paris, France.

^5^Institut Universitaire d'Hématologie, Université Paris Diderot, Sorbonne Paris Cité, Paris, France.

^6^Genetic Epidemiology, QIMR Berghofer Medical Research Institute, Brisbane, Queensland, Australia.

^7^Centre for Genetic Origins of Health and Disease, Faculty of Medicine, Dentistry and Health Sciences, University of Western Australia, Perth, Western Australia, Australia.

^8^Department of Epidemiology and Biostatistics, Key Laboratory of Cancer Prevention and Therapy, Tianjin, National Clinical Research Center of Cancer, Tianjin Medical University Cancer Institute and Hospital, Tianjin, China.

^9^Division of Molecular Genetic Epidemiology, German Cancer Research Center, Heidelberg, Germany.

^10^Centre for Cancer Genetic Epidemiology, Department of Public Health and Primary Care, University of Cambridge, Cambridge, UK.

^11^Centre for Cancer Genetic Epidemiology, Department of Oncology, University of Cambridge, Cambridge, UK.

^12^Division of Genetics and Epidemiology, The Institute of Cancer Research, London, UK.

^13^Division of Breast Cancer Research, The Institute of Cancer Research, London, UK.

^14^Department of Dermatology, University of Athens School of Medicine, Andreas Sygros Hospital, Athens, Greece.

^15^Centre for Cancer Biomarkers (CCBIO), Department of Clinical Medicine, University of Bergen, Bergen, Norway.

^16^Department of Pathology, Haukeland University Hospital, Bergen, Norway.

^17^Department of Pathology, Molecular Pathology, Oslo University Hospital, Rikshospitalet, Oslo, Norway.

^18^Assistance Publique-Hôpitaux de Paris, Hôpital Cochin, Service de Dermatologie, Université Paris Descartes, Paris, France.

^19^Department of Dermatology, Sheba Medical Center, Tel Hashomer, Sackler Faculty of Medicine, Tel Aviv, Israel.

^20^Oncogenetics Unit, Sheba Medical Center, Tel Hashomer, Sackler Faculty of Medicine, Tel Aviv University, Tel Aviv, Israel.

^21^Department of Internal Medicine and Medical Specialties, University of Genoa, Genoa, Italy.

^22^Laboratory of Genetics of Rare Cancers, Istituto di Ricovero e Cura a Carattere Scientifico Azienda Ospedaliera Universitaria (IRCCS AOU) San Martino l'Istituto Scientifico Tumori Istituto Nazionale per la Ricerca sul Cancro, Genoa, Italy.

^23^Division of Cancer Epidemiology and Genetics, National Cancer Institute, US National Institutes of Health, Bethesda, Maryland, USA.

^24^International Hereditary Cancer Center, Pomeranian Medical University, Szczecin, Poland.

^25^Department of Pathology and Laboratory Medicine, Perelman School of Medicine at the University of Pennsylvania, Philadelphia, Pennsylvania, USA.

^26^Université Paris 13, Equipe de Recherche en Epidémiologie Nutritionnelle (EREN), Centre de Recherche en Epidémiologie et Statistiques, INSERM U1153, Institut National de la Recherche Agronomique (INRA) U1125, Conservatoire National des Arts et Métiers, Communauté d'Université Sorbonne Paris Cité, Bobigny, France.

^27^Inherited Disease Research Branch, National Human Genome Research Institute, US National Institutes of Health, Baltimore, Maryland, USA.

^28^Department of Dermatology, Leiden University Medical Center, Leiden, the Netherlands.

^29^Department of Oncology-Pathology, Karolinska Institutet, Karolinska University Hospital, Stockholm, Sweden.

^30^Department of Dermatology, Oslo University Hospital, Rikshospitalet, Oslo, Norway.

^31^Department of Surgical Oncology, Institute of Oncology Ljubljana, Ljubljana, Slovenia.

^32^Department of Surgery, Clinical Sciences, Lund University, Lund, Sweden.

^33^Department of Cancer Epidemiology, H. Lee Moffitt Cancer Center and Research Institute, Tampa, Florida, USA.

^34^Department of Genetics, University of Texas MD Anderson Cancer Center, Houston, Texas, USA.

^35^Department of Medical Genetics, University of Glasgow, Glasgow, UK.

^36^McGill University and Génome Québec Innovation Centre, Montreal, Quebec, Canada.

^37^Department of Public Health, University of Glasgow, Glasgow, UK.

^38^Centre for Cancer Research, University of Sydney at Westmead, Millennium Institute for Medical Research and Melanoma Institute Australia, Sydney, New South Wales, Australia.

^39^Gade Laboratory for Pathology, Department of Clinical Medicine, University of Bergen, Bergen, Norway.

^40^Molecular Biology, the University of Queensland, Brisbane, Australia.

^41^Department of Molecular Diagnostics, Institute of Oncology Ljubljana, Ljubljana, Slovenia.

^42^Department of Oncology/Pathology, Clinical Sciences, Lund University, Lund, Sweden.

^43^Department of Cancer Epidemiology, Clinical Sciences, Lund University, Lund, Sweden.

^44^Melanoma Unit, Departments of Dermatology, Biochemistry and Molecular Genetics, Hospital Clinic, Institut d'Investigacions Biomèdica August Pi Suñe, Universitat de Barcelona, Barcelona, Spain.

^45^Centro de Investigación Biomédica en Red (CIBER) de Enfermedades Raras, Instituto de Salud Carlos III, Barcelona, Spain.

^46^Department of Epidemiology, Richard M. Fairbanks School of Public Health, Indiana University, Indianapolis, Indiana, USA.

^47^Melvin and Bren Simon Cancer Center, Indiana University, Indianapolis, Indiana, USA.

^48^Department of Dermatology, Warren Alpert Medical School of Brown University, Providence, Rhode Island, USA.

^49^Inflammatory Bowel Diseases, QIMR Berghofer Medical Research Institute, Brisbane, Queensland, Australia.

^50^Department of Gastroenterology and Hepatology, Royal Brisbane and Women's Hospital, Brisbane, Queensland, Australia.

^51^University of Queensland School of Medicine, Herston Campus, Brisbane, Queensland, Australia.

^52^Department of Clinical Genetics, Center of Human and Clinical Genetics, Leiden University Medical Center, Leiden, the Netherlands.

^53^Cancer Control Group, QIMR Berghofer Medical Research Institute, Brisbane, Queensland, Australia.

^54^Department of Ophthalmology, Flinders University, Adelaide, South Australia, Australia.

^55^Department of Dermatology, University Hospital Essen, Essen, Germany.

^56^German Consortium for Translational Cancer Research (DKTK), Heidelberg, Germany.

^57^Menzies Institute for Medical Research, University of Tasmania, Hobart, Tasmania, Australia.

^58^Institute of Health and Biomedical Innovation, Queensland University of Technology, Brisbane, Queensland, Australia.

^59^Breakthrough Breast Cancer Research Centre, The Institute of Cancer Research, London, UK.

^60^Cancer Epidemiology and Services Research, Sydney School of Public Health, University of Sydney, Sydney, New South Wales, Australia.

^61^Oncogenomics, QIMR Berghofer Medical Research Institute, Brisbane, Queensland, Australia.

^62^Department of Dermatology, Fachklinik Hornheide, Institute for Tumors of the Skin at the University of Münster, Münster, Germany.

^63^Department of Community and Family Medicine, Geisel School of Medicine, Dartmouth College, Hanover, New Hampshire, USA.

* Supervised equally. ^#^ Contributed equally.

Studies and funding acknowledgements of the MMAC:

GenoMEL

The GenoMEL study (<http://www.genomel.org/>) was funded by the European Commission under the 6^th^ Framework Programme (contract no. LSHC-CT-2006-018702), by Cancer Research UK Programme Awards (C588/A4994 and C588/A10589), by a Cancer Research UK Project Grant (C8216/A6129) and by a grant from the US National Institutes of Health (NIH; CA83115). This research was also supported by the intramural Research Program of the NIH, National Cancer Institute (NCI), Division of Cancer Epidemiology and Genetics.

This study makes use of data generated by the Wellcome Trust Case Control Consortium (<http://www.wtccc.org.uk/>). A full list of the investigators who contributed to the generation of the data is available from their website (see URLs). Funding for the project was provided by the Wellcome Trust under award 076113.

Genotyping for the CIDRUK samples were provided by the Center for Inherited Disease Research (CIDR). CIDR is fully funded through a federal contract from the National Institutes of Health to The Johns Hopkins University, contract number HHSN268201200008I.

Funding specific to particular centers is given below:

Stockholm: Swedish Cancer Society, Karolinska Institutet Research Funds, Radiumhemmet Research Funds, Stockholm County Council Research Funding (ALF).

Lund: Funding to be acknowledged; Swedish Cancer Society, Gunnar Nilsson Foundation, and European Research Council Advanced Grant (ERC-2011–294576).

Genoa: Italian Ministry of Education, University and Research PRIN 2008, IMI and Mara Naum foundation. Italian association for cancer research (AIRC) IG 2014 (15460) to PG; IRCCS AOU San Martino-IST Istituto Nazionale per la Ricerca sul Cancro, 5% per la ricerca corrente, to PG and GBS.

Leiden: Grant provided by European Biobanking and Biomolecular Resources Research Infrastructure (BBMRI) −Netherlands hub (CO18).

Spain: The research at the Melanoma Unit in Barcelona is or was partially funded by Grants from Fondo de Investigaciones Sanitarias P.I. 09/01393 & 12/00840, Spain; by the CIBER de Enfermedades Raras of the Instituto de Salud Carlos III, Spain; by the AGAUR 2009 SGR 1337 and AGAUR 2014_SGR_603 of the Catalan Government, Spain; by a grant from “Fundació La Marató de TV3, 201331-30”, Catalonia, Spain; by the European Commission under the 6th Framework Programme, Contract nº: LSHC-CT-2006-018702 (GenoMEL) and by the National Cancer Institute (NCI) of the US National Institute of Health (NIH) (CA83115).

Norway: Grants from the Comprehensive Cancer Center, Oslo University Hospital (SE0728) and the Norwegian Cancer Society (71512-PR-2006-0356).

AMFS

The AMFS was supported by the National Health and Medical Research Council of Australia (NHMRC) (project grants 566946, 107359, 211172 and program grant number 402761 to GJM and RFK); the Cancer Council New South Wales (project grant 77/00, 06/10), the Cancer Council Victoria and the Cancer Council Queensland (project grant 371); and the US National Institutes of Health (NIH RO1 grant CA-83115-01A2 and 2R01CA083115-11A1 to the international Melanoma Genetics Consortium - GenoMEL). Anne E. Cust is supported by fellowships from the Cancer Institute NSW and the NHMRC. We gratefully acknowledge all of the participants, and the work and dedication of the research coordinators, interviewers, examiners and data management staff.

WAMHS

The WAMHS gratefully acknowledges all study participants for their time and contributions, and the Western Australian DNA Bank and the Ark at The University of Western Australia for biospecimen and bioinformatics related support. The Western Australian Cancer Registry, the WAMHS study team and the WAMHS Management Committee are also gratefully acknowledged for their assistance, as well as the Scott Kirkbride Melanoma Research Centre for funding received to establish the WAMHS resource and related salaries and PhD stipends. The Cancer Council Western Australia is also acknowledged for current salary support for Sarah Ward (Capacity Building and Collaboration grant).

Genotyping services were provided by the Center for Inherited Disease Research (CIDR). CIDR is fully funded through a federal contract from the National Institutes of Health to The Johns Hopkins University, contract number HHSN268201200008I’

Q-MEGA cases and QTWINs controls (used in Q-MEGA_610k set)

Acknowledgement/grants: Q-MEGA and QTWIN thanks A. Baxter, M. de Nooyer, I. Gardner, D. Statham, B. Haddon, M.J. Wright, J. Palmer, J. Symmons, B. Castellano, L. Bardsley, S. Smith, D. Smyth, L. Wallace, M.J. Campbell, A. Caracella, M. Kvaskoff, O. Zheng, B. Chapman and H. Beeby for their input in project management, sample processing and database development. We are grateful to the many research assistants and interviewers for assistance with the studies contributing to the QMEGA and QTWIN collections

The Q-MEGA/QTWIN study was supported by the Melanoma Research Alliance, the NIH NCI (CA88363, CA83115, CA122838, CA87969, CA055075, CA100264, CA133996 and CA49449), the National Health and Medical Research Council of Australia (NHMRC) (200071, 241944, 339462, 380385, 389927,389875, 389891, 389892,389938, 443036, 442915, 442981, 496610, 496675, 496739, 552485, 552498), the Cancer Councils New South Wales, Victoria and Queensland, the Cancer Institute New South Wales, the Cooperative Research Centre for Discovery of Genes for Common Human Diseases (CRC), Cerylid Biosciences (Melbourne), the Australian Cancer Research Foundation, The Wellcome Trust (WT084766/Z/08/Z) and donations from Neville and Shirley Hawkins. Stuart MacGregor acknowledges fellowship support from the Australian National Health and Medical Research Council and from the Australian Research Council.

## Pancreatic Cancer Case-Control Consortium (PanC4)

The PANC4 GWAS was supported by RO1 CA154823. The IARC/Central Europe study was supported by a grant from the US NCI at the NIH (R03 CA123546-02) and grants from the Ministry of Health of the Czech Republic (NR 9029-4/2006, NR9422-3, NR9998-3, and MH CZ-DRO-MMCI 00209805). The work at Johns Hopkins University was supported by the NCI Grants P50CA062924. The Mayo Clinic Biospecimen Resource for Pancreas Research study is supported by the Mayo Clinic SPORE in Pancreatic Cancer (P50 CA102701). The Memorial Sloan Kettering Cancer Center Pancreatic Tumor Registry is supported by P30CA008748, the Geoffrey Beene Foundation, the Arnold and Arlene Goldstein Family, Foundation, and the Society of MSKCC. The Queensland Pancreatic Cancer Study was supported by a grant from the National Health and Medical Research Council of Australia (NHMRC; Grant number 442302).). The UCSF pancreas study was supported by NIH-NCI grants (R01CA1009767, R01CA109767-S1, and R0CA059706) and the Joan Rombauer Pancreatic Cancer Fund. The Yale (CT) pancreas cancer study is supported by NCI at the U.S. NIH, grant 5R01CA098870.

## The Prostate Cancer Association Group to Investigate Cancer Associated Alterations in the Genome (PRACTICAL) Consortium

**PIs from the PRACTICAL (**[**http://practical.icr.ac.uk/**](http://practical.icr.ac.uk/)**), CRUK, BPC3, CAPS, PEGASUS consortia:**

Rosalind A. Eeles^1,2^, Christopher A. Haiman^3^, Zsofia Kote-Jarai^1^, Fredrick R. Schumacher^4,5^, Sara Benlloch^6,1^, Ali Amin Al Olama^6,7^, Kenneth R. Muir^8^, Sonja I. Berndt^9^, David V. Conti^3^, Fredrik Wiklund^10^, Stephen Chanock^9^, Ying Wang^11^, Catherine M. Tangen^12^, Jyotsna Batra^13,14^, Judith A. Clements^13,14^, APCB BioResource (Australian Prostate Cancer BioResource)^15,14^, Henrik Grönberg^10^, Nora Pashayan^16,17^, Johanna Schleutker^18,19^, Demetrius Albanes^9^, Stephanie J. Weinstein^9^, Alicja Wolk^20^, Catharine M. L. West^21^, Lorelei A. Mucci^22^, Géraldine Cancel-Tassin^23,24^, Stella Koutros^9^, Karina Dalsgaard Sørensen^25,26^, Eli Marie Grindedal^27^, David E. Neal^28,29,30^, Freddie C. Hamdy^31,32^, Jenny L. Donovan^33^, Ruth C. Travis^34^, Robert J. Hamilton^35,36^, Sue Ann Ingles^37^, Barry S. Rosenstein^38^, Yong-Jie Lu^39^, Graham G. Giles^40,41,42^, Robert J. MacInnis^40,41^, Adam S. Kibel^43^, Ana Vega^44,45,46^, Manolis Kogevinas^47,48,49,50^, Kathryn L. Penney^51^, Jong Y. Park^52^, Janet L. Stanford^53,54^, Cezary Cybulski^55^, Børge G. Nordestgaard^56,57^, Sune F. Nielsen^56,57^, Hermann Brenner^58,59,60^, Christiane Maier^61^, Jeri Kim^62^, Esther M. John^63^, Manuel R. Teixeira^64,65,66^, Susan L. Neuhausen^67^, Kim De Ruyck^68^, Azad Razack^69^, Lisa F. Newcomb^53,70^, Davor Lessel^71^, Radka Kaneva^72^, Nawaid Usmani^73,74^, Frank Claessens^75^, Paul A. Townsend^76,77^, Jose Esteban Castelao^78^, Monique J. Roobol^79^, Florence Menegaux^80^, Kay-Tee Khaw^81^, Lisa Cannon-Albright^82,83^, Hardev Pandha^77^, Stephen N. Thibodeau^84^, David J. Hunter^85^, Peter Kraft^86^, William J. Blot^87,88^, Elio Riboli^89^

^1^The Institute of Cancer Research, London, SM2 5NG, UK
^2^Royal Marsden NHS Foundation Trust, London, SW3 6JJ, UK
^3^Center for Genetic Epidemiology, Department of Preventive Medicine, Keck School of Medicine, University of Southern California/Norris Comprehensive Cancer Center, Los Angeles, CA 90015, USA
^4^Department of Population and Quantitative Health Sciences, Case Western Reserve University, Cleveland, OH 44106-7219, USA
^5^Seidman Cancer Center, University Hospitals, Cleveland, OH 44106, USA.
^6^Centre for Cancer Genetic Epidemiology, Department of Public Health and Primary Care, University of Cambridge, Strangeways Research Laboratory, Cambridge CB1 8RN, UK
^7^University of Cambridge, Department of Clinical Neurosciences, Stroke Research Group, R3, Box 83, Cambridge Biomedical Campus, Cambridge CB2 0QQ, UK
^8^Division of Population Health, Health Services Research and Primary Care, University of Manchester, Oxford Road, Manchester, M13 9PL, UK
^9^Division of Cancer Epidemiology and Genetics, National Cancer Institute, NIH, Bethesda, Maryland, 20892, USA
^10^Department of Medical Epidemiology and Biostatistics, Karolinska Institute, SE-171 77 Stockholm, Sweden
^11^Department of Population Science, American Cancer Society, 250 Williams Street, Atlanta, GA 30303, USA
^12^SWOG Statistical Center, Fred Hutchinson Cancer Research Center, Seattle, WA 98109, USA
^13^Australian Prostate Cancer Research Centre-Qld, Institute of Health and Biomedical Innovation and School of Biomedical Sciences, Queensland University of Technology, Brisbane QLD 4059, Australia
^14^Translational Research Institute, Brisbane, Queensland 4102, Australia
^15^Australian Prostate Cancer Research Centre-Qld, Queensland University of Technology, Brisbane; Prostate Cancer Research Program, Monash University, Melbourne; Dame Roma Mitchell Cancer Centre, University of Adelaide, Adelaide; Chris O'Brien Lifehouse and The Kinghorn Cancer Centre, Sydney, Australia
^16^Department of Applied Health Research, University College London, London, WC1E 7HB, UK
^17^Centre for Cancer Genetic Epidemiology, Department of Oncology, University of Cambridge, Strangeways Laboratory, Worts Causeway, Cambridge, CB1 8RN, UK
^18^Institute of Biomedicine, University of Turku, Finland
^19^Department of Medical Genetics, Genomics, Laboratory Division, Turku University Hospital, PO Box 52, 20521 Turku, Finland
^20^Department of Surgical Sciences, Uppsala University, 75185 Uppsala, Sweden
^21^Division of Cancer Sciences, University of Manchester, Manchester Academic Health Science Centre, Radiotherapy Related Research, The Christie Hospital NHS Foundation Trust, Manchester, M13 9PL UK
^22^Department of Epidemiology, Harvard T. H. Chan School of Public Health, Boston, MA 02115, USA
^23^CeRePP, Tenon Hospital, F-75020 Paris, France.
^24^Sorbonne Universite, GRC n°5 , AP-HP, Tenon Hospital, 4 rue de la Chine, F-75020 Paris, France
^25^Department of Molecular Medicine, Aarhus University Hospital, Palle Juul-Jensen Boulevard 99, 8200 Aarhus N, Denmark
^26^Department of Clinical Medicine, Aarhus University, DK-8200 Aarhus N
^27^Department of Medical Genetics, Oslo University Hospital, 0424 Oslo, Norway
^28^Nuffield Department of Surgical Sciences, University of Oxford, Room 6603, Level 6, John Radcliffe Hospital, Headley Way, Headington, Oxford, OX3 9DU, UK
^29^University of Cambridge, Department of Oncology, Box 279, Addenbrooke's Hospital, Hills Road, Cambridge CB2 0QQ, UK
^30^Cancer Research UK, Cambridge Research Institute, Li Ka Shing Centre, Cambridge, CB2 0RE, UK
^31^Nuffield Department of Surgical Sciences, University of Oxford, Oxford, OX1 2JD, UK
^32^Faculty of Medical Science, University of Oxford, John Radcliffe Hospital, Oxford, UK
^33^Population Health Sciences, Bristol Medical School, University of Bristol, BS8 2PS, UK
^34^Cancer Epidemiology Unit, Nuffield Department of Population Health, University of Oxford, Oxford, OX3 7LF, UK
^35^Dept. of Surgical Oncology, Princess Margaret Cancer Centre, Toronto ON M5G 2M9, Canada
^36^Dept. of Surgery (Urology), University of Toronto, Canada
^37^Department of Preventive Medicine, Keck School of Medicine, University of Southern California/Norris Comprehensive Cancer Center, Los Angeles, CA 90015, USA
^38^Department of Radiation Oncology and Department of Genetics and Genomic Sciences, Box 1236, Icahn School of Medicine at Mount Sinai, One Gustave L. Levy Place, New York, NY 10029, USA
^39^Centre for Cancer Biomarker and Biotherapeutics, Barts Cancer Institute, Queen Mary University of London, John Vane Science Centre, Charterhouse Square, London, EC1M 6BQ, UK
^40^Cancer Epidemiology Division, Cancer Council Victoria, 615 St Kilda Road, Melbourne, VIC 3004, Australia
^41^Centre for Epidemiology and Biostatistics, Melbourne School of Population and Global Health, The University of Melbourne, Grattan Street, Parkville, VIC 3010, Australia
^42^Precision Medicine, School of Clinical Sciences at Monash Health, Monash University, Clayton, Victoria 3168, Australia
^43^Division of Urologic Surgery, Brigham and Womens Hospital, 75 Francis Street, Boston, MA 02115, USA
^44^Fundación Pública Galega Medicina Xenómica, Santiago de Compostela, 15706, Spain.
^45^Instituto de Investigación Sanitaria de Santiago de Compostela, Santiago de Compostela, 15706, Spain.
^46^Centro de Investigación en Red de Enfermedades Raras (CIBERER), Spain
^47^ISGlobal, Barcelona, Spain
^48^IMIM (Hospital del Mar Medical Research Institute), Barcelona, Spain
^49^Universitat Pompeu Fabra (UPF), Barcelona, Spain
^50^CIBER Epidemiología y Salud Pública (CIBERESP), 28029 Madrid, Spain
^51^Channing Division of Network Medicine, Department of Medicine, Brigham and Women's Hospital/Harvard Medical School, Boston, MA 02115, USA
^52^Department of Cancer Epidemiology, Moffitt Cancer Center, 12902 Magnolia Drive, Tampa, FL 33612, USA
^53^Division of Public Health Sciences, Fred Hutchinson Cancer Research Center, Seattle, Washington, 98109-1024, USA
^54^Department of Epidemiology, School of Public Health, University of Washington, Seattle, Washington 98195, USA
^55^International Hereditary Cancer Center, Department of Genetics and Pathology, Pomeranian Medical University, 70-115 Szczecin, Poland
^56^Faculty of Health and Medical Sciences, University of Copenhagen, 2200 Copenhagen, Denmark
^57^Department of Clinical Biochemistry, Herlev and Gentofte Hospital, Copenhagen University Hospital, Herlev, 2200 Copenhagen, Denmark
^58^Division of Clinical Epidemiology and Aging Research, German Cancer Research Center (DKFZ), D-69120, Heidelberg, Germany
^59^German Cancer Consortium (DKTK), German Cancer Research Center (DKFZ), D-69120 Heidelberg, Germany
^60^Division of Preventive Oncology, German Cancer Research Center (DKFZ) and National Center for Tumor Diseases (NCT), Im Neuenheimer Feld 460, 69120 Heidelberg, Germany
^61^Humangenetik Tuebingen, Paul-Ehrlich-Str 23, D-72076 Tuebingen, Germany
^62^The University of Texas M. D. Anderson Cancer Center, Department of Genitourinary Medical Oncology, 1515 Holcombe Blvd., Houston, TX 77030, USA
^63^Departments of Epidemiology & Population Health and of Medicine, Division of Oncology, Stanford Cancer Institute, Stanford University School of Medicine, Stanford, CA 94304 USA
^64^Department of Laboratory Genetics, Portuguese Oncology Institute of Porto (IPO Porto) / Porto Comprehensive Cancer Center, Porto, Portugal
^65^Cancer Genetics Group, IPO Porto Research Center (CI-IPOP) / RISE@CI-IPOP (Health Research Network), Portuguese Oncology Institute of Porto (IPO Porto) / Porto Comprehensive Cancer Center, Porto, Portugal
^66^School of Medicine and Biomedical Sciences (ICBAS), University of Porto, Porto, Portugal
^67^Department of Population Sciences, Beckman Research Institute of the City of Hope, 1500 East Duarte Road, Duarte, CA 91010
^68^Ghent University, Faculty of Medicine and Health Sciences, Basic Medical Sciences, Proeftuinstraat 86, B-9000 Gent
^69^Department of Surgery, Faculty of Medicine, University of Malaya, 50603 Kuala Lumpur, Malaysia
^70^Department of Urology, University of Washington, 1959 NE Pacific Street, Box 356510, Seattle, WA 98195, USA
^71^Institute of Human Genetics, University Medical Center Hamburg-Eppendorf, D-20246 Hamburg, Germany
^72^Molecular Medicine Center, Department of Medical Chemistry and Biochemistry, Medical University of Sofia, Sofia, 2 Zdrave Str., 1431 Sofia, Bulgaria
^73^Department of Oncology, Cross Cancer Institute, University of Alberta, 11560 University Avenue, Edmonton, Alberta, Canada T6G 1Z2
^74^Division of Radiation Oncology, Cross Cancer Institute, 11560 University Avenue, Edmonton, Alberta, Canada T6G 1Z2
^75^Molecular Endocrinology Laboratory, Department of Cellular and Molecular Medicine, KU Leuven, BE-3000, Belgium
^76^Division of Cancer Sciences, Manchester Cancer Research Centre, Faculty of Biology, Medicine and Health, Manchester Academic Health Science Centre, NIHR Manchester Biomedical Research Centre, Health Innovation Manchester, Univeristy of Manchester, M13 9WL
^77^The University of Surrey, Guildford, Surrey, GU2 7XH, UK
^78^Genetic Oncology Unit, CHUVI Hospital, Complexo Hospitalario Universitario de Vigo, Instituto de Investigación Biomédica Galicia Sur (IISGS), 36204, Vigo (Pontevedra), Spain
^79^Department of Urology, Erasmus University Medical Center, Cancer Institute, 3015 GD Rotterdam, The Netherlands
^80^"Exposome and Heredity", CESP (UMR 1018), Faculté de Médecine, Université Paris-Saclay, Inserm, Gustave Roussy, Villejuif
^81^Clinical Gerontology Unit, University of Cambridge, Cambridge, CB2 2QQ, UK
^82^Division of Epidemiology, Department of Internal Medicine, University of Utah School of Medicine, Salt Lake City, Utah 84132, USA
^83^George E. Wahlen Department of Veterans Affairs Medical Center, Salt Lake City, Utah 84148, USA
^84^Department of Laboratory Medicine and Pathology, Mayo Clinic, Rochester, MN 55905, USA
^85^Nuffield Department of Population Health, University of Oxford, United Kingdom
^86^Program in Genetic Epidemiology and Statistical Genetics, Department of Epidemiology, Harvard School of Public Health, Boston, MA, USA
^87^Division of Epidemiology, Department of Medicine, Vanderbilt University Medical Center, 2525 West End Avenue, Suite 800, Nashville, TN 37232 USA.
^88^International Epidemiology Institute, Rockville, MD 20850, USA
^89^Department of Epidemiology and Biostatistics, School of Public Health, Imperial College London, SW7 2AZ, UK

Acknowledgements

CRUK and PRACTICAL consortium

This work was supported by the Canadian Institutes of Health Research, European Commission's Seventh Framework Programme grant agreement n° 223175 (HEALTH-F2-2009-223175), Cancer Research UK Grants C5047/A7357, C1287/A10118, C1287/A16563, C5047/A3354, C5047/A10692, C16913/A6135, and The National Institute of Health (NIH) Cancer Post-Cancer GWAS initiative grant: No. 1 U19 CA 148537-01 (the GAME-ON initiative).

We would also like to thank the following for funding support: The Institute of Cancer Research and The Everyman Campaign, The Prostate Cancer Research Foundation, Prostate Research Campaign UK (now PCUK), The Orchid Cancer Appeal, Rosetrees Trust, The National Cancer Research Network UK, The National Cancer Research Institute (NCRI) UK. We are grateful for support of NIHR funding to the NIHR Biomedical Research Centre at The Institute of Cancer Research, The Royal Marsden NHS Foundation Trust, and Manchester NIHR Biomedical Research Centre. The Prostate Cancer Program of Cancer Council Victoria also acknowledge grant support from The National Health and Medical Research Council, Australia (126402, 209057, 251533, , 396414, 450104, 504700, 504702, 504715, 623204, 940394, 614296,), VicHealth, Cancer Council Victoria, The Prostate Cancer Foundation of Australia, The Whitten Foundation, PricewaterhouseCoopers, and Tattersall’s. EAO, DMK, and EMK acknowledge the Intramural Program of the National Human Genome Research Institute for their support.

Genotyping of the OncoArray was funded by the US National Institutes of Health (NIH) [U19 CA 148537 for ELucidating Loci Involved in Prostate cancer SuscEptibility (ELLIPSE) project and X01HG007492 to the Center for Inherited Disease Research (CIDR) under contract number HHSN268201200008I]. Additional analytic support was provided by NIH NCI U01 CA188392 (PI: Schumacher).

Research reported in this publication also received support from the National Cancer Institute of the National Institutes of Health under Award Numbers U10 CA37429 (CD Blanke), and UM1 CA182883 (CM Tangen/IM Thompson). The content is solely the responsibility of the authors and does not necessarily represent the official views of the National Institutes of Health.

Funding for the iCOGS infrastructure came from: the European Community's Seventh Framework Programme under grant agreement n° 223175 (HEALTH-F2-2009-223175) (COGS), Cancer Research UK (C1287/A10118, C1287/A 10710, C12292/A11174, C1281/A12014, C5047/A8384, C5047/A15007, C5047/A10692, C8197/A16565), the National Institutes of Health (CA128978) and Post-Cancer GWAS initiative (1U19 CA148537, 1U19 CA148065 and 1U19 CA148112 - the GAME-ON initiative), the Department of Defence (W81XWH-10-1-0341), the Canadian Institutes of Health Research (CIHR) for the CIHR Team in Familial Risks of Breast Cancer, Komen Foundation for the Cure, the Breast Cancer Research Foundation, and the Ovarian Cancer Research Fund.

BPC3

The BPC3 was supported by the U.S. National Institutes of Health, National Cancer Institute (cooperative agreements U01-CA98233 to D.J.H., U01-CA98710 to S.M.G., U01-CA98216 to E.R., and U01-CA98758 to B.E.H., and Intramural Research Program of NIH/National Cancer Institute, Division of Cancer Epidemiology and Genetics).

CAPS

CAPS GWAS study was supported by the Cancer Risk Prediction Center (CRisP; www.crispcenter.org), a Linneus Centre (Contract ID 70867902) financed by the Swedish Research Council, (grant no K2010-70X-20430-04-3), the Swedish Cancer Foundation (grant no 09-0677), the Hedlund Foundation, the Soederberg Foundation, the Enqvist Foundation, ALF funds from the Stockholm County Council. Stiftelsen Johanna Hagstrand och Sigfrid Linner's Minne, Karlsson's Fund for urological and surgical research.

PEGASUS

PEGASUS was supported by the Intramural Research Program, Division of Cancer Epidemiology and Genetics, National Cancer Institute, National Institutes of Health.

# References

1. Burgess S, Thompson SG. Bias in causal estimates from Mendelian randomization studies with weak instruments. Stat Med [Internet]. 2011 May 20;30(11):1312–23. Available from: http://www.ncbi.nlm.nih.gov/pubmed/21432888

2. Burgess S, Davies NM, Thompson SG. Bias due to participant overlap in two‐sample Mendelian randomization. Genet Epidemiol [Internet]. 2016 Nov 1 [cited 2022 Feb 25];40(7):597. Available from: /pmc/articles/PMC5082560/

3. Auton A, Abecasis GR, Altshuler DM, Durbin RM, Bentley DR, Chakravarti A, et al. A global reference for human genetic variation [Internet]. Vol. 526, Nature. Nature Publishing Group; 2015 [cited 2020 Dec 23]. p. 68–74. Available from: https://www.nature.com/articles/nature15393

4. Shin S-Y, Fauman EB, Petersen A-K, Krumsiek J, Santos R, Huang J, et al. An atlas of genetic influences on human blood metabolites. Nat Genet [Internet]. 2014 Jun 11 [cited 2019 Mar 2];46(6):543–50. Available from: http://www.ncbi.nlm.nih.gov/pubmed/24816252

5. Dorajoo R, Sun Y, Han Y, Ke T, Burger A, Chang X, et al. A genome-wide association study of n-3 and n-6 plasma fatty acids in a Singaporean Chinese population. Genes Nutr [Internet]. 2015 Nov 19 [cited 2019 Mar 2];10(6):53. Available from: http://link.springer.com/10.1007/s12263-015-0502-2

6. Kettunen J, Demirkan A, Würtz P, Draisma HHM, Haller T, Rawal R, et al. Genome-wide study for circulating metabolites identifies 62 loci and reveals novel systemic effects of LPA. Nat Commun [Internet]. 2016 Jan 23 [cited 2016 Mar 24];7(1):11122. Available from: http://www.nature.com/articles/ncomms11122

7. Tintle NL, Pottala J V., Lacey S, Ramachandran V, Westra J, Rogers A, et al. A genome-wide association study of saturated, mono- and polyunsaturated red blood cell fatty acids in the Framingham Heart Offspring Study. Prostaglandins Leukot Essent Fat Acids [Internet]. 2015 Mar [cited 2019 Mar 2];94:65–72. Available from: https://linkinghub.elsevier.com/retrieve/pii/S0952327814001987

8. Hemani G, Zheng J, Elsworth B, Wade KH, Haberland V, Baird D, et al. The MR-Base platform supports systematic causal inference across the human phenome. Elife [Internet]. 2018 May 30 [cited 2019 Mar 1];7. Available from: https://elifesciences.org/articles/34408

9. Elsworth​ B, Lyon​ M, Alexander​ T, Liu​ Y, Matthews​ P, Hallett​ J, et al. The MRC IEU OpenGWAS data infrastructure. bioRxiv [Internet]. 2020 Aug 10 [cited 2020 Dec 23];2020.08.10.244293. Available from: https://www.biorxiv.org/content/10.1101/2020.08.10.244293v1

10. Han B, Duong D, Sul JH, de Bakker PIW, Eskin E, Raychaudhuri S. A general framework for meta-analyzing dependent studies with overlapping subjects in association mapping. Hum Mol Genet [Internet]. 2016 May 1 [cited 2022 Mar 1];25(9):1857–66. Available from: https://pubmed.ncbi.nlm.nih.gov/26908615/

11. Lin DY, Sullivan PF. Meta-Analysis of Genome-wide Association Studies with Overlapping Subjects. Am J Hum Genet [Internet]. 2009 Dec 11 [cited 2022 Mar 1];85(6):862. Available from: /pmc/articles/PMC2790578/

12. Giambartolomei C, Vukcevic D, Schadt EE, Franke L, Hingorani AD, Wallace C, et al. Bayesian test for colocalisation between pairs of genetic association studies using summary statistics. Williams SM, editor. PLoS Genet [Internet]. 2014 May 15 [cited 2014 Jul 12];10(5):e1004383. Available from: http://dx.plos.org/10.1371/journal.pgen.1004383

13. Consortium TGte. The GTEx Consortium atlas of genetic regulatory effects across human tissues. Science [Internet]. 2020 Sep 11 [cited 2020 Oct 8];369(6509):1318–30. Available from: http://science.sciencemag.org/

14. Võsa U, Claringbould A, Westra H-J, Bonder MJ, Deelen P, Zeng B, et al. Unraveling the polygenic architecture of complex traits using blood eQTL metaanalysis. bioRxiv [Internet]. 2018 Oct 19 [cited 2020 Oct 8];447367. Available from: https://europepmc.org/article/PPR/PPR59262

15. Haycock PC, Carolina Borges M, Burrows K, Lemaitre RN, Harrison S, Burgess S, et al. Design and quality control of large-scale two-sample Mendelian randomisation studies. medRxiv [Internet]. 2021 Aug 1 [cited 2021 Nov 11];2021.07.30.21260578. Available from: https://www.medrxiv.org/content/10.1101/2021.07.30.21260578v1

16. Howe LJ, Nivard MG, Morris TT, Hansen AF, Rasheed H, Cho Y, et al. Within-sibship GWAS improve estimates of direct genetic effects. bioRxiv [Internet]. 2021 Mar 7 [cited 2021 Nov 11];2021.03.05.433935. Available from: https://www.biorxiv.org/content/10.1101/2021.03.05.433935v1

17. Brumpton B, Sanderson E, Heilbron K, Hartwig FP, Harrison S, Vie GÅ, et al. Avoiding dynastic, assortative mating, and population stratification biases in Mendelian randomization through within-family analyses. Nat Commun 2020 111 [Internet]. 2020 Jul 14 [cited 2021 Nov 11];11(1):1–13. Available from: https://www.nature.com/articles/s41467-020-17117-4

18. Markozannes G, Kanellopoulou A, Dimopoulou O, Kosmidis D, Zhang X, Wang L, et al. Systematic review of Mendelian randomization studies on risk of cancer. BMC Med [Internet]. 2022 Dec 1 [cited 2022 Apr 2];20(1):1–22. Available from: https://bmcmedicine.biomedcentral.com/articles/10.1186/s12916-022-02246-y

19. Carter AR, Sanderson E, Hammerton G, Richmond RC, Davey Smith G, Heron J, et al. Mendelian randomisation for mediation analysis: current methods and challenges for implementation. Eur J Epidemiol [Internet]. 2021 May 1 [cited 2022 Feb 4];36(5):465–78. Available from: https://link.springer.com/article/10.1007/s10654-021-00757-1

20. Lewandowski RJ, Salem R, Mouli SK, Karp JK, Laws JL, Ryu RK, et al. Deaths Due to Cigarette Smoking for 12 Smoking-Related Cancers in the United States [Internet]. Vol. 175, JAMA Internal Medicine. 2015 [cited 2020 Oct 27]. p. 1574–6. Available from: https://www.ncbi.nlm.nih.gov/books/NBK294317/table/ch4.t1/

21. Table 4.1, Conclusions from Surgeon General’s report on active cigarette smoking and cancer [Internet]. Centers for Disease Control and Prevention (US); 2014 [cited 2020 Oct 27]. Available from: https://www.ncbi.nlm.nih.gov/books/NBK294317/table/ch4.t1/

22. Coussens LM, Werb Z. Inflammation and cancer [Internet]. Vol. 420, Nature. NIH Public Access; 2002 [cited 2020 Nov 2]. p. 860–7. Available from: /pmc/articles/PMC2803035/?report=abstract

23. National Cancer Institute. Surveillance, Epidemiology, and End Results Program [Internet]. [cited 2015 Aug 1]. Available from: https://seer.cancer.gov/

24. Tomasetti C, Vogelstein B. Variation in cancer risk among tissues can be explained by the number of stem cell divisions. Science [Internet]. 2015 Jan 2 [cited 2021 Nov 11];347(6217):78–81. Available from: https://pubmed.ncbi.nlm.nih.gov/25554788/

25. Viechtbauer W. Conducting meta-analyses in R with the metafor. J Stat Softw. 2010;36(3):1–48.

26. Viechtbauer W. Conducting Meta-Analyses in R with the metafor Package. J Stat Softw [Internet]. 2010 Aug 5 [cited 2022 Mar 1];36(3):1–48. Available from: https://www.jstatsoft.org/index.php/jss/article/view/v036i03

27. Watanabe M, Toh Y, Ishihara R, Kono K, Matsubara H, Murakami K, et al. Comprehensive registry of esophageal cancer in Japan, 2014. Esophagus [Internet]. 2022 Jan 1 [cited 2023 Apr 5];19(1). Available from: https://pubmed.ncbi.nlm.nih.gov/34550491/

28. Rahbar E, Ainsworth HC, Howard TD, Hawkins GA, Ruczinski I, Mathias R, et al. Uncovering the DNA methylation landscape in key regulatory regions within the FADS cluster. PLoS One [Internet]. 2017 Sep 1 [cited 2022 Aug 18];12(9). Available from: https://pubmed.ncbi.nlm.nih.gov/28957329/

29. Nishida N, Yano H, Nishida T, Kamura T, Kojiro M. Angiogenesis in Cancer. Vasc Health Risk Manag [Internet]. 2006 [cited 2022 Aug 18];2(3):213. Available from: /pmc/articles/PMC1993983/

30. Gharahkhani P, Fitzgerald RC, Vaughan TL, Palles C, Gockel I, Tomlinson I, et al. Genome-wide association studies in oesophageal adenocarcinoma and Barrett’s oesophagus: a large-scale meta-analysis. Lancet Oncol. 2016 Oct;17(10):1363–73.
