# Supplementary material for: The association between genetically elevated polyunsaturated fatty acids and risk of cancer
Source: eBioMedicine. 2023 Apr 20;91:104510. doi: 10.1016/j.ebiom.2023.104510 (PMC10148095; doi:10.1016/j.ebiom.2023.104510)
Supplement: Supplementary_materials_track_changes [file mmc3.docx]

[Supplementary methods 1](#_Toc100506957)

[Literature search 1](#_Toc100506958)

[Potential biases of the instrument selection strategy for secondary PUFA exposures 1](#_Toc100506959)

[Clumping procedure to identify independent genetic associations 2](#_Toc100506960)

[Defining genetic instruments for secondary exposures 3](#_Toc100506961)

[Making allowance for sample overlap between studies 3](#_Toc100506962)

[Sensitivity analyses for violations of Mendelian randomization analytical assumptions 4](#_Toc100506963)

[Colocalisation analysis 4](#_Toc100506964)

[Within-sibship MR analyses 5](#_Toc100506965)

[Effect decomposition analyses 5](#_Toc100506966)

[Modelling sources of heterogeneity in MR findings amongst cancer sites 6](#_Toc100506967)

[Supplementary results 7](#_Toc100506968)

[Sensitivity analyses for violations of Mendelian randomization analytical assumptions 7](#_Toc100506969)

[Colocalisation 7](#_Toc100506970)

[Effect decomposition analyses 8](#_Toc100506971)

[Study acknowledgements 9](#_Toc100506972)

[23andMe 9](#_Toc100506973)

[Esophageal Adenocarcinoma Study 10](#_Toc100506974)

[InterLymph 10](#_Toc100506975)

[Melanoma Meta-analysis Consortium 11](#_Toc100506976)

[References 13](#_Toc100506977)

# Supplementary methods

## Literature search

We searched for meta-analyses of observational studies of the association between polyunsaturated fatty acids (PUFAs) and cancer risk using the following search terms: ("meta-analysis" OR "meta analysis" OR "systematic review") AND (omega OR polyunsaturated) AND cancer. We also searched for randomized controlled trials (RCTs) of PUFAs and cancer risk using: ("randomized controlled trial" OR "randomised controlled trial") AND (omega or polyunsaturated) AND cancer. We searched PubMed up until 10 April 2022 with no language restrictions.

## Potential biases of the instrument selection strategy for secondary PUFA exposures

We investigated individual PUFA exposures in secondary analyses of the cancers that were associated with PUFA desaturase activity in the primary analyses. The investigated PUFA exposures were: alpha-linolenic acid (ALA;18:3n3), docosahexaenoic acid (DHA; 22:6n3), docosapentaenoic acid (DPA; 22:5n3), eicosapentaenoic acid (EPA, 20:5n3), total omega-3 fatty acids, arachidonic acid (AA; 20:4n6), dihomo-gamma-linolenic acid (DGLA; 20:3n6), gamma-linolenic acid (GLA; 18:3n6), linoleic acid (LA; 18:2n6) and total omega-6 fatty acids. Since summary data were available from multiple independent studies for each PUFA, we restricted our analyses to the single largest available study for each PUFA. This resulted in six of 10 instruments being defined using CHARGE and four of 10 being defined using UK Biobank. Due to methodological differences in how fatty acids were measured between the two studies (gas chromatography in CHARGE and nuclear magnetic resonance [NMR] in UK biobank) and the substantially different sample sizes (up to 114,999 in UK Biobank and up to 8866 in CHARGE), this instrument selection strategy reduces the comparability of Mendelian randomization (MR) results amongst different PUFA exposures. However, for our purposes we were not interested in comparing results between PUFA exposures, but mainly in determining whether associations with cancer exist for each PUFA.

Weak instrument bias (a type of measurement error) can also introduce bias, and its impact is likely to be more severe for instruments defined using CHARGE than for UK Biobank. (due to the relatively much smaller sample size of the former). In the presence of sample overlap, instruments estimated in small samples can introduce bias in MR estimates towards the confounded observational association, but in the absence of overlap this bias is towards the null. The degree of bias towards the confounded observational association depends on sample size, the degree of overlap of the instrument study (CHARGE or UK Biobank) with the cancer cases, and the size of the instrument effect on the exposure. Bias is expected to be minimal when study overlap is only with controls of a case-control study or when instrument strength is strong (conventionally defined as instrument F statistics much greater than 10)(1,2).

The individual cohorts of the CHARGE study were: ARIC, CHS, InCHIANTI, CARDIA and MESA. Given that these are population-based cohort studies in which the number of cancer cases as a proportion of the whole study is relatively low, we expect overlap with the cancer consortia, which are primarily based on case-control studies, to have been minimal, and we did not identify any instances of study overlap with the cancer consortia. Instrument strength in CHARGE also varied from 36 to 1929 (median=125, **Supplementary table 1**), indicating that even in the presence of considerable sample overlap, bias is unlikely to have been substantial(1). The UK Biobank study used to define instruments for PUFA exposures contained up to 114,999 participants. Of the 67 cancer outcomes investigated in primary MR analyses, 25 overlapped with UK Biobank. In these 25 studies, we estimate that 15% to 32% (median=24%) of the participants in the cancer study overlapped with the UK Biobank PUFA study (overlap of cases is likely to have been substantially lower) (**Supplementary table 2**). The estimated F statistics for the genetic instruments defined in UK Biobank varied from 81 to 374 (median=117, **Supplementary table 1**), indicating that bias from sample overlap with UK Biobank is likely to have been minimal(1). A caveat is that these F statistics may be over-estimated, indicating that we can’t entirely rule out some materially important weak instrument bias. However, given that overlap of CHARGE and UK Biobank with most of the cancer studies is likely to be low, this bias is more likely to be towards the null for most results.

## Clumping procedure to identify independent genetic associations

We identified independent SNPs associated with PUFA exposures (either the fatty acid biomarkers of D5D and D6D activity or individual omega 3 and omega 6 PUFAs), using a conventional threshold of GWAS statistical significance (P<5x10^-8^) and with linkage disequilibrium (LD) clumping to prune for independence. We used an r^2^ threshold of 0.001 and a base pair window of 10,000 kb. For European ancestry studies, we used 10,000 randomly selected individuals from UK Biobank as the reference panel (all analyses involving individual level data from UK Biobank were accessed under project number 15825). For East Asian ancestry studies, we used 504 East Asian ancestry individuals from the 1000 genomes project as the reference panel(3).

## Instrument for PUFA desaturase activity

The C allele of rs174546 is associated with higher PUFA desaturase activity in European and East Asian ancestry studies (supplementary table 1). It is also associated with higher AA and higher EPA levels. In European and East Asian ancestry studies from Japan and Northern China, the C allele is also the major allele (T allele is the minor allele). However in South East Asian populations the C allele tends to be the minor allele and the T allele the major allele. FADS1/D5D converts the omega 6 PUFAs DGLA into AA and omega 3 PUFAs eicosatetraenoic acid (ETA) to EPA, while FADS2/D6D converts the omega 6 PUFAs LA into gamma linolenic acid (GLA) and omega 3 PUFAs ALA into stearidonic acid (SA).

## Defining genetic instruments for secondary exposures

Secondary Exposures: We defined a set of secondary exposures as omega 3 or omega 6 PUFAs that could be instrumented by genetic variants outside the FADS region, defined as genomic coordinates [GRCh37] chr11: 61,060,452-62,159,523. To do this, we obtained GWAS summary data for individual PUFAs from the following six studies: TwinsUK/KORA (Cooperative Health Research in the Region Augsburg)(4), the SCHS(5), Kettunen et al(6), the Framingham study(7), CHARGE^16^ and UK Biobank (downloaded from OpenGWAS <https://gwas.mrcieu.ac.uk/>)(8,9). Eighteen PUFAs were measured across the six studies (**Supplementary table 2**). Four of the 18 PUFAs could not be clearly classified as omega 3 or omega 6 PUFAs and were therefore excluded **(Supplementary table 2**). We next identified SNPs associated with the individual PUFAs using LD clumping (details described above). When multiple studies were available for the same PUFA, we restricted our analyses to the single largest available study for that PUFA (potential bias from this instrument selection strategy, which we consider to be minimal, is discussed above). For studies of European ancestry, this procedure identified 124 SNPs associated with 14 PUFAs. Four of the 14 PUFAs could not be instrumented by genetic variation outside the *FADS* region and were therefore excluded from secondary MR analyses. The retained secondary PUFA exposures included five omega 3 fatty acids (variation explained, with or without the FADS region in brackets): ALA (3.76% vs 0.36%), DHA (5.07% vs 1.58%), DPA (9.93% vs 2.52%) and total omega-3 fatty acids (8.94% vs 2.19%). Secondary PUFA exposures also included the following five omega 6 PUFAs: GLA (5.31% vs 1.94%), LA (4.01% vs 2.39%), AA (30.9% vs 0.47%), DGLA (11.86% vs 3.75%) and total omega-6 fatty acids (4.62% vs 4.59%). All instruments excluding the FADS region had an F statistic ≥36 (median=81; max=150, supplementary table 2), indicating that bias from weak instruments is unlikely to be substantial(1). Seven of 10 secondary PUFA exposures could be instrumented by ≥2 independent SNPs outside of the FADS region, while three could be instrumented by a single SNP outside the FADS region. For studies of East Asian ancestry, only one PUFA was associated with variation outside the *FADS* region. We therefore excluded studies of East Asian ancestry from secondary MR analyses. Further details on these PUFAs and their genetic instruments can be found in **Supplementary tables 2-3.**

## Combining results across studies

When summary data were available for the same cancer from multiple independent studies, we conducted MR analyses separately for each study, and then combined the MR results by fixed effects meta-analysis using inverse variance weights (“MR-by-study” approach). An alternative approach would be to first combine the summary data across studies, by fixed effects meta-analysis, and then to conduct MR analyses on the pooled dataset (“MR-of-pooled-study” approach). In our case, the first approach (MR-by-study) was preferable because for some cancers we combined results across European and Easy Asian ancestry studies and the instrument for PUFA desaturase activity has a different effect size in European and East Asian populations. The alternative approach is less appropriate when combining data across different populations with distinct instruments. Both approaches should however give the same result when only a single variant is used to instrument an exposure, as was the case in our primary analyses, but might be expected to give different results when the instrument is comprised of multiple variants, as was the case in secondary analyses. To see if that was the case, we compared the two approaches in an MR analysis of secondary PUFAs and lung cancer, where summary genetic data were derived from ILCCO and UK Biobank. The two approaches gave almost identical results (supplementary figure 21).

## Making allowance for sample overlap between studies

We made allowance for sample overlap in analyses that combined results from, or tested for differences between, studies. In meta-analyses of cancer results from the same biological system or in meta regression analyses, we pruned MR results to have no overlapping cases (we acknowledge that a small degree of overlap, even after this procedure, cannot be entirely excluded). This involved the following steps. First, we pruned MR results so that only a single cancer was included for each of the following sites: breast, bowel, stomach, kidney, liver, mouth & throat, ovary and prostate, retaining the cancer with the most cases (e.g. overall breast cancer was included but breast cancer subtypes were excluded). Due to considerable overlap in cases amongst different cancer definitions in UK Biobank, we excluded the following cancer datasets generated in UK Biobank: overall cancer (case overlap with all site specific cancers), cancer of digestive organs (overlap with site-specific digestive system cancers), respiratory and intrathoracic cancer (overlap with lung cancer), lymphoid leukaemia (overlap with leukaemia), brain cancer (overlap with central nervous system and eye cancer), female genital cancer (overlap with endometrial cancer), non-melanoma skin cancer (overlap with squamous cell carcinoma and basal cell carcinoma), overall skin cancer (overlap with melanoma, basal cell carcinoma and squamous cell carcinoma), urinary tract cancer (overlap with bladder cancer). Further details on the above cancers can be found in **Supplementary table 5**.

There was considerable overlap in controls amongst cancer datasets derived from the following biobanks or consortia: UK Biobank, FinnGen, Biobank Japan and InterLymph. To make allowance for control overlap, we increased the standard errors for all MR results generated with participants from one of the latter studies, using a decoupling transformation(10). This involved the following steps. First, we estimated a correlation matrix for the MR results using the method of Lin and Sullivan(11) (this step involves using the number of overlapping samples to infer the correlation matrix). We then used the correlation matrix to decouple the log odds ratios and standard errors for MR results with overlapping controls. The decoupled log odds ratios and standard errors were then used in the following downstream analyses: meta-regression analyses to identify sources of heterogeneity in MR results amongst cancers; random effects meta-analyses of MR results from the same biological system (reproductive cancers, urinary cancers, blood cancers and nervous system cancers); and in Q and Z tests for differences in MR findings amongst colorectal and lung cancer tumour subtypes.

## Sensitivity analyses for violations of Mendelian randomization analytical assumptions

We conducted three sets of analyses to assess the sensitivity of our main findings to violations of analytical assumptions (name of assumption in backets): colocalisation analysis (no genomic confounding), within-sibship MR analyses (no confounding by population stratification) and effect decomposition analyses (no horizontal pleiotropy with smoking).

### Colocalisation analysis

To assess the sensitivity of our findings to genomic confounding, we used colocalisation analysis to estimate posterior probabilities for the sharing of the same causal variant across selected cancers, PUFA desaturase biomarkers and *FADS1/2* gene expression(12). For these analyses we used genetic association results across 500,000 base pairs at the *FADS* gene cluster on chromosome 11q12.2-13.1 and focused on the cancers identified in the primary MR analysis. Genetic association results for *FADS1* and *FADS2* gene expression in Europeans were obtained from the Genotype-Tissue Expression (GTEx) project (version 8) for liver, adipose and blood tissues(13) and from eQTLGen in blood(14). Genetic association results for *FADS1* and *FADS2* gene expression in East Asians were obtained from Biobank Japan in blood and white blood cell subfractions (B cells, CD8 T cells, monocytes and natural killer [NK] cells; other tissues were unavailable)(14). We also selected tissues that were most representative of cancer sites associated with the PUFA desaturase biomarker in the primary MR analysis. Colocalisation analyses were conducted using the coloc package(12) and were conducted in a pairwise fashion, testing each cancer against each of three exposure traits: the PUFA desaturase biomarker, *FADS1* gene expression or *FADS2* gene expression. We set the prior probability that a randomly selected SNP was associated with the cancer or exposure trait to 1x10^-4^ or with both traits to 1x10^-6^ (these are the default priors of the coloc package)(12). Regional association plots, generated using the gassocplot package, were used to visualise genetic association results(15). We defined strong evidence for colocalisation as a poster probability (PP) for sharing the same causal variant (hypothesis 4 [H4])≥80%). Weak evidence was defined as PP_H4_≤25% and moderate evidence defined as 25%<PP_H4_<80%.

### Within-sibship MR analyses

To assess the sensitivity of our results to confounding by population stratification, indirect genetic effects or assortative mating, we conducted within-sibship MR analyses using data on 19,588 sibships from UK Biobank(16,17). Analyses were conducted on overall cancer (to boost power), overall skin cancers, colorectal cancer and lung cancer. To boost power, we also combined colorectal, lung and skin cancers.

### Effect decomposition analyses

In effect decomposition analyses, we estimated the association of rs174546 with 36 biomedically important characteristics, including lipids, smoking and anthropometrics, and then modelled the extent to which any identified associations (defined as P values <0.0013 [alpha of 0.05/36]) could explain our findings. The 36 selected characteristics were defined as traits categorised as risk factors in Open GWAS (<https://gwas.mrcieu.ac.uk/>)(18). For non-smoking characteristics associated with rs174546, we then searched for evidence that the identified characteristics are causally associated with colorectal cancer, lung cancer and basal cell carcinoma using a systematic review of MR studies of cancer(19). We then estimated the extent to which the identified characteristics could explain the association between rs174546 and the latter cancers using the product of coefficients method(20). The result of this analysis can be interpreted as the association between rs174546 and cancer mediated by the selected characteristic (also known as the indirect effect).

For smoking characteristics, we conducted additional association analyses of rs174546 and rs2524299 (the variant most strongly associated with lung cancer in the *FADS* region) with cigarettes smoked per day (N=249,752) and smoking status (ever versus never, N=607,291) in GSCAN (GWAS & Sequencing Consortium of Alcohol and Nicotine use), and with a lifetime smoking score in UK Biobank (N=462,690). The lifetime smoking score captures information on smoking status (ever versus never) as well as smoking duration, heaviness and cessation in ever smokers. Genetic instruments based on the lifetime smoking score can therefore be applied to samples that have not been stratified on smoking status, which was the case for all cancers included in our study with the exception of lung cancer. To assess whether smoking could account for our primary MR findings, we then conducted additional MR analyses of lifetime time smoking on colorectal cancer, lung cancer and basal cell carcinoma, as well as cigarettes smoked per day on lung cancer in ever smokers, using a random effects IVW model(8) (see **Supplementary tables 15 & 16** for details of genetic instruments). We then estimated the effect of rs174546 and rs2524299 on cancer mediated by the latter smoking characteristics using the products of coefficient method(20).

In a scenario in which the biomedical characteristic can account for our MR findings, we expect the mediated effect to be similar to the observed effect of rs174546 or rs2524299 on cancer (also known as the total effect)(20). A limitation is that, even in a scenario in which the effect is entirely mediated by the characteristic, due to measurement error we do not expect them to be identical. We tested for a difference between the mediated (i.e. indirect) and total effects using a Z test. We interpreted similarity between the mediated and total effects as compatible with either vertical or horizontal pleiotropy in our MR findings, whereas dissimilarity was interpreted as evidence against both types of pleiotropy. When we could not identify published MR studies of the characteristic and cancer, we estimated our own causal effect using the Wald ratio method (the effect of rs174546 on cancer divided by its effect on the characteristic). This can be interpreted as the expected effect of the characteristic on cancer, assuming the relationship between rs174546 and cancer is entirely mediated by that characteristic, rs174546 is truly associated with the characteristic, and that rs174546 is not associated with confounders of the relationship between the characteristic and cancer.

## Modelling sources of heterogeneity in MR findings amongst cancer sites

To identify potential sources of heterogeneity in MR findings amongst cancers, we assessed the impact of cancer-level characteristics on our results using a meta-regression approach. We modelled the following cancer-level characteristics: smoking (i.e. whether smoking is one of the accepted causes of the cancer(21,22)), chronic inflammation (whether the cancer has an accepted relationship to a chronic inflammatory condition(23)), cancer incidence(24), survival time(24), median age at diagnosis(24) and tissue-specific rates of stem cell division(25).

We regressed the MR results for each cancer on the cancer-level characteristic and tested for a non-zero slope using a random effects model (fitted using a maximum-likelihood estimator and inverse variance weights implemented in the metafor package(26)). In this analysis, the MR result for each cancer can be considered the outcome, the cancer-level characteristic is the independent variable and a non-zero slope can be interpreted as evidence that the magnitude of the MR result varies by the cancer-level characteristic. To make allowance for sample overlap between cancer studies, we first pruned the MR results to have minimal overlap in cases and, in sensitivity analyses, inflated the standard errors using a decoupling transformation(10) (see above for more details). Sensitivity analyses further assessed the impact of reclassifying cancers into alternative groupings. These included: adding the result for laryngeal squamous carcinoma (identified by a search of the GWAS catalog) to the group of smoking-related cancers (this cancer was otherwise excluded from all analyses); recoding endometrial cancer (which shows a protective association with smoking in observational studies) as a smoking-related cancer; and including cancers associated with infectious agents in the chronic inflammatory group of cancers (see below for further details of how we defined this group).

We defined “smoking-related” cancers using the 2014 US Surgeon General’s Report(21,22). According to the report, the evidence is sufficient to infer that smoking causally increases the risk of the following 12 cancers: bladder cancer, cervical cancer, colorectal cancer, esophageal cancer, kidney cancer, laryngeal cancer, acute myeloid leukemia, liver cancer, lung cancer, oral cavity and pharyngeal cancer, pancreatic cancer and stomach cancer. We defined these cancers as “smoking-related” i.e. smoking is one of the accepted causes of the cancer. According to the report, there is either no, or insufficient, evidence to infer that smoking causally increases risk of the following cancers: brain cancer, breast cancer, prostate cancer, ovarian cancer and endometrial cancer. The report also concluded that smoking reduces risk of endometrial cancer in postmenopausal women. All cancers that did not overlap with the 12 “smoking-related cancers” above were defined as cancers with an uncertain relationship to smoking, referred to as “non-smoking cancers” for short. We had summary data for all smoking-related cancers, except for laryngeal cancer. However, our genetic instrument (rs174546) is in strong LD (r^2^=0.93) with a published GWAS hit for laryngeal cancer in East Asians(24). We therefore conducted additional meta-regression analyses with this cancer included as a sensitivity analysis.

We also compared cancers grouped according to their relationship to chronic inflammatory conditions as defined by Coussens et al(23). Although this may be an outdated reference, we consider it to be reasonably accurate for these analyses, which we consider to be exploratory and hypothesis-generating. According to Coussens et al(23), chronic inflammatory conditions increase risk of the following cancers (condition in brackets): mesothelioma (asbestosis & silicosis), lung cancer (bronchitis), bladder cancer (cystitis & bladder inflammation), oral squamous cell carcinoma (gingivitis and lichen planus), colorectal cancer (inflammatory bowel disease: Crohn’s disease & ulcerative colitis), vulvar squamous cell carcinoma (lichen sclerosus), pancreatic cancer (pancreatitis), esophageal cancer (reflex esophagitis & Barett’s esophagus), salivary gland carcinoma (sialadentis), mucosa-associated lymphoid tissue (MALT) lymphoma (Sjogren syndrome & hashimoto’s thyroiditis) and melanoma (skin inflammation). We defined these cancers as “chronic inflammatory cancers”. All cancers that did not overlap with this group were defined as “other inflammatory cancers”. Summary data were available for all “chronic inflammatory” cancers, except for vulvar squamous cell carcinoma and salivary gland carcinoma. As a sensitivity analysis, we expanded the “chronic inflammatory” group to include cancers with a relationship to infectious agents (agent in brackets): cholangiosarcoma and colon carcinoma (liver flukes); gall bladder cancer (bacteria); gastric adenocarcinoma, gastric MALT lymphoma (Helicobacter pylori); Hepatocellular carcinoma (Hepatitis B and/or C virus); B-cell non-Hodgkin lymphoma, Burkitt lymphoma (Epstein-Barr Virus); Non-Hodgkin lymphoma, squamous cell carcinomas, Kaposi’s sarcoma (human immunodeficiency virus, human herpesvirus type 8); skin carcinoma in draining sinuses (bacterial infection); ovarian carcinoma, cervical/anal carcinoma (Gonnorrhoea, chlamydia, human papillomavirus); bladder, liver and rectal carcinoma and follicular lymphoma of the spleen (Schistosomiasis). Summary data were available for all “infectious agent related” cancers except gall bladder cancer, Burkitt lymphoma and anal carcinoma.

A caveat is that the meta-regression procedure is likely to provide overly precise confidence intervals(27), and findings are also likely to be susceptible to confounding by other cancer-level characteristics. We therefore interpret results as exploratory and hypothesis-generating. We used an alpha threshold of 0.05 to prioritise potential sources of heterogeneity for replication in future studies.

# Supplementary results

## Sensitivity analyses for violations of Mendelian randomization analytical assumptions

### Colocalisation

As a sensitivity analysis for genomic confounding, we assessed evidence for colocalisation of selected cancers with PUFA desaturase activity and with expression of the *FADS1* and *FADS2* genes in various tissues (**Supplementary figure 10 and Supplementary table 10**). Overall, the evidence for colocalisation was strongest for colorectal cancer: the posterior probabilities for a shared causal variant (PP_H4_) with PUFA desaturase activity and with *FADS1* gene expression in the sigmoid colon were ≥96% in European ancestry studies (with similarly strong evidence in East Asian ancestry studies). Findings for esophageal squamous cell carcinoma also showed strong evidence for colocalisation with PUFA desaturase activity (PP_H4_=87%), as well as with *FADS1* and *FADS2* gene expression in blood subtypes (PP_H4_≥76% in CD4 T, CD8 T and NK cells), in studies of East Asian ancestry. Further supporting colocalisation, the lead variants for esophageal squamous cell carcinoma and for *FADS1* gene expression in esophageal tissue are strongly correlated with each other in studies of European and East Asian ancestry (r^2^>0.8).

Colocalisation evidence for other cancers was less robust. Lung cancer did not consistently colocalise with different biomarkers for PUFA desaturase activity (PP_H4_=42% for the D5D biomarker and PP_H4_=68% for the D6D biomarker) and evidence for colocalisation with *FADS1* gene expression in lung tissue was moderate (PP_H4_=69%). On the other hand, there was strong evidence for colocalisation of lung cancer with *FADS1* gene expression in adipose subcutaneous tissue (PP_H4_=96%). Basal cell carcinoma did not colocalise with PUFA desaturase activity, *FADS1* gene expression or *FADS2* gene expression (PP_H4_<8%).

### Effect decomposition analyses

We found that the instrument for PUFA desaturase activity (rs174546) was weakly associated (SD change [95% confidence interval] per copy of the C allele) with LDL cholesterol (0.01 [0.01 to 0.02]), total cholesterol (0.05 [0.04 to 0.05]), triglycerides (-0.05 [-0.05 to -0.04]), HDL cholesterol (0.04 [0.03 to 0.05]), height (0.01 [0.01 to 0.02]), platelet count (-0.03 [-0.04 to -0.02]), heart rate (-0.03 [-0.04 to -0.02]) and age at menopause (0.02 [0.01 to 0.03]) **(Supplementary table 11 and Supplementary figure 12**). By comparison, the SD change in AA:DGLA (biomarker for D5D activity) per copy of the C allele was 0.87 (0.84 to 0.89) in European ancestry individuals (**Supplementary table 1**).

In a systematic review of MR studies(19), the OR (95% CI) for colorectal cancer per SD increase in genetically proxied height was 1.04 (1.00 to 1.08), for LDL cholesterol was 1.14 (1.04 to 1.25), for triglycerides was 0.93 (0.84 to 1.03), for total cholesterol was 1.09 (1.01 to 1.18), for HDL cholesterol was 1.03 (0.93 to 1.15) and for age at menopause was 1.00 (0.82 to 1.22). For lung cancer, the OR (95% CI) per SD increase in genetically proxied height was 1.07 (1.00 to 1.15), for platelet count was 1.00 (1.00 to 1.00), for LDL cholesterol was 0.90 (0.84 to 0.97), for triglycerides was 0.98 (0.91 to 1.06), for total cholesterol was 0.94 (0.88 to 1.01) and for HDL cholesterol was 1.01 (0.95 to 1.08)(19). We then estimated the effect of rs174546 on colorectal cancer and lung cancer mediated by the latter characteristics (also known as the indirect effect, **Supplementary table 11**). These were consistently less than 1.01 and smaller than the total effect of rs174546 on colorectal cancer (OR=1.06 95% CI: 1.05 to 1.08) and lung cancer (OR=1.03 95% CI: 1.01 to 1.06) (P values for difference between indirect and total effects ≤ 0.013). These results indicate that the identified characteristics cannot account for the observed associations of rs174546 with colorectal cancer and lung cancer and, consequently, that pleiotropy with these characteristics cannot explain our MR findings. A caveat is that we have likely underestimated the CIs for the indirect effects, since these analyses did not take into account uncertainty in the effect of rs174546 on the above characteristics.

We did not identify published MR studies for associations of platelet count and heart rate with colorectal cancer or associations of heart rate and age at menopause with lung cancer. We were therefore unable to estimate indirect effects for these characteristics. However, to account for the observed association of rs174546 with colorectal cancer, lower platelet count and lower heart rate would have to be associated with unusually strong ORs for colorectal cancer of 8.50 (4.59 to 15.72) per SD decrease. We therefore consider it unlikely that these characteristics can account for our colorectal cancer MR results. ORs for lung cancer per SD decrease in heart rate would have to be 3.06 (1.31 to 7.17) and per SD increase in age at menopause would have to be 5.52 (95% CI 1.51 to 20.24), which includes plausible effect sizes in the confidence intervals. We therefore cannot exclude the possibility that these characteristics can partly explain the lung cancer findings.

We conducted additional effect decomposition analyses to assess whether our findings could be explained by smoking. Per copy of the allele associated with higher PUFA desaturase activity (allele C) for rs174546, the OR for ever smoking status was 1.00 (95% CI: 0.99 to 1.01), the SD change in cigarettes smoked per day was 0.01 (95% CI 0.00 to 0.03) and the SD change in a lifetime smoking score was -0.001 (-0.004 to 0.002). In effect decomposition analyses, we found that genetically proxied lifetime smoking could not account for the observed association between rs174546 and selected cancers (P values ≤ 4.72x10^-03^ for difference between total and indirect smoking effects) (**Supplementary table 12**). In further analyses of rs2524299 (the strongest variant for lung cancer in the *FADS* region), the OR for lung cancer in ever smokers due to genetically proxied cigarettes smoked per day (1.01 [95% CI: 1.01 to 1.02]) was smaller than the observed association (OR=1.10 [95% CI: 1.05 to 1.14]) (P value = 3.53x10^-04^ for difference between total and indirect effects), suggesting that pleiotropy with smoking heaviness does not explain our MR results. Similar findings were observed for rs174546 (**Supplementary table 13**). A caveat is that these analyses did not model uncertainty in the effect of these genetic variants on smoking, meaning we are likely to have underestimated the CIs for the indirect effects.

**Modelling sources of heterogeneity amongst cancers**

There was little evidence that MR results varied by cancer incidence, survival time, median age-at-diagnosis, or tissue-specific rates of stem cell division (P≥0.56, **Supplementary figures 13-16; Supplementary table 14**). MR results tended to be stronger for 13 “smoking-related” cancers (OR= 1.05 [95% CI: 1.02 to 1.09]) compared to 30 “non-smoking” cancers (OR=1.01 [95% CI: 0.99 to 1.03]) (P = 0.003, for difference between ORs) (**Supplementary figure 17**). MR results were stronger for nine cancers with an accepted relationship to chronic inflammatory conditions (OR=1.05 [95% CI: 1.02 to 1.09]) compared to 34 other cancers (OR=1.01 [95% CI: 0.99 to 1.03]) (P=0.004, for the difference in OR) (**Supplementary figure 18**). MR results also tended to be stronger for digestive (OR=1.05 [95% CI: 1.01 to 1.10]) compared to non-digestive system cancers (OR=1.01 [95% CI: 0.99 to 1.03]) (P=0.019 for difference in OR) (**Supplementary figure 19**). Results were similar in various sensitivity analyses, including analyses that adjusted for potential sample overlap between studies or that reclassified cancers into different groupings (**Supplementary table 14**).

# Supplementary discussion

## Proxying multiple independent points in the PUFA biosynthesis pathway

Our analysis would have been significantly strengthened by the use of multiple independent genetic proxies for different points in the PUFA biosynthesis pathway, e.g. for the metabolic steps catalysed by the enzymatic products of *FADS1*, *FADS2* and *ELOVL2* (**Supplementary figure 2**). This proved impossible for *FADS1* and *FADS2,* due to their very close physical proximity. We did however employ some SNPs as instruments that probably work through modulation of *ELOVL2* gene expression levels. For example, rs3734398, rs3798713 and rs2180725, located in the *ELOVL2* gene, were amongst the genetic instruments used for docosapentaenoic acid (DPA), eicosapentaenoic acid (EPA) and linoleic acid (LA) (**Supplementary table 3**). rs3734398 resides in the 3 prime untranslated region, while rs3798713 and rs2180725 are intronic to *ELOVL2*. Since EPA is the substrate for the ELOVL2 enzyme, associations between rs3798713 and this fatty acid may reflect ELOVL2 activity levels. Per SD increase in EPA proxied by rs3798713, the odds ratio for colorectal cancer was 0.99 (95% confidence interval 0.84 to 1.17), for lung cancer was 1.09 (0.89 to 1.33) (Figure 3) and for basal cell carcinoma was 0.99 (0.8 to 1.22) (Supplementary figure 9) (MR results for EPA excluding the FADS region reflect rs3798713). Although these results could be interpreted as indicating no association between ELOVL2 enzyme activity and the selected cancers, interpretation is complicated by the relatively low variance explained in EPA levels by rs3798713 (0.55%). Thus, it is unclear whether this result reflects lack of power or is a true null. For example, the confidence intervals include an effect size that is similar in magnitude to the associations observed for arachidonic acid and colorectal cancer (Figure 2).

## Role of FADS activity

Our primary instrument, rs174546, which we used as a proxy for PUFA desaturase activity, resides in the 3 prime untranslated region of the *FADS1* gene and is also intronic to the *FADS2* gene. There is evidence that the variant regulates expression of both *FADS1* and *FADS2* through modulation of an enhancer element located between the two genes. For example, rs174537 (r2=0.989 with rs174546) is correlated with DNA methylation in a putative enhancer located between the *FADS1* and *FADS2* promoter regions(28). Our instrument, or the causal variant(s) in LD with our instrument, is likely to directly affect levels of D5D and D6D enzymes via *FADS1* and *FADS2* gene expression. Since we were unable to distinguish between activity of the two, we interpret our instrument as a proxy for both D5D and D6D desaturase activity.

D5D and D6D metabolise omega 3 and omega 6 PUFAs, which are hypothesised to have opposing effects on cancer risk - the omega 3 PUFAs having anti-inflammatory anti-carcinogenic effects and omega 6 PUFAs have pro-inflammatory pro-carcinogenic effects. These opposing effects would be expected to attenuate cancer findings based on *FADS* genetic variants towards the null. As to why we observed positive associations with cancer risk (and not null associations), this might reflect a relatively higher consumption of omega 6 PUFAs compared to omega 3 PUFAs in the European and East Asian ancestry studies included in our analyses. In other words, omega 6 PUFAs may outcompete omega 3 PUFAs for D5D and D6D, due to their greater abundance. Alternatively, perhaps the observational associations for omega3 and cancer risk are not causal and only omega 6 PUFAs have a true causal effect on cancer. It would be interesting to replicate our study in a population where omega 3 PUFA consumption outweighs the consumption of omega 6 PUFAs, to see whether associations with cancer risk reversed direction or attenuated to the null.

## Clinical implications of global variation in rs174546 C allele prevalence

To improve the safety profile of potential interventions on PUFAs, interventions could be targeted to carriers of the C allele of rs174546 (the allele associated with higher PUFA desaturase activity, higher risk of selected cancers but lower risk of inflammatory bowel disease), since such individuals would be expected to obtain more benefit than carriers of the T allele. Such an intervention would have to take into account C and T allele prevalence. For example, the intervention would probably not be worth the increased cost of screening in populations where the T allele is very rare.

Potential carcinogenic pathways

The pro-inflammatory eicosanoid pathway is not the only biological mechanism that could account for our findings. For example, AA is metabolised into leukotrienes and pro-inflammatory hydroxyeicosatetraenoic acids (HETEs), via lipoxygenase (LOX), which are hypothesised to have a role in carcinogenesis(28). Cytochrome P450 (CYP) ω-hydroxylase is also able to metabolise AA into HETEs(28). CYP epoxygenase generates AA epoxides or epoxyeicosatrienoic acids (EETs), which could play a role in carcinogenesis via their influence on cellular proliferation, survival and angiogenesis(28). Thromboxane, another product of COX mediated metabolism of AA, is a clotting factor that leads to platelet aggregation, which could influence carcinogenesis through its regulation of angiogenesis(29).

# Study acknowledgements

## 23andMe

Participants provided informed consent and participated in the research online, under a protocol approved by the external AAHRPP-accredited IRB, Ethical & Independent Review Services (E&I Review). The full GWAS summary statistics for the 23andMe discovery data

set will be made available through 23andMe to qualified researchers under an agreement with 23andMe that protects the privacy of the 23andMe participants. Please visit https://research.23andme.com/collaborate/#dataset-access/

for more information and to apply to access the data.

Esophageal Adenocarcinoma Study

Esophageal adenocarcinoma GWAS(30): The MD Anderson controls were drawn from dbGaP (study accession: phs000187.v1.p1). Genotyping of these controls was performed through the University of Texas MD Anderson Cancer Center (UTMDACC) and the Johns Hopkins University Center for Inherited Disease Research (CIDR). We acknowledge the principal investigators of this study: Christopher Amos, Qingyi Wei and Jeffrey E. Lee. Controls from the Genome-Wide Association Study of Parkinson Disease were obtained from dbGaP (study accession: phs000196.v2.p1). This work, in part, used data from the National Institute of Neurological Disorders and Stroke (NINDS) dbGaP database from the CIDR:NeuroGenetics Research Consortium Parkinson’s disease study. We acknowledge the principal investigators and coinvestigators of this study: Haydeh Payami, John Nutt, Cyrus Zabetian, Stewart Factor, Eric Molho and Donald Higgins. Controls from the Chronic Renal Insufficiency Cohort (CRIC) were drawn from dbGaP (study accession: phs000524.v1.p1). The CRIC study was performed by the CRIC investigators and supported by the National Institute of Diabetes and Digestive and Kidney Diseases (NIDDK). Data and samples from CRIC reported here were supplied by NIDDK Central Repositories. This report was not prepared in collaboration with investigators of the CRIC study and does not necessarily reflect the opinions or views of the CRIC study, the NIDDK Central Repositories or the NIDDK. We acknowledge the principal investigators and the project officer of this study: Harold I. Feldman, Raymond R. Townsend, Lawrence J. Appel, Mahboob Rahman, Akinlolu Ojo, James P. Lash, Jiang He, Alan S. Go and John W. Kusek.

## InterLymph

The Health Professionals Follow-up Study was supported in part by National Institutes of Health grants UO1 CA167552, R01 CA149445, and R01 CA098122. The authors would like to acknowledge the contribution to this study from central cancer registries supported through the Centers for Disease Control and Prevention’s National Program of Cancer Registries (NPCR) and/or the National Cancer Institute’s Surveillance, Epidemiology, and End Results (SEER) Program. Central registries may also be supported by state agencies, universities, and cancer centers. Participating central cancer registries include the following: Alabama, Alaska, Arizona, Arkansas, California, Colorado, Connecticut, Delaware, Florida, Georgia, Hawaii, Idaho, Indiana, Iowa, Kentucky, Louisiana, Massachusetts, Maine, Maryland, Michigan, Mississippi, Montana, Nebraska, Nevada, New Hampshire, New Jersey, New Mexico, New York, North Carolina, North Dakota, Ohio, Oklahoma, Oregon, Pennsylvania, Puerto Rico, Rhode Island, Seattle SEER Registry, South Carolina, Tennessee, Texas, Utah, Virginia, West Virginia, Wyoming. The authors assume full responsibility for analyses and interpretation of these data. We would also like to thank the participants and staff of the Health Professionals Follow-up Study for their valuable contributions. The study protocol was approved by the institutional review boards of the Brigham and Women’s Hospital and Harvard T.H. Chan School of Public Health, and those of participating registries as required.

The Nurses’ Health Study was supported in part by National Institutes of Health grants UM1 CA186107, P01 CA87969, R01 CA49449, R01 CA149445, R01 CA098122 and R01 CA134958. The authors would like to acknowledge the contribution to this study from central cancer registries supported through the Centers for Disease Control and Prevention’s National Program of Cancer Registries (NPCR) and/or the National Cancer Institute’s Surveillance, Epidemiology, and End Results (SEER) Program. Central registries may also be supported by state agencies, universities, and cancer centers. Participating central cancer registries include the following: Alabama, Alaska, Arizona, Arkansas, California, Colorado, Connecticut, Delaware, Florida, Georgia, Hawaii, Idaho, Indiana, Iowa, Kentucky, Louisiana, Massachusetts, Maine, Maryland, Michigan, Mississippi, Montana, Nebraska, Nevada, New Hampshire, New Jersey, New Mexico, New York, North Carolina, North Dakota, Ohio, Oklahoma, Oregon, Pennsylvania, Puerto Rico, Rhode Island, Seattle SEER Registry, South Carolina, Tennessee, Texas, Utah, Virginia, West Virginia, Wyoming. The authors assume full responsibility for analyses and interpretation of these data. We also thank the participants and staff of the Nurses' Health Study for their valuable contributions. The study protocol was approved by the institutional review boards of the Brigham and Women’s Hospital and Harvard T.H. Chan School of Public Health, and those of participating registries as required.

The Utah hematological malignancy study was supported by funding from the National Cancer Institute (NCI) grant R01 CA134674 (to NJC). Data collection in Utah was supported by the Utah Population Database (UPDB) and Utah Cancer Registry (UCR). The UPDB is supported by Huntsman Cancer Institute (HCI, including Huntsman Cancer Foundation, HCF), the University of Utah, and NCI grant P30 CA2014. The UCR is funded by the NCI's SEER Program, Contract No. HHSN261201800016I, the US Center for Disease Control and Prevention's National Program of Cancer Registries (Cooperative Agreement No. NU58DP006320), the University of Utah, and HCF. The study thanks all study participants, ascertainment, laboratory, and research informatics teams at HCI, and the Hematology Biobank, Justin Williams, Brandt Jones, Myke Madsen, Brian Avery and Rob Sargent for their important contributions.

## Melanoma Meta-analysis Consortium

**GenoMEL**

The GenoMEL study (<http://www.genomel.org/>) was funded by the European Commission under the 6^th^ Framework Programme (contract no. LSHC-CT-2006-018702), by Cancer Research UK Programme Awards (C588/A4994 and C588/A10589), by a Cancer Research UK Project Grant (C8216/A6129) and by a grant from the US National Institutes of Health (NIH; CA83115). This research was also supported by the intramural Research Program of the NIH, National Cancer Institute (NCI), Division of Cancer Epidemiology and Genetics.

This study makes use of data generated by the Wellcome Trust Case Control Consortium (<http://www.wtccc.org.uk/>). A full list of the investigators who contributed to the generation of the data is available from their website (see URLs). Funding for the project was provided by the Wellcome Trust under award 076113.

Genotyping for the CIDRUK samples were provided by the Center for Inherited Disease Research (CIDR). CIDR is fully funded through a federal contract from the National Institutes of Health to The Johns Hopkins University, contract number HHSN268201200008I.

Funding specific to particular centers is given below:

Stockholm: Swedish Cancer Society, Karolinska Institutet Research Funds, Radiumhemmet Research Funds, Stockholm County Council Research Funding (ALF).

Lund: Funding to be acknowledged; Swedish Cancer Society, Gunnar Nilsson Foundation, and European Research Council Advanced Grant (ERC-2011–294576).

Genoa: Italian Ministry of Education, University and Research PRIN 2008, IMI and Mara Naum foundation. Italian association for cancer research (AIRC) IG 2014 (15460) to PG; IRCCS AOU San Martino-IST Istituto Nazionale per la Ricerca sul Cancro, 5% per la ricerca corrente, to PG and GBS.

Leiden: Grant provided by European Biobanking and Biomolecular Resources Research Infrastructure (BBMRI) −Netherlands hub (CO18).

Spain: The research at the Melanoma Unit in Barcelona is or was partially funded by Grants from Fondo de Investigaciones Sanitarias P.I. 09/01393 & 12/00840, Spain; by the CIBER de Enfermedades Raras of the Instituto de Salud Carlos III, Spain; by the AGAUR 2009 SGR 1337 and AGAUR 2014_SGR_603 of the Catalan Government, Spain; by a grant from “Fundació La Marató de TV3, 201331-30”, Catalonia, Spain; by the European Commission under the 6th Framework Programme, Contract nº: LSHC-CT-2006-018702 (GenoMEL) and by the National Cancer Institute (NCI) of the US National Institute of Health (NIH) (CA83115).

Norway: Grants from the Comprehensive Cancer Center, Oslo University Hospital (SE0728) and the Norwegian Cancer Society (71512-PR-2006-0356).

**AMFS**

The AMFS was supported by the National Health and Medical Research Council of Australia (NHMRC) (project grants 566946, 107359, 211172 and program grant number 402761 to GJM and RFK); the Cancer Council New South Wales (project grant 77/00, 06/10), the Cancer Council Victoria and the Cancer Council Queensland (project grant 371); and the US National Institutes of Health (NIH RO1 grant CA-83115-01A2 and 2R01CA083115-11A1 to the international Melanoma Genetics Consortium - GenoMEL). Anne E. Cust is supported by fellowships from the Cancer Institute NSW and the NHMRC. We gratefully acknowledge all of the participants, and the work and dedication of the research coordinators, interviewers, examiners and data management staff.

**WAMHS**

The WAMHS gratefully acknowledges all study participants for their time and contributions, and the Western Australian DNA Bank and the Ark at The University of Western Australia for biospecimen and bioinformatics related support. The Western Australian Cancer Registry, the WAMHS study team and the WAMHS Management Committee are also gratefully acknowledged for their assistance, as well as the Scott Kirkbride Melanoma Research Centre for funding received to establish the WAMHS resource and related salaries and PhD stipends. The Cancer Council Western Australia is also acknowledged for current salary support for Sarah Ward (Capacity Building and Collaboration grant).

Genotyping services were provided by the Center for Inherited Disease Research (CIDR). CIDR is fully funded through a federal contract from the National Institutes of Health to The Johns Hopkins University, contract number HHSN268201200008I’

**Q-MEGA cases and QTWINs controls (used in Q-MEGA_610k set)**

Acknowledgement/grants: Q-MEGA and QTWIN thanks A. Baxter, M. de Nooyer, I. Gardner, D. Statham, B. Haddon, M.J. Wright, J. Palmer, J. Symmons, B. Castellano, L. Bardsley, S. Smith, D. Smyth, L. Wallace, M.J. Campbell, A. Caracella, M. Kvaskoff, O. Zheng, B. Chapman and H. Beeby for their input in project management, sample processing and database development. We are grateful to the many research assistants and interviewers for assistance with the studies contributing to the QMEGA and QTWIN collections

The Q-MEGA/QTWIN study was supported by the Melanoma Research Alliance, the NIH NCI (CA88363, CA83115, CA122838, CA87969, CA055075, CA100264, CA133996 and CA49449), the National Health and Medical Research Council of Australia (NHMRC) (200071, 241944, 339462, 380385, 389927,389875, 389891, 389892,389938, 443036, 442915, 442981, 496610, 496675, 496739, 552485, 552498), the Cancer Councils New South Wales, Victoria and Queensland, the Cancer Institute New South Wales, the Cooperative Research Centre for Discovery of Genes for Common Human Diseases (CRC), Cerylid Biosciences (Melbourne), the Australian Cancer Research Foundation, The Wellcome Trust (WT084766/Z/08/Z) and donations from Neville and Shirley Hawkins. Stuart MacGregor acknowledges fellowship support from the Australian National Health and Medical Research Council and from the Australian Research Council.

# References

1. Burgess S, Thompson SG. Bias in causal estimates from Mendelian randomization studies with weak instruments. Stat Med [Internet]. 2011 May 20;30(11):1312–23. Available from: http://www.ncbi.nlm.nih.gov/pubmed/21432888

2. Burgess S, Davies NM, Thompson SG. Bias due to participant overlap in two‐sample Mendelian randomization. Genet Epidemiol [Internet]. 2016 Nov 1 [cited 2022 Feb 25];40(7):597. Available from: /pmc/articles/PMC5082560/

3. Auton A, Abecasis GR, Altshuler DM, Durbin RM, Bentley DR, Chakravarti A, et al. A global reference for human genetic variation [Internet]. Vol. 526, Nature. Nature Publishing Group; 2015 [cited 2020 Dec 23]. p. 68–74. Available from: https://www.nature.com/articles/nature15393

4. Shin S-Y, Fauman EB, Petersen A-K, Krumsiek J, Santos R, Huang J, et al. An atlas of genetic influences on human blood metabolites. Nat Genet [Internet]. 2014 Jun 11 [cited 2019 Mar 2];46(6):543–50. Available from: http://www.ncbi.nlm.nih.gov/pubmed/24816252

5. Dorajoo R, Sun Y, Han Y, Ke T, Burger A, Chang X, et al. A genome-wide association study of n-3 and n-6 plasma fatty acids in a Singaporean Chinese population. Genes Nutr [Internet]. 2015 Nov 19 [cited 2019 Mar 2];10(6):53. Available from: http://link.springer.com/10.1007/s12263-015-0502-2

6. Kettunen J, Demirkan A, Würtz P, Draisma HHM, Haller T, Rawal R, et al. Genome-wide study for circulating metabolites identifies 62 loci and reveals novel systemic effects of LPA. Nat Commun [Internet]. 2016 Jan 23 [cited 2016 Mar 24];7(1):11122. Available from: http://www.nature.com/articles/ncomms11122

7. Tintle NL, Pottala J V., Lacey S, Ramachandran V, Westra J, Rogers A, et al. A genome-wide association study of saturated, mono- and polyunsaturated red blood cell fatty acids in the Framingham Heart Offspring Study. Prostaglandins Leukot Essent Fat Acids [Internet]. 2015 Mar [cited 2019 Mar 2];94:65–72. Available from: https://linkinghub.elsevier.com/retrieve/pii/S0952327814001987

8. Hemani G, Zheng J, Elsworth B, Wade KH, Haberland V, Baird D, et al. The MR-Base platform supports systematic causal inference across the human phenome. Elife [Internet]. 2018 May 30 [cited 2019 Mar 1];7. Available from: https://elifesciences.org/articles/34408

9. Elsworth​ B, Lyon​ M, Alexander​ T, Liu​ Y, Matthews​ P, Hallett​ J, et al. The MRC IEU OpenGWAS data infrastructure. bioRxiv [Internet]. 2020 Aug 10 [cited 2021 Nov 11];2020.08.10.244293. Available from: https://www.biorxiv.org/content/10.1101/2020.08.10.244293v1

10. Han B, Duong D, Sul JH, de Bakker PIW, Eskin E, Raychaudhuri S. A general framework for meta-analyzing dependent studies with overlapping subjects in association mapping. Hum Mol Genet [Internet]. 2016 May 1 [cited 2022 Mar 1];25(9):1857–66. Available from: https://pubmed.ncbi.nlm.nih.gov/26908615/

11. Lin DY, Sullivan PF. Meta-Analysis of Genome-wide Association Studies with Overlapping Subjects. Am J Hum Genet [Internet]. 2009 Dec 11 [cited 2022 Mar 1];85(6):862. Available from: /pmc/articles/PMC2790578/

12. Giambartolomei C, Vukcevic D, Schadt EE, Franke L, Hingorani AD, Wallace C, et al. Bayesian test for colocalisation between pairs of genetic association studies using summary statistics. Williams SM, editor. PLoS Genet [Internet]. 2014 May 15 [cited 2014 Jul 12];10(5):e1004383. Available from: http://dx.plos.org/10.1371/journal.pgen.1004383

13. Consortium TGte. The GTEx Consortium atlas of genetic regulatory effects across human tissues. Science [Internet]. 2020 Sep 11 [cited 2020 Oct 8];369(6509):1318–30. Available from: http://science.sciencemag.org/

14. Võsa U, Claringbould A, Westra H-J, Bonder MJ, Deelen P, Zeng B, et al. Unraveling the polygenic architecture of complex traits using blood eQTL metaanalysis. bioRxiv [Internet]. 2018 Oct 19 [cited 2020 Oct 8];447367. Available from: https://europepmc.org/article/PPR/PPR59262

15. Haycock PC, Carolina Borges M, Burrows K, Lemaitre RN, Harrison S, Burgess S, et al. Design and quality control of large-scale two-sample Mendelian randomisation studies. medRxiv [Internet]. 2021 Aug 1 [cited 2021 Nov 11];2021.07.30.21260578. Available from: https://www.medrxiv.org/content/10.1101/2021.07.30.21260578v1

16. Howe LJ, Nivard MG, Morris TT, Hansen AF, Rasheed H, Cho Y, et al. Within-sibship GWAS improve estimates of direct genetic effects. bioRxiv [Internet]. 2021 Mar 7 [cited 2021 Nov 11];2021.03.05.433935. Available from: https://www.biorxiv.org/content/10.1101/2021.03.05.433935v1

17. Brumpton B, Sanderson E, Heilbron K, Hartwig FP, Harrison S, Vie GÅ, et al. Avoiding dynastic, assortative mating, and population stratification biases in Mendelian randomization through within-family analyses. Nat Commun 2020 111 [Internet]. 2020 Jul 14 [cited 2021 Nov 11];11(1):1–13. Available from: https://www.nature.com/articles/s41467-020-17117-4

18. Elsworth​ B, Lyon​ M, Alexander​ T, Liu​ Y, Matthews​ P, Hallett​ J, et al. The MRC IEU OpenGWAS data infrastructure. bioRxiv [Internet]. 2020 Aug 10 [cited 2020 Dec 23]; Available from: https://doi.org/10.1101/2020.08.10.244293

19. Markozannes G, Kanellopoulou A, Dimopoulou O, Kosmidis D, Zhang X, Wang L, et al. Systematic review of Mendelian randomization studies on risk of cancer. BMC Med [Internet]. 2022 Dec 1 [cited 2022 Apr 2];20(1):1–22. Available from: https://bmcmedicine.biomedcentral.com/articles/10.1186/s12916-022-02246-y

20. Carter AR, Sanderson E, Hammerton G, Richmond RC, Davey Smith G, Heron J, et al. Mendelian randomisation for mediation analysis: current methods and challenges for implementation. Eur J Epidemiol [Internet]. 2021 May 1 [cited 2022 Feb 4];36(5):465–78. Available from: https://link.springer.com/article/10.1007/s10654-021-00757-1

21. Lewandowski RJ, Salem R, Mouli SK, Karp JK, Laws JL, Ryu RK, et al. Deaths Due to Cigarette Smoking for 12 Smoking-Related Cancers in the United States [Internet]. Vol. 175, JAMA Internal Medicine. 2015 [cited 2020 Oct 27]. p. 1574–6. Available from: https://www.ncbi.nlm.nih.gov/books/NBK294317/table/ch4.t1/

22. Table 4.1, Conclusions from Surgeon General’s report on active cigarette smoking and cancer [Internet]. Centers for Disease Control and Prevention (US); 2014 [cited 2020 Oct 27]. Available from: https://www.ncbi.nlm.nih.gov/books/NBK294317/table/ch4.t1/

23. Coussens LM, Werb Z. Inflammation and cancer [Internet]. Vol. 420, Nature. NIH Public Access; 2002 [cited 2020 Nov 2]. p. 860–7. Available from: /pmc/articles/PMC2803035/?report=abstract

24. Surveillance, Epidemiology, and End Results Program [Internet]. [cited 2021 Nov 11]. Available from: https://seer.cancer.gov/

25. Tomasetti C, Vogelstein B. Cancer etiology. Variation in cancer risk among tissues can be explained by the number of stem cell divisions. Science [Internet]. 2015 Jan 2 [cited 2021 Nov 11];347(6217):78–81. Available from: https://pubmed.ncbi.nlm.nih.gov/25554788/

26. Viechtbauer W. Conducting meta-analyses in R with the metafor. J Stat Softw. 2010;36(3):1–48.

27. Viechtbauer W. Conducting Meta-Analyses in R with the metafor Package. J Stat Softw [Internet]. 2010 Aug 5 [cited 2022 Mar 1];36(3):1–48. Available from: https://www.jstatsoft.org/index.php/jss/article/view/v036i03

28. Rahbar E, Ainsworth HC, Howard TD, Hawkins GA, Ruczinski I, Mathias R, et al. Uncovering the DNA methylation landscape in key regulatory regions within the FADS cluster. PLoS One [Internet]. 2017 Sep 1 [cited 2022 Aug 18];12(9). Available from: https://pubmed.ncbi.nlm.nih.gov/28957329/

29. Nishida N, Yano H, Nishida T, Kamura T, Kojiro M. Angiogenesis in Cancer. Vasc Health Risk Manag [Internet]. 2006 [cited 2022 Aug 18];2(3):213. Available from: /pmc/articles/PMC1993983/

30. Gharahkhani P, Fitzgerald RC, Vaughan TL, Palles C, Gockel I, Tomlinson I, et al. Genome-wide association studies in oesophageal adenocarcinoma and Barrett’s oesophagus: a large-scale meta-analysis. Lancet Oncol. 2016 Oct;17(10):1363–73.
